# Measurement errors in control risk regression: A comparison of correction techniques

**DOI:** 10.1002/sim.9228

**Published:** 2021-10-15

**Authors:** Annamaria Guolo

**Affiliations:** ^1^ Department of Statistical Sciences University of Padova Padova Italy

**Keywords:** likelihood, measurement error, meta‐analysis, score function, SIMEX

## Abstract

Control risk regression is a diffuse approach for meta‐analysis about the effectiveness of a treatment, relating the measure of risk with which the outcome occurs in the treated group to that in the control group. The severity of illness is a source of between‐study heterogeneity that can be difficult to measure. It can be approximated by the rate of events in the control group. Since the estimate is a surrogate for the underlying risk, it is prone to measurement error. Correction methods are necessary to provide reliable inference. This article illustrates the extent of measurement error effects under different scenarios, including departures from the classical normality assumption for the control risk distribution. The performance of different measurement error corrections is examined. Attention will be paid to likelihood‐based structural methods assuming a distribution for the control risk measure and to functional methods avoiding the assumption, namely, a simulation‐based method and two score function methods. Advantages and limits of the approaches are evaluated through simulation. In case of large heterogeneity, structural approaches are preferable to score methods, while score methods perform better for small heterogeneity and small sample size. The simulation‐based approach has a satisfactory behavior whichever the examined scenario, with no convergence issues. The methods are applied to a meta‐analysis about the association between diabetes and risk of Parkinson disease. The study intends to make researchers aware of the measurement error problem occurring in control risk regression and lead them to the use of appropriate correction techniques to prevent fallacious conclusions.

## 1 INTRODUCTION

Meta‐analysis instruments are commonly adopted to evaluate the effectiveness of a treatment in clinical trials comparing a treatment group and a control group.^1^, ^2^ Detecting and explaining heterogeneity between the studies are crucial, as heterogeneity can be a consequence of several factors, including differences in study designs, characteristics of patients enrolled, and clinical interventions. Although many sources of study heterogeneity can be quantified, some factors can be difficult to measure, as, for example, the severity of illness in patients.^3^ An approximation of the severity of illness is given by the underlying risk or baseline risk for the patients in the control condition, a measure of the rate at which the outcome of interest occurs. This kind of information is typically not available at the population level, but it can be measured from the studies included in the meta‐analysis through the control rate, that is, the rate of events in the control group.^1^, ^2^, ^3^, ^4^, ^5^, ^6^ The inclusion of such information in the meta‐analysis model gives rise to the so‐called control risk regression. Control risk regression is an example of meta‐regression,^1^ an extension of meta‐analysis obtained through the inclusion of study‐specific covariates useful to quantity the contribution of the differences among the studies to the overall heterogeneity. Criticism toward the use of meta‐regression points out the risk of low power to detect relationships or of data dredging.^7^ Nevertheless, meta‐regression is commonly adopted as an efficient alternative to the simple subgrouping of studies with different characteristics.^8^, ^9^ The use of a surrogate for the measure of risk of the outcome from the studies included in the meta‐analysis suggests that the available information is affected by error. The most known effect of measurement error is the attenuation bias, that is, a biased toward zero estimate of the estimator of the coefficient associated to the risk measure in control risk regression, under an additive and homoscedastic error on the baseline risk measure, see, for example, van Houwelingen et al.^1^ This article investigates the effects of measurement errors in inferential procedures including estimates of the parameters of interest in control risk regression, the evaluation of the associated variability, and the construction of confidence intervals. Attention is paid to classical scenarios where normality is assumed for the control risk distribution and to nonclassical scenarios with departures from the normality assumption. Correcting for the presence of measurement error is a necessary step for inference to be reliable. The last decade has seen the development of several approaches to face the measurement error problem in control risk regression. Proposals are primarily inspired by solutions developed in the long‐established measurement error literature,^10^, ^11^, ^12^, ^13^ which distinguishes between structural techniques, when a distribution for the mismeasured covariate is assumed, and functional techniques, that are free of assumptions on the mismeasured covariates. A comparison of some of the proposed correction methods has been carried out in Ghidey et al,^14^ namely, a structural solution represented by the likelihood approach under a Normal distribution for the underlying risk,^15^, ^16^, ^17^ the method of moments, and two functional methods, represented by conditional score and corrected score functions.^11^ The comparison performed through simulation includes the underlying risk following a Normal distribution or a mixture of Normals.

The article intends to deeply investigate the performance of the correction methods in control risk regression under different scenarios, with the aim of providing suggestions to choose the appropriate solution, in this way avoiding the risk of fallacious conclusions. The article extends the previous comparison carried out in Ghidey et al^14^ by including other measurement error solutions beyond the functional methods. Namely, the article considers the likelihood‐based approach when the classical Normal distribution for the control risk measure is assumed and when a flexible alternative represented by the Skew‐Normal distribution is adopted^17^ and a simulation based‐technique.^18^ All the approaches are compared to the uncorrected weighted least squares regression ignoring the presence of measurement errors. The comparison is performed through simulation, including the underlying risk following a Normal distribution, a Skew‐Normal distribution or a mixture of Normals, in scenarios with increasing sample size and between‐study heterogeneity. In addition, data are simulated according to a two‐step procedure with the aim of reflecting the control risk generation mechanism, instead of the usual simulation strategy which samples data directly from the model considered in the meta‐analysis.

Characteristics of the competing approaches, either in terms of accuracy of inferential procedures and applicability with interest on convergence issues, are discussed. The methods are illustrated in a real data example about the association between diabetes and risk of Parkinson disease.

## 2 BACKGROUND

Consider a meta‐analysis of *n* independent studies comparing a treated group and a control group, with the aim of evaluating the effectiveness of a common treatment. Let $\eta_{i}$ and $\xi_{i},i=1,\ldots ,n,$ denote the true unobserved measure of risk in the treatment group and the true unobserved measure of risk in the control group for study *i*, respectively. Common measures of risk are the log‐odds or the log‐event rate. The true unobserved measure of risk in the control group $\xi_{i}$ is often called control risk or underlying risk.^2^ A well‐established and computationally convenient model relating $\eta_{i}$ and $\xi_{i}$ is the linear regression model^2^, ^5^, ^15^, ^19^

(1) $$\eta_{i}=\beta_{0}+\beta_{1}\xi_{i}+\varepsilon_{i},\;\varepsilon_{i}∼N(0,\tau^{2}),$$

where $\tau^{2}$ is the residual variance describing the variation among studies in the treatment rate unexplained by the underlying risk, that is, due to factors different from the severity of illness. Let the sampling error $\varepsilon_{i}$ be independent of $\xi_{i}$. Parameter $\beta_{1}$ represents the interest of the analysis, as it describes the relationship between the treatment rate and the underlying risk. The case $\beta_{1}=0$ indicates a treatment rate independent of the underlying risk, that is, a constant treatment rate equal to $\beta_{0}$, while the case $\beta_{1}=1$ indicates that when the risk in the control condition increases by a given amount, then the risk in the treatment group increases by the same amount. A different model can be formulated that considers the relationship between the treatment effect and the underlying risk. If the treatment effect is $\eta_{i}-\xi_{i}$, then model

$$\eta_{i}-\xi_{i}=\beta_{0}^{*}+\beta_{1}^{*}\xi_{i}+\varepsilon_{i},\;\varepsilon_{i}∼N(0,\tau^{2})$$

is a reparameterization of model (1), with ${\left(\beta_{0}^{*},\beta_{1}^{*}\right)}^{⊤}={\left(0,0\right)}^{⊤}$ being an indication of no treatment effect, see Ghidey et al.^14^ Increasing values of $\beta_{1}^{*}$ reflect a larger treatment effect for subjects with larger risks. Model formulation (1) has the advantage of independence between the risk measure in the treatment group and the risk measure in the control group, as they are computed on different subjects. The alternative formulation, conversely, gives rise to dependence between the measure of the treatment effect and the control risk, with the possibility of spurious correlation, see van Houwelingen et al^1^ and references therein. In the rest of this article, we will focus on relationship given in model (1). Inference is typically performed using the estimates ${\hat{\eta }}_{i}$ and ${\hat{\xi }}_{i}$ of $\eta_{i}$ and $\xi_{i}$, respectively, obtained as summary measures provided by each study. The simplest approach for analysis^4^ is a weighted least squares regression, with weights given by the inverse of the variance of the treatment rate. Large criticism toward the weighted least squares regression highlights that the approach does not account for the measurement error affecting ${\hat{\eta }}_{i}$ and ${\hat{\xi }}_{i}$ as they are estimated rather than true values obtained from each study included in the meta‐analysis in form of summary measures.^15^ The main consequence of measurement error in both the variables of model (1) has been long recognized an estimate of $\beta_{1}$ biased toward zero.^1^, ^20^ Actually, there are further effects of measurement errors on inferential conclusions, as it would be illustrated in the simulation studies. Over the past few decades, a huge literature has focused on the effects of measurement errors affecting covariates in regression models. Notable examples are included in the book‐length reviews of measurement error correction techniques by, for example, Gustafson,^10^ Carroll et al,^11^ Buonaccorsi,^12^ and Yi,^13^ see also Keogh et al^21^ and Shaw et al.^22^ Consequences of ignoring the errors in variables are various, with negligible to substantial effects on inferential conclusions. The most known effect is the *attenuation phenomenon*, that is, a biased toward zero estimate of the slope in simple linear regression model when the covariate is mismeasured. The attenuation phenomenon occurs in case the measurement error affecting the covariate is classical and additive, that is, the observed measure $X^{*}$ is the sum of the true unobserved covariate *X* plus an error component *U*, where *U* is independent of *X*, with zero mean and constant variance, for example, Buonaccorsi.^12^ ^(Chapter5)^. Outside this situation, as, for example, in case of nonlinear models or more complex error structures, effects are unpredictable (eg, Carroll et al^11^ ^(Chapter3)^) and typically include reduced power of tests and empirical coverage probabilities far from the nominal level, usually underestimating it. When the unbiased and homoscedastic measurement error affects the response variable, consequences in linear regression models do not typically include bias of the estimators. The main effect is an increased variability of the observed data about the least squares fitted line if compared to the error‐free data. Inferential procedures such as tests and confidence intervals remain valid, although less powerful. See, for example, Carroll et al.^11^ ^(Chapter15)^

In control risk regression, information available from each study included in the meta‐analysis, either ${\hat{\eta }}_{i}$ or ${\hat{\xi }}_{i}$, is a summary measure of the outcome risk for the subjects belonging to that study. Accordingly, the measure is an estimate of the real $\eta_{i}$ or $\xi_{i}$ affected by the variability associated to the estimation process. In contrast to much of the literature focused on mismeasured covariates, measurement errors in control risk regression affect both the covariate and the response, as Ghidey et al^14^ point out. Issues in this framework have been relatively less explored and typically refer to situations where the mismeasured variables are independent, for example, Buonaccorsi.^12^ ^(Chapter4)^ In control risk regression, conversely, we cannot assume that the error in $\eta_{i}$ and the error in $\xi_{i}$ are independent, as they can reflect characteristics of the measurement procedure in the patients enrolled in the same study included in the meta‐analysis.

## 3 CORRECTION METHODS

Consider the measurement error model specifying the relationship between error‐prone estimates ${\left({\hat{\eta }}_{i},{\hat{\xi }}_{i}\right)}^{⊤}$ based on study *i* and the corresponding error‐free unobserved variables ${\left(\eta_{i},\xi_{i}\right)}^{⊤}$. A common choice is the bivariate Normal specification, namely,

(2) $$\left.\left(\begin{array}{c} {\hat{\eta }}_{i}\\ {\hat{\xi }}_{i} \end{array}\right)\right|\left(\begin{array}{c} \eta_{i}\\ \xi_{i} \end{array}\right)∼{\text{Normal}}_{2}\left(\left(\begin{array}{c} \eta_{i}\\ \xi_{i} \end{array}\right),\Gamma_{i}\right),$$

where the within‐study variance/covariance matrix $\Gamma_{i}$ is specified using single study information. Model (2) assumes that ${\left({\hat{\eta }}_{i},{\hat{\xi }}_{i}\right)}^{⊤}$ is an unbiased measure of the unknown ${\left(\eta_{i},\xi_{i}\right)}^{⊤}$, with an additive component accounting for residual variation, due, for example, to study characteristics in observational studies. In the rest of this article, the within‐study variance/covariance matrix $\Gamma_{i}$ will be considered as known and denoted by ${\hat{\Gamma }}_{i}$. Matrix ${\hat{\Gamma }}_{i}$ is equal to that obtained from the information of each study. Considering the within‐study variance‐covariance matrix as known, when it is actually estimated from each study, is a common and convenient assumption in classical meta‐analysis, usually justified by large sample size of each study in medical or epidemiological investigations. See Hamza et al^23^ and Bellio and Guolo^24^ for a discussion and innovative solutions in case the assumption is not satisfied.

### 3.1 Structural methods

The structural approach to measurement error correction in terms of likelihood function requires the specification of the distribution for the underlying risk in the control condition $\xi_{i}$. Let $p_{i}(\xi_{i};\delta )$ be the associated density function, with parameter vector δ. Let $p_{i}(\eta_{i}|\xi_{i};\psi )$ be the density function for the conditional outcome of the control risk regression model (1), with $\psi ={\left(\beta_{0},\beta_{1},\tau^{2}\right)}^{⊤}$, and let $p_{i}({\hat{\eta }}_{i},{\hat{\xi }}_{i}|\eta_{i},\xi_{i};\gamma_{i})$ be the density function for the conditional outcome of the measurement error model (2), depending on the study‐specific vector of parameters $\gamma_{i}$ which consists of the known elements of ${\hat{\Gamma }}_{i}$. The likelihood function for the whole parameter vector $\theta ={\left(\psi^{⊤},\delta^{⊤}\right)}^{⊤}$ is

$$L(\theta )=\overset{n}{\underset{i=1}{\prod }}\;\int \int p_{i}({\hat{\eta }}_{i},{\hat{\xi }}_{i}|\eta_{i},\xi_{i};\gamma_{i})p_{i}(\eta_{i}|\xi_{i};\psi )p_{i}(\xi_{i};\delta )d\eta_{i}d\xi_{i}.$$

For computational convenience, a Normal distribution for $\xi_{i}$ is usually assumed $\xi_{i}∼N(\mu ,\sigma^{2})$, so that $\delta ={\left(\mu ,\sigma^{2}\right)}^{⊤}$. This leads to a closed‐form expression of L(θ) (eg, Guolo^17^),

$$L(\theta )=\overset{n}{\underset{i=1}{\prod }}\;p_{i}({\hat{\eta }}_{i},{\hat{\xi }}_{i};\theta ),$$

where $p_{i}({\hat{\eta }}_{i},{\hat{\xi }}_{i};\theta )$ is the density function of a bivariate Normal distribution

$$\left(\begin{array}{c} {\hat{\eta }}_{i}\\ {\hat{\xi }}_{i} \end{array}\right)∼{\text{Normal}}_{2}\left(\left(\begin{array}{c} \beta_{0}+\beta_{1}\mu \\ \mu \end{array}\right),\;{\hat{\Gamma }}_{i}+\left[\begin{array}{cc} \tau^{2}+\beta_{1}^{2}\sigma^{2} & \beta_{1}\sigma^{2}\\ \beta_{1}\sigma^{2} & \sigma^{2} \end{array}\right]\right).$$

Despite the computational advantage of a Normal specification for the underlying risk distribution, the choice does not allow to account for common forms of non‐normality arising in applications, including skewness, bimodality, heavy tails, see for example, Guolo^17^ and Lee and Thompson.^25^ Specifications of the underlying risk distribution other than the Normal have been proposed in the literature. They include flexible solutions based on mixture of Normals,^15^ semiparametric specification,^16^ and the Skew‐Normal distribution.^17^ While the proposals are shown to be satisfactory in terms of improved inference on variance components, computational difficulties related to the loss of the closed‐form likelihood function can discourage the applicability. In this article, we will focus on the Skew‐Normal specification illustrated in Guolo^17^ to deal with deviations from normality due to skewness. The approach has a feasible implementation and simulation studies in Reference 17 suggest that it does not suffer from numerical instabilities. Moreover, in case the underlying risk is actually normally distributed, then the Skew‐Normal specification does not misrepresent the data as it includes the Normal distribution as a special case. Finally, the choice does not limit the standard likelihood theory to be applied.

Consider the underlying risk $\xi_{i}$ following the Skew‐Normal distribution,^26^ $\xi_{i}∼SN(\mu ,\sigma ,\alpha )$, with density function

(3) $$p_{\xi_{i}}(\xi_{i};\delta )=p_{\xi_{i}}(\xi_{i};\mu ,\sigma ,\alpha )=(2/\sigma )\phi \{(\xi_{i}-\mu )/\sigma \}\Phi \{\alpha (\xi_{i}-\mu )/\sigma \},$$

where $\delta ={\left(\mu ,\sigma ,\alpha \right)}^{⊤}$, μ,σ,α denote, respectively, the location, the scale, and the shape parameter, and ϕ(·) and Φ(·) represent the standard Normal density and distribution functions, respectively. The likelihood function

(4) $$L(\theta )=\overset{n}{\underset{i=1}{\prod }}\;\int_{\mathbb{R}}p({\hat{\eta }}_{i},{\hat{\xi }}_{i}|\xi_{i};\gamma ,{\hat{\Gamma }}_{i})p(\xi_{i};\delta )d\xi_{i}$$

does not have a closed‐form expression and a numerical integration is needed, for example, via a Gauss‐Hermite quadrature. Then, the maximum likelihood estimate (MLE) $\hat{\theta }$ of the whole parameter vector θ can be obtained by optimization routines in standard softwares.

In order to account for model misspecification, especially in terms of uncorrected specification of the distribution for $\xi_{i}$, the standard errors of the MLE can be obtained using the sandwich formula, see for example, Guolo.^17^ Let $\ell_{i}(\theta )$ be the log‐likelihood contribution of study *i*. The sandwich estimator of the variance‐covariance matrix of $\hat{\theta }$ is

$$\text{cov}(\hat{\theta })=n^{-1}J^{-1}(\theta )I(\theta )J^{-1}(\theta ){|}_{\theta =\hat{\theta }},$$

where

$$J(\theta )=n^{-1}\overset{n}{\underset{i=1}{\sum }}\;\frac{\partial^{2}}{\partial \theta \partial \theta^{𝒯}}\ell_{i}(\theta )$$

and

$$I(\theta )=n^{-1}\overset{n}{\underset{i=1}{\sum }}\;\frac{\partial }{\partial \theta }\ell_{i}(\theta ){\left\{\frac{\partial }{\partial \theta }\ell_{i}(\theta )\right\}}^{𝒯}.$$

See, for example, Carroll et al.^27^ ^(SectionA.6.1)^

### 3.2 Functional methods

Differently from structural approaches, functional methods for measurement error correction do not make any assumption on the distribution of the unobserved underlying risk $\xi_{i}$. According to this view, parameters $\xi_{1},\ldots ,\xi_{n}$ are interpreted as additional nuisance components to be estimated. Thus, the total number of parameters increases with the sample size, giving rise to a framework where standard likelihood inference might fail, see for example, Severini.^28^ The use of functional methods has the advantage of robustness of the inferential conclusions to misspecification of the control risk distribution, although at the price of a loss of efficiency of the results with respect to fully parametric solutions.

#### 3.2.1 Score functions

In order to face the problem of increasing number of parameters, Ghidey et al^14^ refer to the methodology of unbiased estimating equations, by investigating two approaches developed in the measurement error literature and known as *corrected score approach* and *conditional score approach*. Both methods produce consistent estimators that are *M*‐estimators, whose score function is unbiased in presence of measurement error, see also Carroll et al.^11^ ^(Chapter7)^

Starting from the estimating equations for ${\left(\beta_{0},\beta_{1},\tau^{2}\right)}^{⊤}$, Ghidey et al^14^ derive the weighted estimating equations accounting for the size of the measurement error in study *i*. Let $\sigma_{{\hat{\eta }}_{i}}^{2}$ and $\sigma_{{\hat{\xi }}_{i}}^{2}$ denote the known variance of ${\hat{\eta }}_{i}$ and ${\hat{\xi }}_{i}$, respectively. Then, the corrected score equations for ${\left(\beta_{0},\beta_{1},\tau^{2}\right)}^{⊤}$ are

$$\begin{array}{ll} & \overset{n}{\underset{i=1}{\sum }}\;\frac{{\hat{\eta }}_{i}-\beta_{0}-\beta_{1}{\hat{\xi }}_{i}}{\sigma_{{\hat{\eta }}_{i}}^{2}+\tau^{2}}=0,\\ & \overset{n}{\underset{i=1}{\sum }}\;\frac{\left({\hat{\eta }}_{i}-\beta_{0}-\beta_{1}{\hat{\xi }}_{i}\right){\hat{\xi }}_{i}+\beta_{1}\sigma_{{\hat{\xi }}_{i}}^{2}}{\sigma_{{\hat{\eta }}_{i}}^{2}+\tau^{2}}=0,\\ & \overset{n}{\underset{i=1}{\sum }}\;\frac{\sigma_{{\hat{\eta }}_{i}}^{2}+\tau^{2}+\beta_{1}^{2}\sigma_{{\hat{\xi }}_{i}}^{2}-{\left({\hat{\eta }}_{i}-\beta_{0}-\beta_{1}{\hat{\xi }}_{i}\right)}^{2}}{{\left(\sigma_{{\hat{\eta }}_{i}}^{2}+\tau^{2}\right)}^{2}}=0. \end{array}$$

The corrected estimates of ${\left(\beta_{0},\beta_{1},\tau^{2}\right)}^{⊤}$ can be obtained through a Newton‐Raphson algorithm and the associated standard error from the sandwich formula. The consistency of the resulting estimator is based only on the correct specification of the first and second moments of the sampling error $\varepsilon_{i}$, without any distributional assumption on $\xi_{i}$.

The conditional score approach developed in Stefanski and Carroll^29^ makes use of sufficient statistics to derive the parameters estimators. Ghidey et al^14^ obtain the conditional score equations for ${\left(\beta_{0},\beta_{1},\tau^{2}\right)}^{⊤}$, namely,

$$\begin{array}{ll} & \overset{n}{\underset{i=1}{\sum }}\;\frac{{\hat{\eta }}_{i}-\beta_{0}-\beta_{1}{\hat{\xi }}_{i}}{\sigma_{{\hat{\eta }}_{i}}^{2}+\tau^{2}}=0,\\ & \overset{n}{\underset{i=1}{\sum }}\;\frac{\left({\hat{\eta }}_{i}-\beta_{0}-\beta_{1}{\hat{\xi }}_{i}\right){\hat{\xi }}_{i}}{\sigma_{{\hat{\eta }}_{i}}^{2}+\tau^{2}}+\frac{\beta_{1}\sigma_{{\hat{\xi }}_{i}}^{2}{\left({\hat{\eta }}_{i}-\beta_{0}-\beta_{1}{\hat{\xi }}_{i}\right)}^{2}}{\left(\sigma_{{\hat{\eta }}_{i}}^{2}+\tau^{2}\right)\left(\sigma_{{\hat{\eta }}_{i}}^{2}+\tau^{2}+\beta_{1}^{2}\sigma_{{\hat{\xi }}_{i}}^{2}\right)}=0,\\ & \overset{n}{\underset{i=1}{\sum }}\;\frac{\sigma_{{\hat{\eta }}_{i}}^{2}+\tau^{2}+\beta_{1}^{2}\sigma_{{\hat{\xi }}_{i}}^{2}-{\left({\hat{\eta }}_{i}-\beta_{0}-\beta_{1}{\hat{\xi }}_{i}\right)}^{2}}{{\left(\sigma_{{\hat{\eta }}_{i}}^{2}+\tau^{2}\right)}^{2}}=0. \end{array}$$

As in the previous approach, the equations can be solved through a Newton‐Raphson algorithm and the associated standard error can be obtained through the sandwich formula.

Simulation studies in Ghidey et al^14^ suggest that the approaches perform similarly when the measurement error is small. When the measurement error is large and the number of studies recruited in the meta‐analysis is small, the conditional score approach is preferable as the associated estimator is more efficient.^14^

#### 3.2.2 Simulation‐extrapolation

SIMEX is a simulation‐based functional approach developed to estimate and reduce the effects of measurement errors affecting covariates, see Cook and Stefanski^30^ and Stefanski and Cook.^31^ The method has been originally developed to correct for additive errors, but it can be extended to any situation where the measurement error can be simulated via Monte Carlo procedures. SIMEX consists of two steps. In the first step, simulation is used to estimate the parameters in datasets generated with additional increasing measurement errors. In the second step, the relationship between the estimates and the amount of the added measurement error is established and used to extrapolate the estimate back to the case of no measurement error. As the approach focuses on the main model (1), its applicability with existing software is straightforward, in this way making it extremely appealing.^17^

Let $W_{i}={\left({\hat{\eta }}_{i},{\hat{\xi }}_{i}\right)}^{⊤}$ denote the vector of data from study *i*, with expected value $X_{i}={\left(\eta_{i},\xi_{i}\right)}^{⊤}$ and known variance/covariance matrix ${\hat{\Gamma }}_{i}$ and let $\psi ={\left(\beta_{0},\beta_{1},\tau^{2}\right)}^{⊤}$ denote the vector of parameters in model (1). In the *simulation step*, for any λ≥0 in a grid $\Lambda =\{0,\lambda_{1},\ldots ,\lambda_{M}\}$, additional independent measurement errors are generated *B* times starting from the original data,

$$W_{b,i}(\lambda )=W_{i}+\sqrt{\lambda }U_{b,i},\;b=1,\ldots ,B,\;i=1,\ldots ,n,$$

where $U_{b,i}$ is a vector of mutually independent pseudo‐errors, independent of $X_{i}$, and generated from a Normal distribution with zero mean and variance/covariance matrix ${\hat{\Gamma }}_{i}$. The new mismeasured variable $W_{b,i}(\lambda )$ has expected value equal to $X_{i}$ and variance/covariance matrix equal to $(1+\lambda ){\hat{\Gamma }}_{i}$. Its mean squared error $\text{MSE}\{W_{b,i}(\lambda )\}=E\{{\left(W_{b,i}(\lambda )-X_{i}\right)}^{2}|X_{i}\}$ equals zero when λ=−1. Once the additional mismeasured data are available, the estimate ${\hat{\psi }}_{b}(\lambda )$ of ψ for given *b* and λ is obtained, by applying the uncorrected or naive model, for example, the weighted least squares regression,^4^ to data $W_{b,i},i=1,\ldots ,n$. The simulation step ends with the average of the estimates over *b* for a fixed λ,

$$\hat{\psi }(\lambda )=B^{-1}\overset{B}{\underset{b=1}{\sum }}\;{\hat{\psi }}_{b}(\lambda ).$$

The *extrapolation step* determines a relationship between $\hat{\psi }(\lambda )$ and λ, one parameter at a time. The relationship is extrapolated back to the case of no measurement error, that is, to λ=−1. The resulting estimate is the SIMEX estimate ${\hat{\psi }}_{\text{SIMEX}}$.

The variance/covariance matrix associated to ${\hat{\psi }}_{\text{SIMEX}}$ can be easily calculated when the measurement error variance/covariance matrix is known or estimated well enough, see Stefanski and Cook^31^ and Carroll et al.^11^ ^(AppendixB.4)^ Let ${ŝ}_{b}^{2}(\lambda )$ denote the estimated model‐based variance/covariance matrix of ${\hat{\psi }}_{b}(\lambda )$ obtained from the naive model and let ${ŝ}^{2}(\lambda )$ be the average of ${ŝ}_{b}^{2}(\lambda )$ over *b*,

$${ŝ}^{2}(\lambda )=B^{-1}\overset{B}{\underset{b=1}{\sum }}\;{ŝ}_{b}^{2}(\lambda ).$$

Let $s_{\Delta }^{2}(\lambda )$ denote the sample variance/covariance matrix of terms ${\hat{\psi }}_{b}(\lambda )$, b=1,…,B. The variance/covariance matrix of ${\hat{\psi }}_{\text{SIMEX}}$ is obtained by extrapolating back the relationship between the components of the difference ${ŝ}^{2}(\lambda )-s_{\Delta }^{2}(\lambda )$ and λ to the case λ=−1, one parameter at a time.

## 4 SIMULATION STUDY

Several simulation studies have been conducted to investigate the performance of the structural and functional methods in terms of accuracy of inferential results. The likelihood approach under the Normal and the Skew‐Normal specification for the control risk distribution, corrected score, conditional score, and SIMEX are compared to the uncorrected weighted least squares regression,^4^ also referred to as the naive approach. Simulations are implemented in the R programming language.^32^

### 4.1 Set‐up

Data simulation follows a two‐stage procedure. In the first stage, values for $\xi_{i}$ are generated according to one of the following choices:

1. A standard Normal distribution;
2. A Skew‐Normal distribution SN(0,1,1), in order to investigate the performance of the methods under skewness of the risk distribution;
3. A mixture of Normal distributions, in order to investigate the performance of the methods under bimodality of the risk distribution, namely,
$$\pi N\left(-(1-\pi )\mu ,\sigma^{2}\right)+(1-\pi )N\left(\pi \mu ,\sigma^{2}\right),$$
  where $\pi =0.25,\mu =1.5,\sigma^{2}=0.05$.

Values of $\eta_{i}$ are generated following relationship (1) with ${\left(\beta_{0},\beta_{1}\right)}^{⊤}={\left(0,1\right)}^{⊤}$. The parameter choice reflects the case of no relationship between the treatment benefit $\eta_{i}-\xi_{i}$ and the underlying risk $\xi_{i}$, for example, Ghidey et al.^14^ In the second stage, values of ${\hat{\eta }}_{i}$ and ${\hat{\xi }}_{i}$ are obtained conditionally on ${\left(\eta_{i},\xi_{i}\right)}^{⊤}$ for each study, by distinguishing two scenarios:

(i) $\eta_{i}$ and $\xi_{i}$ are the log‐odds in the treatment group and in the control group for study *i*, respectively; their observed versions are measured as
(5) $${\hat{\eta }}_{i}=\log\left(\frac{y_{i}}{n_{T_{i}}-y_{i}}\right),\;{\hat{\xi }}_{i}=\log\left(\frac{x_{i}}{n_{C_{i}}-x_{i}}\right),$$
  where $y_{i}$ and $x_{i}$ are the observed number of events in the treatment group and in the control group for study *i*, respectively, and $n_{T_{i}}$ and $n_{C_{i}}$ are the number of treated and controls, respectively; thus, $y_{i}$ and $x_{i}$ are generated as follows,^15^
$$Y_{i}∼\text{Binomial}\left(n_{T_{i}},\frac{e^{\eta_{i}}}{1+e^{\eta_{i}}}\right),\;X_{i}∼\text{Binomial}\left(n_{C_{i}},\frac{e^{\xi_{i}}}{1+e^{\xi_{i}}}\right);$$
  the numbers of treated and controls are generated from a Uniform distribution on [15,200]. For structural approaches, matrix ${\hat{\Gamma }}_{i}$ is obtained as
(6) $${\hat{\Gamma }}_{i}=\left[\begin{array}{cc} y_{i}^{-1}+{\left(n_{T_{i}}-y_{i}\right)}^{-1} & 0\\ 0 & x_{i}^{-1}+{\left(n_{C_{i}}-x_{i}\right)}^{-1} \end{array}\right].$$
  The correlation between ${\hat{\eta }}_{i}$ and ${\hat{\xi }}_{i}$ is zero as the estimates of $\eta_{i}$ and $\xi_{i}$ are computed on different subjects, treated and controls, respectively;
(ii) $\eta_{i}$ and $\xi_{i}$ are the log event rate in the treatment group and in the control group for study *i*, respectively; their observed versions are measured as
$${\hat{\eta }}_{i}=\log\left(\frac{y_{i}}{n_{y_{i}}}\right)\;\text{and}\;{\hat{\xi }}_{i}=\log\left(\frac{x_{i}}{n_{x_{i}}}\right),$$
  where $y_{i}$ and $x_{i}$ are the observed number of events in the treatment group and in the control group for study *i*, respectively, and $n_{y_{i}}$ and $n_{x_{i}}$ are the number of person‐years in the treatment group and in the control group, respectively; thus, $y_{i}$ and $x_{i}$ are generated as follows,^15^
$$Y_{i}∼\text{Poisson}\left(n_{y_{i}}e^{\eta_{i}}\right)\;\text{and}\;X_{i}∼\text{Poisson}\left(n_{x_{i}}e^{\xi_{i}}\right);$$
  the numbers of person‐years in the treatment group and in the control group are generated from a Uniform distribution on [100,5000]. For structural approaches, matrix ${\hat{\Gamma }}_{i}$ is still obtained as a diagonal matrix,
$${\hat{\Gamma }}_{i}=\left[\begin{array}{cc} y_{i}^{-1} & 0\\ 0 & x_{i}^{-1} \end{array}\right].$$

For each scenario, increasing values of the variance component are considered, $\tau^{2}\in \{0.1,0.5,0.9,1.5\}$, as well as increasing values of the number of studies included in the meta‐analysis, n∈{10,20,50}. One thousand datasets are generated for each scenario and each combination of sample size *n* and variance $\sigma^{2}$. Likelihood maximization, based on the Nelder and Mead algorithm,^33^ employs the weighted least squares estimates as starting values. Numerical integration needed for likelihood maximization in case of Skew‐Normal specification of the distribution of $\xi_{i}$ is performed through a Gauss‐Hermite quadrature.

The application of SIMEX considers λ assuming values on Λ={0.0,0.5,1.0,1.5,2.0}, following the conventional choice in the literature. Similarly, the number of remeasured datasets *B* is fixed at 200. The quadratic extrapolation function is chosen to model the relationship between $\hat{\psi }(\lambda )$ and λ, given its numerical stability with respect to alternatives, see Carroll et al.^11^ ^(Section5.3.2)^

### 4.2 Results

**FIGURE 1:**
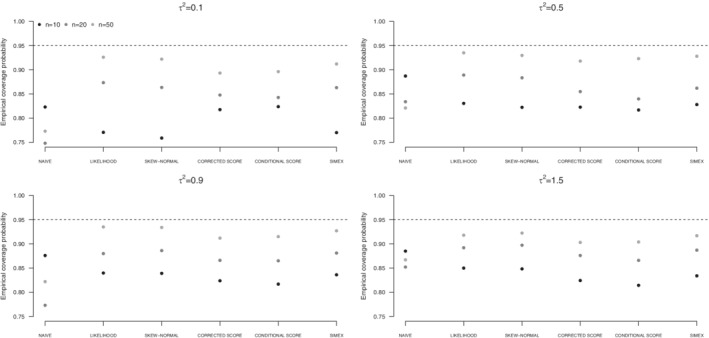
Empirical coverage probabilities of confidence intervals for $\beta_{1}$ from uncorrected approach (NAIVE), likelihood approach under a Normal specification (LIKELIHOOD) or a Skew‐Normal specification (SKEW‐NORMAL) for the underlying risk distribution, SIMEX, corrected score, and conditional score, on the basis of 1000 replicates of simulation scenario (i). Underlying risk normally distributed

**FIGURE 2:**
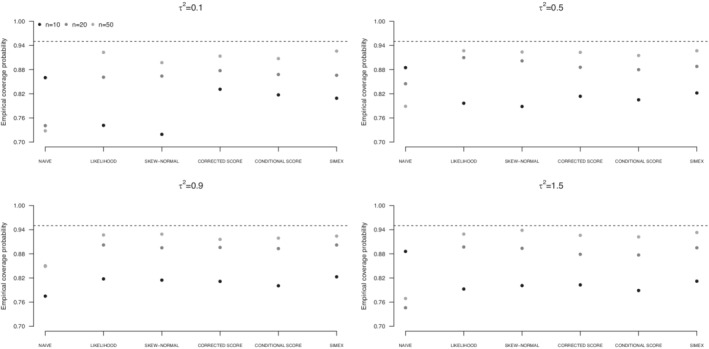
Empirical coverage probabilities of confidence intervals for $\beta_{1}$ from uncorrected approach (NAIVE), likelihood approach under a Normal specification (LIKELIHOOD) or a Skew‐Normal specification (SKEW‐NORMAL) for the underlying risk distribution, SIMEX, corrected score, and conditional score, on the basis of 1000 replicates of simulation scenario (i). Underlying risk distributed as a mixture of Normals

**FIGURE 3:**
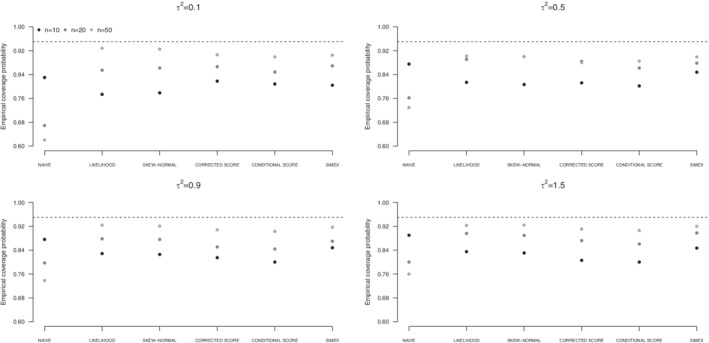
Empirical coverage probabilities of confidence intervals for $\beta_{1}$ from uncorrected approach (NAIVE), likelihood approach under a Normal specification (LIKELIHOOD) or a Skew‐Normal specification (SKEW‐NORMAL) for the underlying risk distribution, SIMEX, corrected score and conditional score, on the basis of 1000 replicates of simulation scenario (i). Underlying risk distributed as a Skew‐Normal

Results for scenario (i) are graphically reported in terms of empirical coverage probabilities of confidence intervals at nominal level 0.95 for $\beta_{1}$ (Figures 1, 2, 3) and numerically reported in terms of bias, standard deviation of the estimates, and estimated standard error of the estimator of the variance component $\tau^{2}$ (Tables 1, 2, 3). Analogous results for the estimators of $\beta_{0}$ and $\beta_{1}$ and empirical coverage probabilities of confidence intervals at nominal level 0.95 for $\beta_{0}$ are reported in the Supplementary Material. The Supplementary Material contains also the values of the empirical coverage probabilities for $\beta_{0}$ and $\beta_{1}$ and the associated Monte Carlo standard error (eg, Morris et al.^34^). Analogous results for scenario (ii) are reported in the Supplementary Material.

**TABLE 1:** Bias and standard deviation (SD) of the estimates of $\tau^{2}$, and average of the estimated standard errors (SE) obtained from uncorrected approach (NAIVE), likelihood analysis under a Normal or a Skew‐Normal specification of the distribution of ξ, corrected score, conditional score, SIMEX, on the basis of 1000 replicates of simulation scenario (i)

$\tau^{2}$	Method	Bias	SD	SE	Mean	SD	SE	Mean	SD	SE
0.1		n=10			n=20			n=50		
	NAIVE	0.066	0.091	0.083	0.163	0.102	0.088	0.133	0.057	0.048
	LIKELIHOOD	−0.037	0.068	0.031	−0.029	0.067	0.034	−0.009	0.040	0.023
	SKEW‐NORMAL	−0.037	0.069	0.031	−0.029	0.068	0.034	−0.009	0.040	0.023
	CORRECTED SCORE	−0.014	0.067	0.144	0.007	0.069	0.136	−0.008	0.042	0.071
	CONDITIONAL SCORE	−0.014	0.067	0.143	0.007	0.069	0.139	−0.008	0.043	0.070
	SIMEX	−0.001	0.111	0.146	0.014	0.105	0.117	0.010	0.056	0.056
0.5		n=10			n=20			n=50		
	NAIVE	0.011	0.274	0.255	0.177	0.257	0.226	0.171	0.156	0.137
	LIKELIHOOD	−0.139	0.239	0.238	−0.090	0.210	0.236	−0.033	0.129	0.166
	SKEW‐NORMAL	−0.139	0.241	0.243	−0.090	0.210	0.239	−0.033	0.129	0.167
	CORRECTED SCORE	−0.129	0.241	0.155	−0.079	0.214	0.141	−0.032	0.132	0.087
	CONDITIONAL SCORE	−0.129	0.241	0.155	−0.079	0.214	0.140	−0.032	0.132	0.087
	SIMEX	0.015	0.331	0.381	0.044	0.265	0.270	0.036	0.150	0.147
0.9		n=10			n=20			n=50		
	NAIVE	−0.028	0.456	0.436	0.226	0.440	0.375	0.182	0.240	0.221
	LIKELIHOOD	−0.261	0.372	0.496	−0.151	0.306	0.484	−0.087	0.201	0.347
	SKEW‐NORMAL	−0.256	0.375	0.508	−0.152	0.307	0.488	−0.086	0.201	0.349
	CORRECTED SCORE	−0.229	0.394	0.182	−0.148	0.317	0.155	−0.086	0.204	0.105
	CONDITIONAL SCORE	−0.228	0.393	0.180	−0.148	0.317	0.154	−0.086	0.204	0.104
	SIMEX	0.019	0.536	0.599	0.034	0.380	0.386	0.040	0.234	0.234
1.5		n=10			n=20			n=50		
	NAIVE	−0.077	0.753	0.711	0.210	0.612	0.570	0.213	0.363	0.350
	LIKELIHOOD	−0.549	0.440	0.836	−0.317	0.430	0.924	−0.109	0.326	0.757
	SKEW‐NORMAL	−0.547	0.450	0.841	−0.317	0.433	0.930	−0.105	0.328	0.761
	CORRECTED SCORE	−0.344	0.657	0.231	−0.216	0.534	0.198	−0.106	0.327	0.133
	CONDITIONAL SCORE	−0.344	0.657	0.229	−0.216	0.535	0.196	−0.106	0.327	0.133
	SIMEX	0.085	0.892	0.987	0.097	0.631	0.643	0.091	0.362	0.373

*Note*: Underlying risk normally distributed.

**TABLE 2:** Bias and standard deviation (SD) of the estimates of $\tau^{2}$, and average of the estimated standard errors (SE) obtained from uncorrected approach (NAIVE), likelihood analysis under a Normal or a Skew‐Normal specification of the distribution of ξ, corrected score, conditional score, SIMEX, on the basis of 1000 replicates of simulation scenario (i)

$\tau^{2}$	Method	Bias	SD	SE	Mean	SD	SE	Mean	SD	SE
0.1		n=10			n=20			n=50		
	NAIVE	0.056	0.078	0.078	0.096	0.070	0.065	0.116	0.050	0.044
	LIKELIHOOD	−0.031	0.067	0.032	−0.021	0.058	0.028	−0.010	0.038	0.022
	SKEW‐NORMAL	−0.032	0.067	0.032	−0.022	0.058	0.031	−0.009	0.037	0.022
	CORRECTED SCORE	−0.012	0.064	0.137	−0.013	0.057	0.107	−0.012	0.041	0.073
	CONDITIONAL SCORE	−0.012	0.064	0.134	−0.013	0.058	0.106	−0.012	0.041	0.072
	SIMEX	0.006	0.099	0.130	0.009	0.079	0.084	0.009	0.049	0.050
0.5		n=10			n=20			n=50		
	NAIVE	−0.001	0.260	0.249	0.087	0.202	0.196	0.149	0.150	0.132
	LIKELIHOOD	−0.124	0.241	0.252	−0.083	0.174	0.209	−0.047	0.119	0.155
	SKEW‐NORMAL	−0.120	0.244	0.257	−0.079	0.175	0.214	−0.040	0.122	0.159
	CORRECTED SCORE	−0.116	0.235	0.154	−0.087	0.176	0.123	−0.046	0.120	0.084
	CONDITIONAL SCORE	−0.116	0.235	0.152	−0.086	0.176	0.122	−0.045	0.120	0.084
	SIMEX	0.023	0.320	0.367	0.015	0.215	0.226	0.030	0.142	0.141
0.9		n=10			n=20			n=50		
	NAIVE	0.119	0.628	0.510	0.100	0.361	0.333	0.159	0.238	0.216
	LIKELIHOOD	−0.276	0.362	0.489	−0.146	0.292	0.482	−0.078	0.206	0.355
	SKEW‐NORMAL	−0.267	0.374	0.500	−0.136	0.304	0.499	−0.075	0.210	0.356
	CORRECTED SCORE	−0.244	0.397	0.183	−0.142	0.301	0.153	−0.077	0.208	0.105
	CONDITIONAL SCORE	−0.243	0.397	0.180	−0.141	0.302	0.152	−0.077	0.208	0.105
	SIMEX	0.000	0.543	0.585	0.045	0.374	0.384	0.053	0.237	0.234
1.5		n=10			n=20			n=50		
	NAIVE	−0.130	0.699	0.685	0.390	0.795	0.630	0.267	0.408	0.361
	LIKELIHOOD	−0.556	0.457	0.826	−0.304	0.398	0.892	−0.136	0.327	0.727
	SKEW‐NORMAL	−0.546	0.458	0.832	−0.305	0.394	0.892	−0.133	0.319	0.727
	CORRECTED SCORE	−0.384	0.596	0.227	−0.233	0.480	0.189	−0.133	0.328	0.130
	CONDITIONAL SCORE	−0.384	0.596	0.223	−0.233	0.480	0.187	−0.133	0.328	0.129
	SIMEX	0.034	0.828	0.948	0.072	0.590	0.611	0.085	0.372	0.367

*Note*: Underlying risk distributed as a mixture of Normals.

**TABLE 3:** Bias and standard deviation (SD) of the estimates of $\tau^{2}$, and average of the estimated standard errors (SE) obtained from uncorrected approach (NAIVE), likelihood analysis under a Normal or a Skew‐Normal specification of the distribution of ξ, corrected score, conditional score, SIMEX, on the basis of 1000 replicates of simulation scenario (i)

$\tau^{2}$	Method	Bias	SD	SE	Mean	SD	SE	Mean	SD	SE
0.1		n=10			n=20			n=50		
	NAIVE	0.073	0.089	0.087	0.238	0.169	0.113	0.171	0.077	0.055
	LIKELIHOOD	−0.032	0.069	0.033	−0.019	0.080	0.038	−0.007	0.044	0.025
	SKEW‐NORMAL	−0.032	0.070	0.033	−0.016	0.082	0.044	−0.007	0.044	0.025
	CORRECTED SCORE	−0.008	0.068	0.145	0.016	0.088	0.143	−0.006	0.050	0.084
	CONDITIONAL SCORE	−0.008	0.068	0.143	0.017	0.089	0.137	−0.006	0.050	0.082
	SIMEX	0.011	0.108	0.149	0.020	0.125	0.128	0.016	0.064	0.062
0.5		n=10			n=20			n=50		
	NAIVE	0.029	0.274	0.264	0.207	0.262	0.236	0.218	0.178	0.147
	LIKELIHOOD	−0.130	0.239	0.255	−0.094	0.205	0.236	−0.038	0.127	0.167
	SKEW‐NORMAL	−0.134	0.239	0.254	−0.093	0.211	0.239	−0.039	0.127	0.167
	CORRECTED SCORE	−0.121	0.234	0.161	−0.085	0.207	0.145	−0.037	0.132	0.091
	CONDITIONAL SCORE	−0.121	0.234	0.159	−0.084	0.208	0.142	−0.037	0.132	0.090
	SIMEX	0.028	0.328	0.394	0.039	0.259	0.278	0.040	0.151	0.154
0.9		n=10			n=20			n=50		
	NAIVE	−0.004	0.460	0.448	0.271	0.447	0.390	0.260	0.268	0.237
	LIKELIHOOD	−0.264	0.362	0.511	−0.146	0.316	0.519	−0.060	0.213	0.376
	SKEW‐NORMAL	−0.261	0.369	0.520	−0.152	0.312	0.514	−0.060	0.212	0.376
	CORRECTED SCORE	−0.238	0.388	0.189	−0.135	0.342	0.168	−0.059	0.216	0.110
	CONDITIONAL SCORE	−0.237	0.387	0.186	−0.134	0.342	0.165	−0.059	0.217	0.109
	SIMEX	0.029	0.535	0.624	0.069	0.410	0.428	0.072	0.248	0.247
1.5		n=10			n=20			n=50		
	NAIVE	−0.131	0.714	0.685	0.409	0.709	0.636	0.298	0.413	0.367
	LIKELIHOOD	−0.577	0.443	0.806	−0.291	0.438	0.936	−0.127	0.333	0.737
	SKEW‐NORMAL	−0.575	0.438	0.809	−0.290	0.441	0.942	−0.130	0.334	0.736
	CORRECTED SCORE	−0.381	0.634	0.229	−0.176	0.541	0.200	−0.126	0.338	0.132
	CONDITIONAL SCORE	−0.380	0.635	0.225	−0.174	0.541	0.196	−0.126	0.338	0.131
	SIMEX	0.042	0.863	0.963	0.144	0.645	0.656	0.075	0.376	0.370

*Note*: Underlying risk distributed as a Skew‐Normal.

As expected, the weighted least squares approach provides unsatisfactory results, with empirical coverage probabilities for $\beta_{1}$ below the 95% target level. Such a behavior appears whichever the distribution of the underlying risk, and it does not ameliorate increasing the sample size. This is a consequence of the downward bias of $\beta_{1}$ (Tables S5‐S7 in the Supplementary Material), which turns out in confidence intervals centered on a value far from the target level, with standard error that reduces as the sample size increases. The unsatisfactory performance is more evident in case of small between‐study variance $\tau^{2}$. A similar behavior affects inference on $\beta_{0}$, see the corresponding results in the Supplementary Material (Tables S1‐S3 and Figures S1‐S3).

Likelihood solutions, using a Normal specification or a Skew‐Normal specification of the underlying risk distribution, provide almost unbiased estimators of the regression parameters $\beta_{0}$ and $\beta_{1}$ under all the examined situations, as expected from a theoretical point of view. See the corresponding results in the Supplementary Material (Tables S1‐S3 and Tables S5‐S7). Conversely, the estimators of the between‐study variance $\tau^{2}$ are affected and tend to underestimate the true value. The bias remarkably increases with $\tau^{2}$, see Tables 1, 2, 3. Increasing the sample size is helpful to reduce the bias as well as the standard errors of the estimators. The discrepancy between the standard deviation of the estimators of $\tau^{2}$ and the corresponding average standard errors is a consequence of the assumptions on the distribution of $\xi_{i}$ made in the structural approaches. Mean squared error associated to likelihood‐based solutions increases with $\tau^{2}$, and it appears to be substantial with reference to the estimator of $\tau^{2}$, as a consequence of the large bias. See the results in the Supplementary Material, namely, Figures S1 to S3 with reference to the estimators of $\beta_{0}$, Figures S7 to S9 with reference to the estimators of $\beta_{1}$, and Figures S10 to S12 with reference to the estimators of $\tau^{2}$. Such a behavior emerges under a Normal distribution for the underlying risk and is even more evident under departures from normality. As expected, the mean squared error largely reduces increasing the sample size. The biased estimates of $\tau^{2}$ affect the empirical coverage probabilities for the regression coefficients, that are far from the target level, especially for small *n*. Improvements are obtained with increasing sample size. With n=50 the empirical coverage probabilities are very close to the target level. See Figures 1, 2, 3 and Table S8 with reference to $\beta_{1}$, and Figures S4 to S6 and Table S4 with reference to $\beta_{0}$. A slightly better performance in terms of empirical coverage probabilities is obtained when a Skew‐Normal specification for the distribution of $\xi_{i}$ is adopted in place of the Normal specification, for large $\tau^{2}$ and $\xi_{i}$ not normally distributed (Figures 2 and 3).

Score functions reveal helpful to improve on likelihood‐based approaches in terms of empirical coverage probabilities of confidence intervals for $\beta_{1}$ in case of small sample size and small values of the between‐study variance $\tau^{2}$. See, for example, the results when the underlying risk is distributed as a Normal (Figure 1) or as a mixture of Normals (Figure 2). The result is even more evident with reference to $\beta_{0}$, see the corresponding empirical coverage probabilities in Figures S4 to S6 and in Table S4 in the Supplementary Material. Conversely, likelihood solutions tend to perform similarly or even better, in case of large $\tau^{2}$, see Tables 1, 2, 3. With respect to the estimation of $\tau^{2}$, both the corrected score and the conditional score are preferable to the likelihood‐based solutions, as they provide estimators with a reduced bias and a reduced standard error, especially for large $\tau^{2}$, whichever the underlying risk distribution. This result translates into a much smaller mean squared error, whichever the underlying risk distribution (Figures S10‐S12 in the Supplementary Material). Such a behavior is in line with previous findings in Ghidey et al.^14^ The score functions approaches tend to perform similarly, the only difference being a slightly reduced standard error of the estimators of the parameters $\beta_{0}$ and $\beta_{1}$ from the conditional score function with respect to the corrected score function, see Tables S1 to S3 and Tables S5 to S7 in the Supplementary Material. The difference is more evident in case of small sample size and in case of deviations from the normality for the underlying risk distribution. The estimators of $\tau^{2}$ perform similarly, see Tables 1, 2, 3. No relevant differences are experienced in terms of empirical coverage probabilities for the estimators of the regression coefficients (Figures 1‐3 and Table S8).

Results from SIMEX are largely satisfactory. Bias of the estimators of $\beta_{0}$ and $\beta_{1}$ is small whichever the examined scenario and it is not affected by the sample size or the increasing between‐study variance $\tau^{2}$, see Tables S1 to S3 and Tables S5 to S7 in the Supplementary Material. SIMEX estimator of $\beta_{1}$ is less biased than the estimator provided by likelihood‐based solutions, especially in case of small sample size n=10. Similarly, the SIMEX estimator of $\tau^{2}$ has a small bias, outperforming alternative approaches for small *n*, with substantial improvement over likelihood‐based solutions (Tables 1, 2, 3). The price to pay is a larger standard error, which reflects the simulation‐based nature of the correction method. Increasing the sample size reduces the standard error, sometimes markedly, as it happens for large $\tau^{2}$, if compared to likelihood‐based solutions. Globally, the standard deviations of the estimators of $\tau^{2}$ are consistent with the estimated standard errors. The mean square error of the estimators is smaller than that from the likelihood‐based approaches, especially when the inferential interest is on $\tau^{2}$ (Figures S10‐S12 in the Supplementary Material), and slightly larger with respect to the score approaches. Empirical coverage probabilities of confidence intervals for $\beta_{1}$ tend to be closer to the target level than competing approaches, with emphasis in case of small sample size and moderate to large between‐study variance. Advantages are evident in case of skewness of the underlying risk distribution, see Figure 3 and Table S4.

An additional simulation has been carried out to compare the methods in case of large sample size, equal to 100. The examined scenario refers to the Normal distribution for the underlying risk. Results are reported in the Supplementary Material (Table S9 and Figures S13‐S17). Under such a large sample size, the behavior of the competing methods is comparable (Table S9), and superior to that of the naive analysis, in line with the findings in Ghidey et al^14^ referred to the score functions and the likelihood‐based solution. Efficiency in terms of mean squared error is similar when inference is on the regression parameters $\beta_{0}$ and $\beta_{1}$ (Figures S13 and S14). A similar result holds when interest is on the between‐study variance $\tau^{2}$, although likelihood‐based estimators tend to have larger mean squared error for large $\tau^{2}$ (Figure S15). Nevertheless, the mean squared error reaches small values if compared to scenarios with reduced sample size, as expected. Empirical coverage probabilities of confidence intervals for $\beta_{0}$ and $\beta_{1}$ are closer to the target 95% level under all the approaches, providing an evident amelioration over the naive analysis (Figures S16 and S17).

A comparison of the correction techniques from a computational point of view highlights convergence problems of likelihood approaches, which typically occur as nonpositive definite variance/covariance matrix. The issue is frequent in case of small sample size and/or small between‐study variance. The failure rate reaches about 15% for n=10 and $\tau^{2}=0.1$. The application of the corrected score and the conditional score experiences some convergence issues as well. The corrected score has a slightly larger failure rate if compared to the conditional score, reaching 15% to 16% for n=10 and $\tau^{2}=0.1$. SIMEX is the correction method less affected by computational problems, under all the examined scenarios. A nonpositive definite SIMEX estimated variance/covariance matrix is a possible issue related to SIMEX application, although not frequent Carroll et al.^11^ ^(SectionB.4.1)^ It has been not experienced in the performed simulation studies.

## 5 EXAMPLE

Lu et al^35^ consider a meta‐analysis of 14 case‐control studies about the association between prior onset of diabetes and the risk of Parkinson disease. Information is available about the number of events and the number of subjects in the diabetes group and in the control group, as reported in Table 4. The meta‐analysis in Lu et al^35^ has the aim of providing an additional investigation of the relationship between the two pathologies, a topic that has received substantial attention as the results in the literature are often inconsistent, giving rise to positive association, null association or inverse association. The analysis in Lu et al^35^ performed through a random‐effects approach of the log‐odds ratio from each study suggests a negative association between the two pathologies (summary odds ratio = 0.75, 95% confidence interval 0.58‐0.98). A limitation of the study highlighted by the authors is the presence of several sources of heterogeneity to be controlled for, as heterogeneity is due to gender, geographical region, source of the control groups, and severity of diabetes mellitus. In this light, the use of control risk regression can be advantageous. Let $\eta_{i}$ and $\xi_{i}$ denote the log‐odds in the *i*th case group and control group, respectively, and let their observed error‐prone versions ${\hat{\eta }}_{i}$ and ${\hat{\xi }}_{i}$ evaluated as in (5), that is, ${\hat{\eta }}_{i}=\log\left\{y_{i}/(n_{y_{i}}-y_{i})\right\}$, ${\hat{\xi }}_{i}=\log\left\{x_{i}/(n_{x_{i}}-x_{i})\right\}$. The within‐study variance/covariance matrix ${\hat{\Gamma }}_{i}$ is estimated as in (6). The inferential interest is on $\beta_{1}$, with a value smaller than one indicating that the increase of the log‐odds in the control condition by a certain amount leads to a reduced increase of risk for the case group. The results after applying the likelihood approach under a Normal specification of the risk distribution, SIMEX, the corrected score and the conditional score for ${\left(\beta_{0},\beta_{1},\tau^{2}\right)}^{⊤}$ are reported in Table 5, with standard errors for the estimators of the regression parameters in parentheses. The results from the uncorrected approach are reported as well. The use of a Skew‐Normal specification of the control risk distribution in the likelihood approach does not give benefits, as the skewness parameter is estimated equal to zero. The corresponding results are not displayed, as the method reduces to the likelihood approach under a Normal control risk distribution.

**TABLE 4:** Parkinson disease data

	Cases of Parkinson disease		Controls	
Study	Events	Total	Events	Total
1	6	35	12	105
2	6	178	58	534
3	18	212	8	175
4	12	74	18	148
5	11	93	26	93
6	13	196	17	196
7	10	249	39	368
8	13	318	31	318
9	18	197	24	197
10	17	228	29	228
11	26	352	61	484
12	48	13 695	223	68 445
13	126	1931	482	9651
14	291	3637	308	3637

*Note*: Number of events and totals in the diabetes group and in the control group in the meta‐analysis of Lu et al.^35^

**TABLE 5:** Parkinson disease data

	$\beta_{0}$	$\beta_{1}$	$\tau^{2}$
NAIVE	−0.459 (0.283)	0.853 (0.098)	0.349 (0.241)
LIKELIHOOD NORMAL	−0.626 (0.254)	0.886 (0.086)	0.115 (0.031)
CORRECTED SCORE	−0.766 (0.331)	0.803 (0.085)	0.147 (0.078)
CONDITIONAL SCORE	0.748 (0.321)	0.810 (0.074)	0.147 (0.079)
SIMEX	−0.847 (0.321)	0.799 (0.108)	0.245 (0.101)

*Note*: Estimates and estimated standard errors in parentheses for the parameters in model (1), obtained from uncorrected approach (NAIVE), likelihood analysis under a Normal specification for the control rate distribution, corrected score, conditional score, SIMEX.

All the approaches provide estimates of the regression coefficient $\beta_{1}$ smaller than one, suggesting that diabetes affected subjects are associated with a lower risk of Parkinson disease than patients in the control condition. In this way, the conclusions in Reference 35 are supported, although the correction techniques result in a less strong association between diabetes and risk of Parkinson disease than the weighted least squares approach. The estimate of the intercept is substantially lower if the measurement error is not taken into account; more interestingly, not accounting for the presence of measurement error produces a much larger estimate of the heterogeneity component $\tau^{2}$, if compared to correction approaches, with a substantially larger standard error. The finding reflects the capability of the correction techniques to capture the variability among studies due to the presence of errors affecting the measures of $\eta_{i}$ and $\xi_{i}$. Not accounting for such component, in the naive analysis, gives rise to a larger estimate of the between‐study heterogeneity parameter $\tau^{2}$. As experienced in the simulation studies, there is no relevant difference between the corrected score and the conditional score. The normality assumption of the structural approach is difficult to be tested given the small number of studies included in the meta‐analysis (eg, Ghidey et al^14^). Nevertheless, the results from the approach are in line with those from competing methods and the use of a skewed distribution reveals to be not necessary. From a practical point of view, no computational problems arise when applying the correction techniques.

## 6 CONCLUDING REMARKS

This article focused on inference in control risk regression, where the presence of measurement error affects the outcome risk measure of both the treatment group and the control group. Inference ignoring the presence of measurement error typically gives rise to unreliable inferential conclusions. In this article, different correction techniques from either a structural or a functional approach for dealing with measurement errors have been compared through simulation. Attention has been paid to likelihood‐based solutions under a Normal or a Skew‐Normal distribution for the underlying risk distribution, score functions, and the simulation‐extrapolation SIMEX method. The approaches have been compared under different scenarios, including small to large sample size, increasing between‐study heterogeneity, underlying risk normally or not‐normally distributed in the control condition. The paper represents an extension of a previous comparison between correction methods developed in Ghidey et al,^14^ with new contributions given by the inclusion of additional approaches, different scenarios, further criteria for the evaluation of the methods. The adopted data generation process has been designed in order to reflect the control risk generation mechanism.

Simulation results indicate that correcting for measurement error is a necessary step, as the naive weighted least squares approach ignoring the errors provides largely unsatisfactory inferential results. Among the correction techniques, likelihood‐based solutions suffer for small‐sample bias of the estimator of the heterogeneity parameter, with emphasis for large values of the heterogeneity component. The result affects the empirical coverage of confidence interval for the estimators of the regression coefficients, which tends to be lower than the target level. The unsatisfactory performance of the likelihood approach under a Normal specification of the underlying risk distribution is partially improved using the Skew‐Normal specification. The use of corrected score function or conditional score function reveals helpful in improving on likelihood approaches in terms of empirical coverage of confidence intervals for small sample size and small values of the heterogeneity component. For large heterogeneity, likelihood solutions remain preferable. No substantial differences emerge between the two score functions, the conditional score function providing a slightly reduced standard error of the estimators of the regression coefficients. SIMEX reveals satisfactory in terms of bias of the estimators of the regression coefficients and the heterogeneity components, whichever the examined scenario and the sample size, although at the price of a larger standard error. Empirical coverage probabilities for the estimator of the regression coefficients are closer to the target level than competing approaches, especially in case of skewness of the underlying risk distribution. In addition, it has the advantage of not suffering from convergence problems. In this sense, it is preferable to likelihood‐based solutions and score functions, whose application is limited by substantial failure rate in convergence, especially for small sample size or large heterogeneity.

All the approaches allow to quickly carry out control risk regression, as they take only few seconds in case of large sample size of the examined meta‐analysis. Computational time required by SIMEX could increase if the number *B* of replicates in the simulation step is fixed to a larger value than examined. However, some investigations with larger *B* show that the performance of SIMEX does not substantially vary.

The application of the methods has been illustrated in a real meta‐analysis about the association of diabetes and risk of Parkinson disease. Correcting for measurement error in control risk regression suggests a decreased risk of Parkinson disease for diabetic patients if compared to controls, with a substantial larger estimate of the variance component from the correction techniques with respect to the naive approach which ignores the presence of measurement errors.

The paper did not consider the moment‐based correction method (Buonaccorsi^12^ ^(Section5)^ and Ghidey et al^14^). Previous results in Guolo^18^ show that the method shares with SIMEX the lack of any assumption about the underlying baseline risk distribution, however at the price of a less accurate evaluation of the uncertainty of the estimators. In addition, the moment based correction may provide inadmissible estimates of some components, such as, for example, negative estimates of variance quantities, a situation which requires ad hoc corrections, see, for example, Buonaccorsi.^12^ ^(Section5.4.4)^

At the time of writing, the code for implementing some of the approaches developed for measurement error correction in control risk regression is made available from the authors. We refer to the code in the R ^32^ programming language used for implementing the score functions in Ghidey et al^14^ and SIMEX in Guolo.^17^

The performed study has the first aim of warning researchers against the risk due to not accounting for measurement error in control risk regression and secondly of helping in choosing an appropriate correction technique to be applied to the available meta‐analysis. Control risk regression is a powerful instrument to explain a portion of the heterogeneity in meta‐analysis studies: We hope this article motivates researchers to make an appropriate use of it through the inclusion of measurement error correction techniques to prevent fallacious results.

## Supporting information


**Data S1.** Supplementary material

## Data Availability

Data sharing is not applicable to this article as no new data were created or analyzed in this study.
