# Supplementary material for: Measurement errors in control risk regression: A comparison of correction techniques
Source: Stat Med. 2021 Oct 15;41(1):163–79. doi: 10.1002/sim.9228 (PMC9292416; doi:10.1002/sim.9228)
Supplement: Supplementary file 1 — Data S1. Supplementary material [file SIM-41-163-s001.pdf]

Web-based Supporting Materials for "Measurement errors in control risk regression: a comparison of correction techniques" by Annamaria Guolo

Web Appendix A: Additional simulation results for the log odds case

This web appendix reports a portion of the results of the simulation study carried out to compare the performance of the competing approaches in control risk regression, as described in Section 4 of the main manuscript. The reference is to simulation scenario *i*) in the main manuscript.

Table S1: Bias and standard deviation (SD) of the estimates of β_0, and average of the estimated standard errors (SE) obtained from naive analysis, likelihood analysis under a Normal or a Skew-Normal specification of the distribution of ξ, corrected score, conditional score, SIMEX, on the basis of $1,000$ replicates of simulation scenario i). Underlying risk normally distributed.

τ^2	Method	Bias	SD	SE	Bias	SD	SE	Bias	SD	SE
0.1			$n = 10$			$n = 20$			$n = 50$	
	NAIVE	-0.002	0.157	0.083	0.003	0.137	0.088	0.001	0.076	0.048
	LIKELIHOOD	-0.001	0.162	0.128	0.002	0.120	0.108	0.000	0.067	0.064
	SKEW-NORMAL	-0.001	0.162	0.129	0.002	0.121	0.109	0.001	0.067	0.064
	CORRECTED SCORE	-0.002	0.152	0.135	-0.003	0.123	0.110	0.000	0.069	0.064
	CONDITIONAL SCORE	-0.001	0.148	0.134	-0.002	0.121	0.110	0.000	0.069	0.064
	SIMEX	-0.001	0.155	0.135	-0.001	0.126	0.116	0.001	0.075	0.069
0.5			$n = 10$			$n = 20$			$n = 50$	
	NAIVE	0.001	0.283	0.255	0.000	0.235	0.226	0.002	0.163	0.137
	LIKELIHOOD	0.004	0.275	0.227	-0.001	0.187	0.176	0.002	0.112	0.110
	SKEW-NORMAL	0.001	0.276	0.228	-0.001	0.189	0.177	0.003	0.112	0.110
	CORRECTED SCORE	0.006	0.271	0.228	-0.003	0.190	0.175	0.003	0.112	0.110
	CONDITIONAL SCORE	0.005	0.270	0.227	-0.003	0.189	0.174	0.003	0.112	0.110
	SIMEX	0.004	0.274	0.241	-0.003	0.198	0.188	0.003	0.118	0.116
0.9			$n = 10$			$n = 20$			$n = 50$	
	NAIVE	0.003	0.385	0.436	-0.024	0.376	0.375	-0.007	0.202	0.221
	LIKELIHOOD	0.004	0.353	0.290	-0.011	0.230	0.215	0.003	0.143	0.139
	SKEW-NORMAL	0.008	0.349	0.289	-0.011	0.232	0.216	0.003	0.144	0.139
	CORRECTED SCORE	0.005	0.346	0.292	-0.012	0.231	0.215	0.003	0.144	0.139
	CONDITIONAL SCORE	0.005	0.345	0.291	-0.011	0.231	0.215	0.003	0.144	0.139
	SIMEX	0.005	0.354	0.309	-0.012	0.241	0.229	0.003	0.151	0.147
1.5			$n = 10$			$n = 20$			$n = 50$	
	NAIVE	-0.014	0.460	0.711	0.015	0.380	0.570	0.015	0.220	0.350
	LIKELIHOOD	-0.015	0.420	0.374	0.008	0.294	0.276	0.008	0.177	0.177
	SKEW-NORMAL	-0.018	0.425	0.373	0.007	0.296	0.278	0.009	0.175	0.176
	CORRECTED SCORE	-0.013	0.420	0.370	0.009	0.296	0.277	0.008	0.177	0.177
	CONDITIONAL SCORE	-0.014	0.419	0.370	0.009	0.295	0.277	0.008	0.177	0.177
	SIMEX	-0.013	0.432	0.396	0.011	0.310	0.296	0.008	0.186	0.186

Table S2: Bias and standard deviation (SD) of the estimates of β_0, and average of the estimated standard errors (SE) obtained from naive analysis, likelihood analysis under a Normal or a Skew-Normal specification of the distribution of ξ, corrected score, conditional score, SIMEX, on the basis of $1,000$ replicates of simulation scenario i). Underlying risk as a mixture of Normals.

τ^2	Method	Bias	SD	SE	Bias	SD	SE	Bias	SD	SE
0.1			$n = 10$			$n = 20$			$n = 50$	
	NAIVE	0.006	0.141	0.078	-0.001	0.119	0.065	0.001	0.094	0.044
	LIKELIHOOD	-0.010	0.143	0.121	-0.005	0.100	0.094	-0.005	0.066	0.063
	SKEW-NORMAL	-0.015	0.147	0.121	-0.007	0.101	0.095	-0.008	0.067	0.064
	CORRECTED SCORE	-0.009	0.140	0.130	-0.001	0.101	0.096	-0.002	0.067	0.063
	CONDITIONAL SCORE	-0.008	0.141	0.129	-0.001	0.103	0.100	-0.002	0.067	0.063
	SIMEX	-0.001	0.139	0.128	-0.001	0.126	0.116	-0.001	0.070	0.066
0.5			$n = 10$			$n = 20$			$n = 50$	
	NAIVE	-0.012	0.265	0.249	0.003	0.212	0.196	0.010	0.165	0.132
	LIKELIHOOD	-0.034	0.248	0.218	-0.007	0.167	0.163	-0.002	0.107	0.108
	SKEW-NORMAL	-0.034	0.247	0.219	-0.005	0.169	0.165	-0.004	0.112	0.109
	CORRECTED SCORE	-0.028	0.248	0.218	0.001	0.169	0.163	0.004	0.108	0.107
	CONDITIONAL SCORE	-0.027	0.247	0.218	0.001	0.169	0.163	0.004	0.108	0.107
	SIMEX	-0.019	0.250	0.226	0.002	0.176	0.172	0.004	0.114	0.113
0.9			$n = 10$			$n = 20$			$n = 50$	
	NAIVE	0.013	0.501	0.510	-0.012	0.279	0.333	0.001	0.187	0.216
	LIKELIHOOD	-0.031	0.329	0.278	-0.016	0.227	0.210	-0.012	0.142	0.138
	SKEW-NORMAL	-0.030	0.328	0.278	-0.009	0.230	0.212	-0.013	0.150	0.138
	CORRECTED SCORE	-0.017	0.323	0.274	-0.008	0.227	0.210	-0.004	0.142	0.138
	CONDITIONAL SCORE	-0.016	0.323	0.274	-0.008	0.227	0.210	-0.004	0.142	0.138
	SIMEX	-0.010	0.333	0.288	-0.008	0.236	0.223	-0.004	0.150	0.145
1.5			$n = 10$			$n = 20$			$n = 50$	
	NAIVE	0.022	0.450	0.685	0.010	0.543	0.630	0.009	0.315	0.361
	LIKELIHOOD	-0.009	0.415	0.354	-0.008	0.276	0.267	-0.003	0.179	0.175
	SKEW-NORMAL	-0.010	0.425	0.358	-0.011	0.283	0.266	-0.004	0.180	0.174
	CORRECTED SCORE	0.009	0.411	0.352	0.000	0.278	0.265	0.005	0.179	0.174
	CONDITIONAL SCORE	0.008	0.411	0.352	0.001	0.278	0.265	0.005	0.179	0.174
	SIMEX	0.018	0.420	0.374	0.002	0.292	0.282	0.005	0.189	0.184

Table S3: Bias and standard deviation (SD) of the estimates of β_0, and average of the estimated standard errors (SE) obtained from naive analysis, likelihood analysis under a Normal or a Skew-Normal specification of the distribution of ξ, corrected score, conditional score, SIMEX, on the basis of $1,000$ replicates of simulation scenario i). Underlying risk as a Skew-Normal.

τ^2	Method	Bias	SD	SE	Bias	SD	SE	Bias	SD	SE
0.1			$n = 10$			$n = 20$			$n = 50$	
	NAIVE	0.057	0.182	0.087	0.074	0.280	0.113	0.069	0.160	0.055
	LIKELIHOOD	-0.008	0.188	0.152	-0.011	0.146	0.128	0.001	0.081	0.080
	SKEW-NORMAL	-0.007	0.191	0.156	-0.011	0.150	0.131	0.001	0.082	0.080
	CORRECTED SCORE	-0.013	0.192	0.169	-0.016	0.158	0.139	0.000	0.086	0.078
	CONDITIONAL SCORE	-0.004	0.184	0.164	-0.008	0.153	0.135	0.000	0.085	0.077
	SIMEX	0.009	0.187	0.168	0.005	0.156	0.147	0.010	0.090	0.086
0.5			$n = 10$			$n = 20$			$n = 50$	
	NAIVE	0.057	0.354	0.264	0.100	0.328	0.236	0.058	0.246	0.147
	LIKELIHOOD	-0.021	0.347	0.274	-0.006	0.246	0.215	-0.002	0.141	0.134
	SKEW-NORMAL	-0.017	0.354	0.269	-0.008	0.249	0.214	-0.002	0.142	0.135
	CORRECTED SCORE	-0.024	0.340	0.282	-0.005	0.245	0.211	-0.004	0.142	0.132
	CONDITIONAL SCORE	-0.023	0.341	0.274	-0.001	0.241	0.206	-0.004	0.142	0.131
	SIMEX	0.004	0.343	0.294	0.022	0.252	0.231	0.006	0.148	0.143
0.9			$n = 10$			$n = 20$			$n = 50$	
	NAIVE	0.068	0.481	0.448	0.082	0.452	0.390	0.091	0.284	0.237
	LIKELIHOOD	-0.024	0.446	0.364	-0.003	0.303	0.270	0.005	0.179	0.175
	SKEW-NORMAL	-0.023	0.456	0.356	-0.003	0.304	0.274	0.006	0.179	0.172
	CORRECTED SCORE	-0.016	0.430	0.360	-0.004	0.306	0.268	0.006	0.182	0.169
	CONDITIONAL SCORE	-0.011	0.427	0.353	-0.002	0.307	0.263	0.007	0.181	0.168
	SIMEX	0.010	0.439	0.382	0.022	0.313	0.288	0.017	0.187	0.182
1.5			$n = 10$			$n = 20$			$n = 50$	
	NAIVE	0.045	0.570	0.685	0.075	0.573	0.636	0.080	0.376	0.367
	LIKELIHOOD	-0.045	0.584	0.449	-0.003	0.386	0.345	0.004	0.227	0.218
	SKEW-NORMAL	-0.058	0.588	0.455	0.000	0.385	0.348	0.005	0.227	0.216
	CORRECTED SCORE	-0.037	0.557	0.455	0.000	0.387	0.342	0.002	0.229	0.213
	CONDITIONAL SCORE	-0.034	0.554	0.450	0.004	0.384	0.336	0.003	0.227	0.211
	SIMEX	-0.014	0.576	0.481	0.032	0.394	0.367	0.012	0.237	0.228

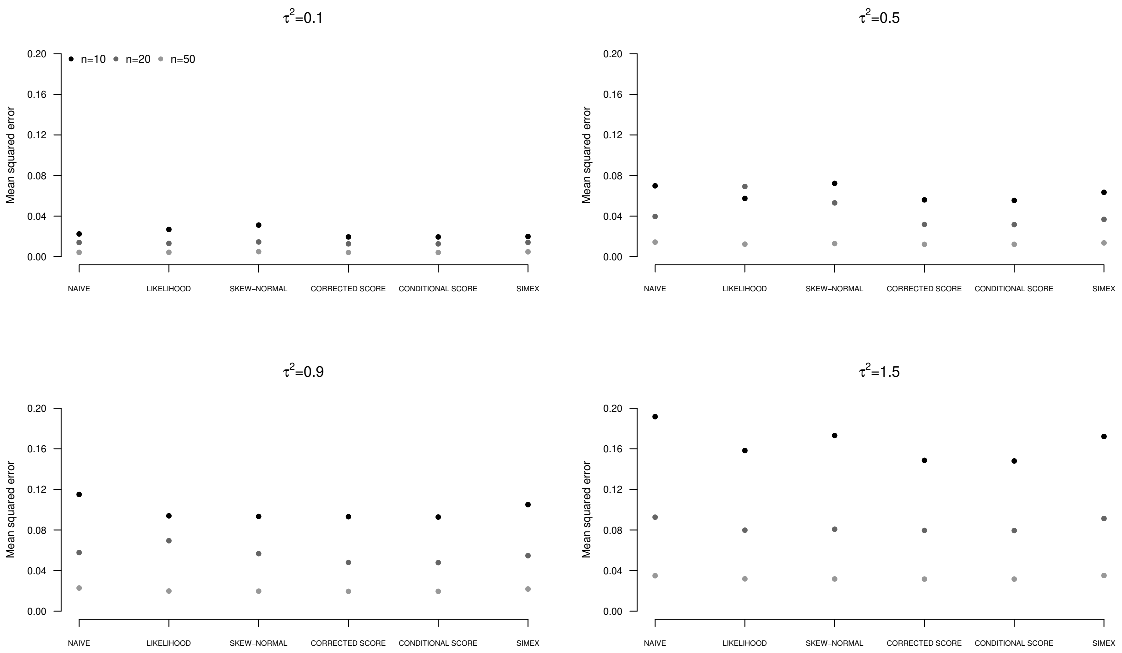

Figure S1: Mean squared error of the estimators of β_0 obtained from naive analysis, likelihood analysis under a Normal or a Skew-Normal specification of the distribution of ξ, corrected score, conditional score, SIMEX, on the basis of $1,000$ replicates of simulation scenario i). Underlying risk normally distributed.

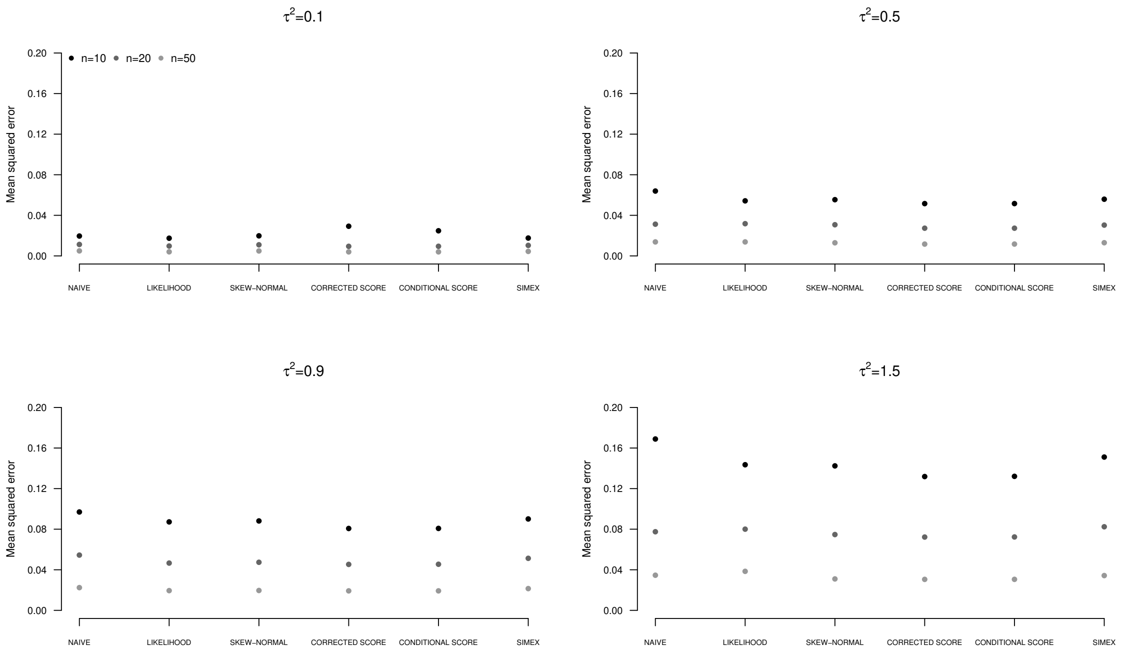

Figure S2: Mean squared error of the estimators of β_0 obtained from naive analysis, likelihood analysis under a Normal or a Skew-Normal specification of the distribution of ξ, corrected score, conditional score, SIMEX, on the basis of $1,000$ replicates of simulation scenario i). Underlying risk distributed as a mixture of Normals.

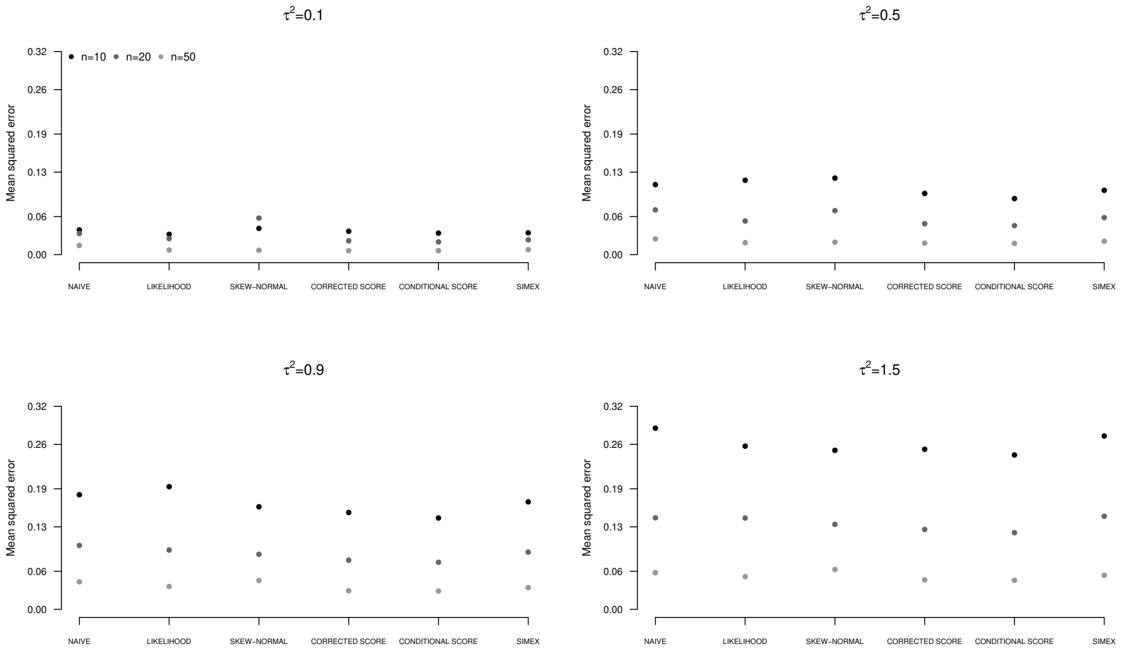

Figure S3: Mean squared error of the estimators of β_0 obtained from naive analysis, likelihood analysis under a Normal or a Skew-Normal specification of the distribution of ξ, corrected score, conditional score, SIMEX, on the basis of $1,000$ replicates of simulation scenario i). Underlying risk distributed as a Skew-Normal.

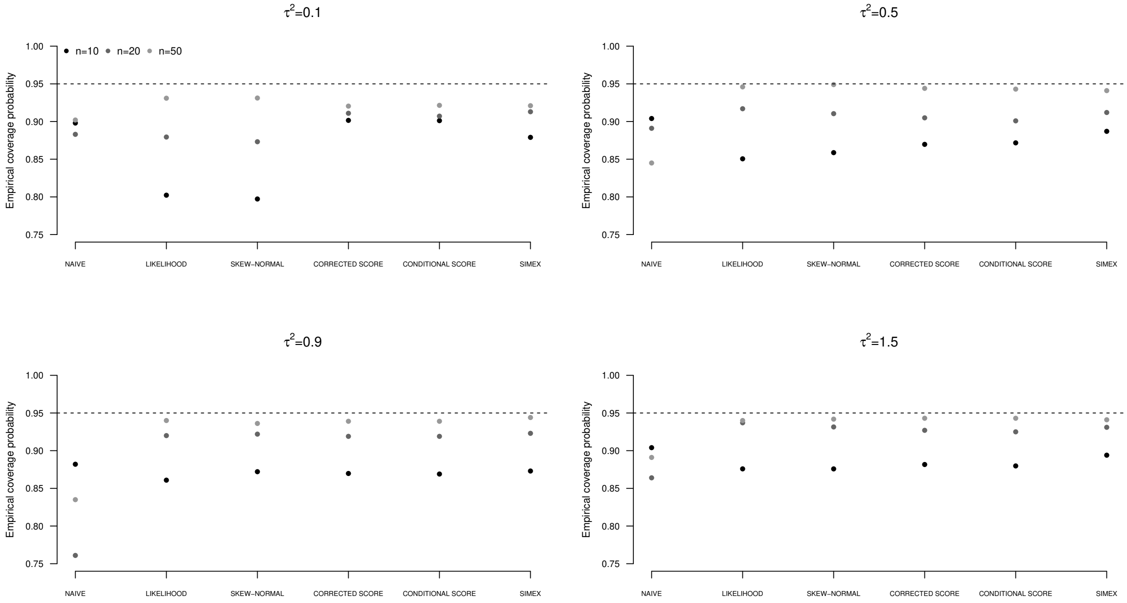

Figure S4: Empirical coverage probabilities of confidence intervals for β_0 from uncorrected approach (NAIVE), likelihood approach under a Normal specification (LIKELIHOOD) or a Skew-Normal specification (SKEW-NORMAL) for the underlying risk distribution, SIMEX, corrected score and conditional score, on the basis of $1,000$ replicates of simulation scenario i). Underlying risk normally distributed.

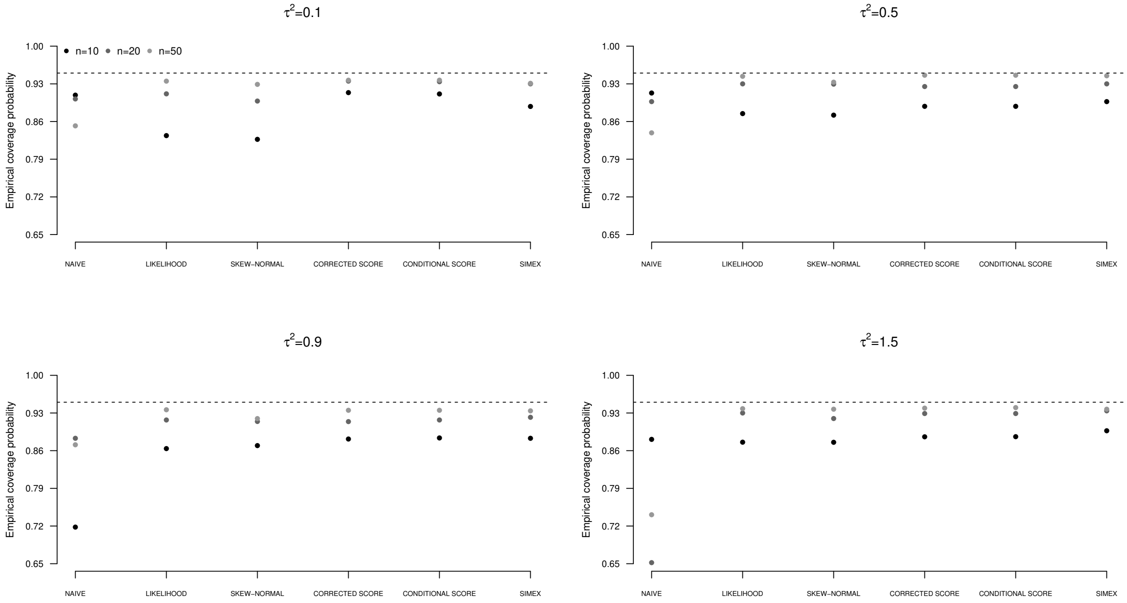

Figure S5: Empirical coverage probabilities of confidence intervals for β_0 from uncorrected approach (NAIVE), likelihood approach under a Normal specification (LIKELIHOOD) or a Skew-Normal specification (SKEW-NORMAL) for the underlying risk distribution, SIMEX, corrected score and conditional score, on the basis of $1,000$ replicates of simulation scenario i). Underlying risk distributed as a mixture of Normals.

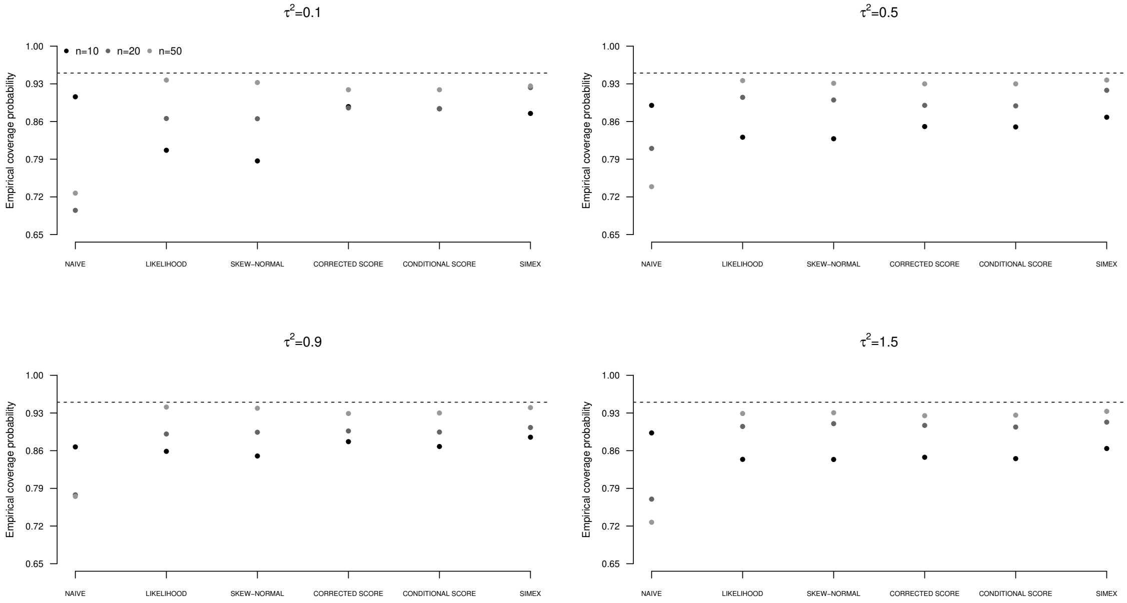

Figure S6: Empirical coverage probabilities of confidence intervals for β_0 from uncorrected approach (NAIVE), likelihood approach under a Normal specification (LIKELIHOOD) or a Skew-Normal specification (SKEW-NORMAL) for the underlying risk distribution, SIMEX, corrected score and conditional score, on the basis of $1,000$ replicates of simulation scenario i). Underlying risk distributed as a Skew-Normal.

Table S4: Empirical coverage probabilities (multiplied by 1,000) of confidence intervals for β_0 and associated Monte Carlo standard error (in parentheses, multiplied by 1,000) obtained from naive analysis, likelihood analysis under a Normal or a Skew-Normal specification of the distribution of ξ, corrected score, conditional score, SIMEX, on the basis of $1,000$ replicates of simulation scenario i). Underlying risk distributed as a Normal, Mixture of Normals, or Skew-Normal.

		n			n			n		
τ^2	Method	10	20	50	10	20	50	10	20	50
0.1			Normal			Mixture			Skew-Normal	
	NAIVE	898 (10)	883 (10)	902 (9)	909 (9)	902 (9)	852 (11)	906 (9)	695 (15)	727 (14)
	LIKELIHOOD	802 (13)	879 (10)	931 (8)	834 (12)	911 (9)	935 (8)	807 (13)	866 (11)	937 (8)
	SKEW-NORMAL	797 (14)	873 (11)	931 (8)	827 (13)	898 (11)	929 (10)	787 (14)	865 (11)	932 (8)
	CORRECTED SCORE	902 (10)	911 (10)	920 (9)	888 (11)	885 (11)	919 (9)	914 (9)	935 (8)	937 (8)
	CONDITIONAL SCORE	901 (10)	907 (10)	921 (9)	884 (11)	884 (11)	919 (9)	911 (10)	934 (8)	937 (8)
	SIMEX	879 (10)	913 (9)	921 (9)	875 (10)	923 (8)	926 (8)	888 (10)	930 (8)	931 (8)
0.1			Normal			Mixture			Skew-Normal	
	NAIVE	904 (9)	891 (10)	845 (11)	913 (9)	897 (10)	839 (12)	890 (10)	810 (12)	739 (14)
	LIKELIHOOD	851 (11)	917 (9)	946 (7)	875 (10)	930 (8)	944 (7)	831 (12)	905 (9)	936 (8)
	SKEW-NORMAL	859 (12)	910 (9)	949 (7)	872 (11)	929 (9)	933 (10)	828 (13)	900 (10)	931 (8)
	CORRECTED SCORE	870 (11)	905 (9)	944 (7)	888 (10)	925 (8)	946 (7)	851 (11)	890 (10)	930 (8)
	CONDITIONAL SCORE	872 (11)	901 (9)	943 (7)	888 (10)	925 (8)	946 (7)	850 (11)	889 (10)	930 (8)
	SIMEX	887 (10)	912 (9)	941 (7)	897 (10)	930 (8)	945 (7)	868 (11)	918 (9)	937 (8)
0.1			Normal			Mixture			Skew-Normal	
	NAIVE	882 (10)	761 (13)	835 (12)	718 (14)	883 (10)	871 (11)	867 (11)	778 (13)	775 (13)
	LIKELIHOOD	861 (11)	920 (9)	940 (8)	864 (11)	917 (9)	936 (8)	859 (11)	891 (10)	941 (7)
	SKEW-NORMAL	872 (11)	922 (9)	936 (8)	869 (11)	914 (10)	920 (11)	850 (12)	894 (10)	939 (8)
	CORRECTED SCORE	870 (11)	919 (9)	939 (8)	882 (10)	914 (9)	935 (8)	877 (10)	897 (10)	929 (8)
	CONDITIONAL SCORE	869 (11)	919 (9)	939 (8)	884 (10)	917 (9)	935 (8)	868 (11)	895 (10)	930 (8)
	SIMEX	873 (11)	923 (8)	944 (7)	883 (10)	922 (8)	934 (8)	885 (10)	903 (9)	940 (8)
0.1			Normal			Mixture			Skew-Normal	
	NAIVE	904 (9)	864 (11)	891 (10)	881 (10)	652 (15)	741 (14)	893 (10)	770 (13)	727 (14)
	LIKELIHOOD	876 (10)	937 (8)	940 (8)	876 (10)	930 (8)	938 (8)	844 (11)	905 (9)	929 (8)
	SKEW-NORMAL	876 (11)	931 (8)	942 (7)	876 (11)	920 (10)	937 (10)	844 (12)	910 (9)	930 (8)
	CORRECTED SCORE	882 (10)	927 (8)	943 (7)	886 (10)	929 (8)	939 (8)	848 (11)	907 (9)	925 (8)
	CONDITIONAL SCORE	880 (10)	925 (8)	943 (7)	886 (10)	929 (8)	940 (8)	845 (12)	904 (9)	926 (8)
	SIMEX	894 (10)	931 (8)	941 (7)	897 (10)	934 (8)	937 (8)	864 (11)	913 (9)	933 (8)

Table S5: Bias and standard deviation (SD) of the estimates of β_1, and average of the estimated standard errors (SE) obtained from naive analysis, likelihood analysis under a Normal or a Skew-Normal specification of the distribution of ξ, corrected score, conditional score, SIMEX, on the basis of $1,000$ replicates of simulation scenario i). Underlying risk normally distributed.

τ^2	Method	Bias	SD	SE	Bias	SD	SE	Bias	SD	SE
0.1			$n = 10$			$n = 20$			$n = 50$	
	NAIVE	-0.070	0.189	0.083	-0.092	0.146	0.088	-0.055	0.083	0.048
	LIKELIHOOD	0.028	0.209	0.144	0.023	0.152	0.129	-0.003	0.076	0.073
	SKEW-NORMAL	0.032	0.216	0.142	0.024	0.156	0.131	-0.003	0.077	0.073
	CORRECTED SCORE	-0.001	0.221	0.153	-0.026	0.157	0.130	-0.029	0.082	0.074
	CONDITIONAL SCORE	-0.016	0.204	0.149	-0.033	0.149	0.126	-0.029	0.080	0.073
	SIMEX	-0.013	0.205	0.139	-0.005	0.154	0.126	0.000	0.088	0.079
0.5			$n = 10$			$n = 20$			$n = 50$	
	NAIVE	-0.051	0.302	0.255	-0.073	0.240	0.226	-0.035	0.161	0.137
	LIKELIHOOD	0.042	0.339	0.256	0.024	0.230	0.201	0.004	0.122	0.120
	SKEW-NORMAL	0.044	0.363	0.259	0.025	0.231	0.200	0.003	0.123	0.121
	CORRECTED SCORE	0.008	0.322	0.234	-0.028	0.228	0.185	-0.028	0.122	0.116
	CONDITIONAL SCORE	0.002	0.319	0.231	-0.033	0.226	0.183	-0.029	0.121	0.117
	SIMEX	-0.003	0.316	0.245	-0.007	0.233	0.193	0.004	0.126	0.122
0.9			$n = 10$			$n = 20$			$n = 50$	
	NAIVE	-0.054	0.399	0.436	-0.004	0.378	0.375	-0.063	0.197	0.221
	LIKELIHOOD	0.049	0.446	0.328	0.017	0.277	0.232	-0.002	0.156	0.150
	SKEW-NORMAL	0.042	0.428	0.326	0.017	0.277	0.231	-0.004	0.156	0.151
	CORRECTED SCORE	0.003	0.406	0.299	-0.014	0.271	0.219	-0.040	0.152	0.144
	CONDITIONAL SCORE	-0.001	0.402	0.298	-0.017	0.269	0.220	-0.040	0.153	0.145
	SIMEX	-0.005	0.404	0.317	0.011	0.276	0.231	-0.005	0.159	0.152
1.5			$n = 10$			$n = 20$			$n = 50$	
	NAIVE	-0.080	0.492	0.711	-0.072	0.367	0.570	-0.061	0.217	0.350
	LIKELIHOOD	0.025	0.519	0.417	0.047	0.350	0.313	-0.006	0.205	0.190
	SKEW-NORMAL	0.024	0.522	0.417	0.042	0.342	0.315	-0.011	0.203	0.190
	CORRECTED SCORE	-0.018	0.504	0.378	-0.012	0.337	0.286	-0.042	0.197	0.182
	CONDITIONAL SCORE	-0.025	0.496	0.376	-0.017	0.329	0.286	-0.043	0.196	0.182
	SIMEX	-0.023	0.499	0.398	0.007	0.335	0.299	-0.008	0.203	0.190

Table S6: Bias and standard deviation (SD) of the estimates of β_1, and average of the estimated standard errors (SE) obtained from naive analysis, likelihood analysis under a Normal or a Skew-Normal specification of the distribution of ξ, corrected score, conditional score, SIMEX, on the basis of $1,000$ replicates of simulation scenario i). Underlying risk distributed as a mixture of Normals.

τ^2	Method	Bias	SD	SE	Bias	SD	SE	Bias	SD	SE
0.1			$n = 10$			$n = 20$			$n = 50$	
	NAIVE	-0.100	0.220	0.078	-0.131	0.175	0.065	-0.095	0.130	0.044
	LIKELIHOOD	0.058	0.289	0.184	0.005	0.166	0.143	-0.004	0.105	0.100
	SKEW-NORMAL	0.056	0.302	0.181	0.001	0.168	0.144	-0.015	0.104	0.100
	CORRECTED SCORE	0.033	0.280	0.207	-0.022	0.174	0.150	-0.022	0.108	0.101
	CONDITIONAL SCORE	0.011	0.257	0.194	-0.028	0.166	0.145	-0.024	0.106	0.097
	SIMEX	-0.008	0.255	0.187	-0.024	0.180	0.152	-0.007	0.112	0.105
0.5			$n = 10$			$n = 20$			$n = 50$	
	NAIVE	-0.105	0.401	0.249	-0.104	0.292	0.196	-0.068	0.233	0.132
	LIKELIHOOD	0.051	0.474	0.354	0.021	0.282	0.254	0.013	0.175	0.172
	SKEW-NORMAL	0.060	0.524	0.347	0.021	0.290	0.253	0.007	0.177	0.170
	CORRECTED SCORE	0.023	0.463	0.340	0.002	0.286	0.248	-0.016	0.174	0.169
	CONDITIONAL SCORE	0.012	0.451	0.326	-0.007	0.280	0.242	-0.015	0.173	0.167
	SIMEX	-0.023	0.429	0.336	-0.003	0.289	0.256	0.011	0.181	0.177s
0.9			$n = 10$			$n = 20$			$n = 50$	
	NAIVE	-0.158	0.658	0.510	-0.140	0.365	0.333	-0.097	0.253	0.216
	LIKELIHOOD	0.067	0.589	0.436	0.008	0.358	0.323	-0.007	0.228	0.217
	SKEW-NORMAL	0.066	0.614	0.431	0.008	0.373	0.329	-0.006	0.227	0.214
	CORRECTED SCORE	0.015	0.538	0.407	-0.023	0.348	0.313	-0.035	0.226	0.212
	CONDITIONAL SCORE	0.009	0.529	0.395	-0.028	0.343	0.310	-0.036	0.224	0.210
	SIMEX	-0.012	0.537	0.419	-0.019	0.352	0.327	-0.007	0.233	0.222
1.5			$n = 10$			$n = 20$			$n = 50$	
	NAIVE	-0.082	0.685	0.685	-0.073	0.675	0.630	-0.086	0.394	0.361
	LIKELIHOOD	0.088	0.800	0.554	0.025	0.457	0.404	-0.001	0.287	0.277
	SKEW-NORMAL	0.095	0.859	0.556	0.028	0.463	0.402	-0.008	0.282	0.273
	CORRECTED SCORE	0.000	0.689	0.522	-0.003	0.454	0.391	-0.029	0.281	0.268
	CONDITIONAL SCORE	-0.004	0.683	0.512	-0.011	0.446	0.386	-0.029	0.280	0.267
	SIMEX	0.000	0.719	0.543	0.003	0.460	0.410	0.007	0.290	0.281

Table S7: Bias and standard deviation (SD) of the estimates of β_1, and average of the estimated standard errors (SE) obtained from naive analysis, likelihood analysis under a Normal or a Skew-Normal specification of the distribution of ξ, corrected score, conditional score, SIMEX, on the basis of $1,000$ replicates of simulation scenario i). Underlying risk distributed as a Skew-Normal.

τ^2	Method	Bias	SD	SE	Bias	SD	SE	Bias	SD	SE
0.1			$n=10$			$n=20$			$n=50$	
	NAIVE	-0.097	0.212	0.087	-0.111	0.263	0.113	-0.099	0.167	0.055
	LIKELIHOOD	0.028	0.242	0.180	0.025	0.188	0.151	0.001	0.103	0.094
	SKEW-NORMAL	0.029	0.244	0.179	0.023	0.195	0.155	-0.001	0.104	0.095
	CORRECTED SCORE	-0.003	0.251	0.190	-0.011	0.213	0.172	-0.031	0.109	0.099
	CONDITIONAL SCORE	-0.017	0.242	0.184	-0.029	0.197	0.155	-0.031	0.107	0.095
	SIMEX	-0.026	0.235	0.177	-0.016	0.199	0.160	-0.016	0.113	0.101
0.5			$n=10$			$n=20$			$n=50$	
	NAIVE	-0.085	0.370	0.264	-0.144	0.329	0.236	-0.084	0.231	0.147
	LIKELIHOOD	0.051	0.420	0.311	0.036	0.295	0.250	0.003	0.166	0.150
	SKEW-NORMAL	0.046	0.418	0.300	0.034	0.299	0.250	0.001	0.168	0.151
	CORRECTED SCORE	0.021	0.408	0.296	-0.015	0.291	0.242	-0.037	0.164	0.148
	CONDITIONAL SCORE	0.012	0.393	0.285	-0.022	0.281	0.230	-0.037	0.162	0.145
	SIMEX	-0.010	0.388	0.304	-0.021	0.285	0.247	-0.015	0.170	0.153
0.9			$n=10$			$n=20$			$n=50$	
	NAIVE	-0.079	0.496	0.448	-0.123	0.406	0.390	-0.108	0.286	0.237
	LIKELIHOOD	0.078	0.567	0.412	0.016	0.363	0.308	-0.002	0.206	0.192
	SKEW-NORMAL	0.074	0.595	0.408	0.012	0.368	0.307	-0.005	0.206	0.190
	CORRECTED SCORE	0.015	0.504	0.370	-0.034	0.353	0.287	-0.044	0.201	0.184
	CONDITIONAL SCORE	0.006	0.497	0.358	-0.043	0.342	0.278	-0.045	0.199	0.182
	SIMEX	0.004	0.530	0.395	-0.038	0.342	0.295	-0.019	0.206	0.190
1.5			$n=10$			$n=20$			$n=50$	
	NAIVE	-0.105	0.601	0.685	-0.110	0.518	0.636	-0.114	0.337	0.367
	LIKELIHOOD	0.046	0.708	0.506	0.048	0.443	0.393	-0.001	0.250	0.234
	SKEW-NORMAL	0.051	0.722	0.505	0.035	0.442	0.394	-0.002	0.248	0.234
	CORRECTED SCORE	-0.007	0.637	0.460	-0.017	0.421	0.366	-0.043	0.242	0.224
	CONDITIONAL SCORE	-0.012	0.630	0.451	-0.024	0.411	0.355	-0.044	0.240	0.221
	SIMEX	-0.014	0.654	0.496	-0.027	0.409	0.376	-0.018	0.245	0.232

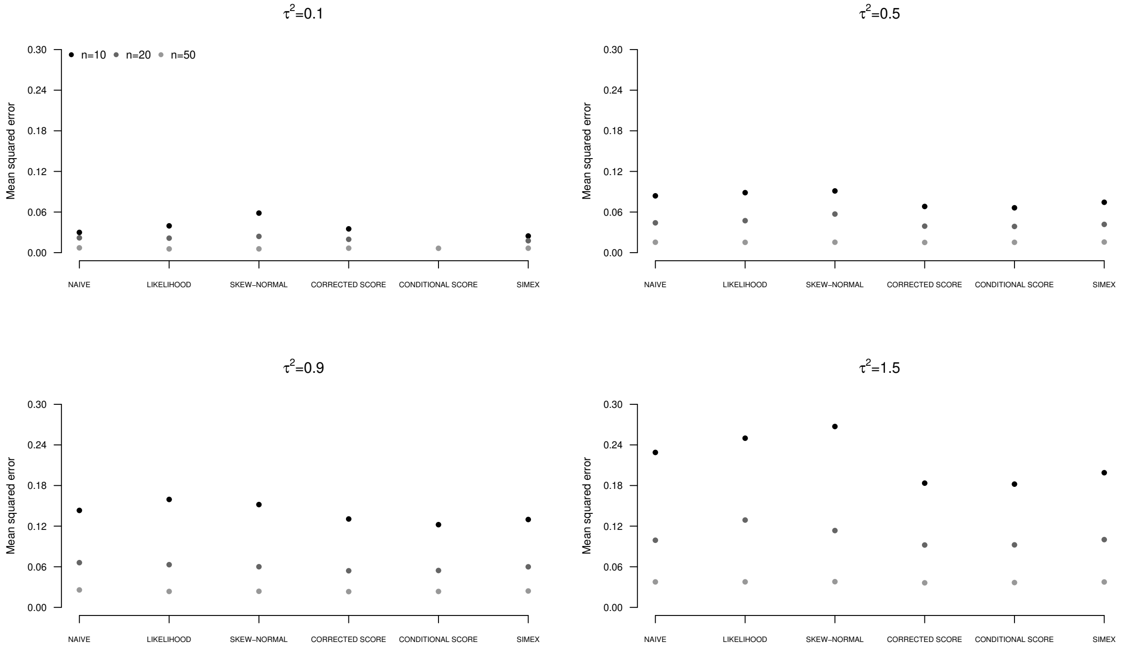

Figure S7: Mean squared error of the estimators of β_1 obtained from naive analysis, likelihood analysis under a Normal or a Skew-Normal specification of the distribution of ξ, corrected score, conditional score, SIMEX, on the basis of $1,000$ replicates of simulation scenario i). Underlying risk normally distributed.

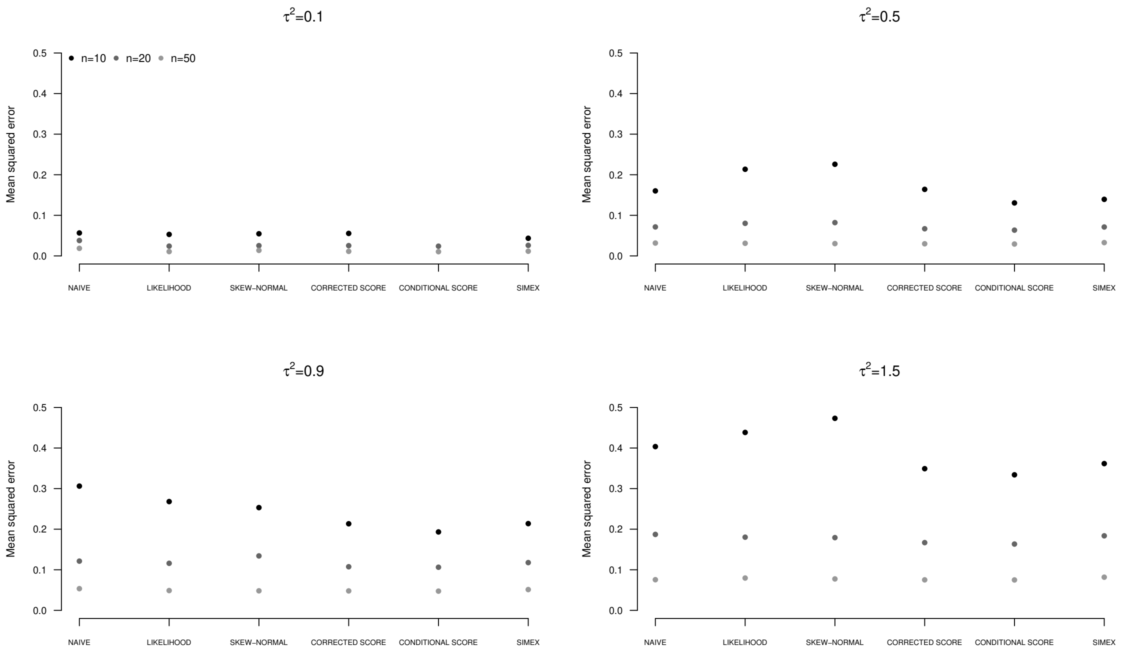

Figure S8: Mean squared error of the estimators of β_1 obtained from naive analysis, likelihood analysis under a Normal or a Skew-Normal specification of the distribution of ξ, corrected score, conditional score, SIMEX, on the basis of $1,000$ replicates of simulation scenario i). Underlying risk distributed as a mixture of Normals.

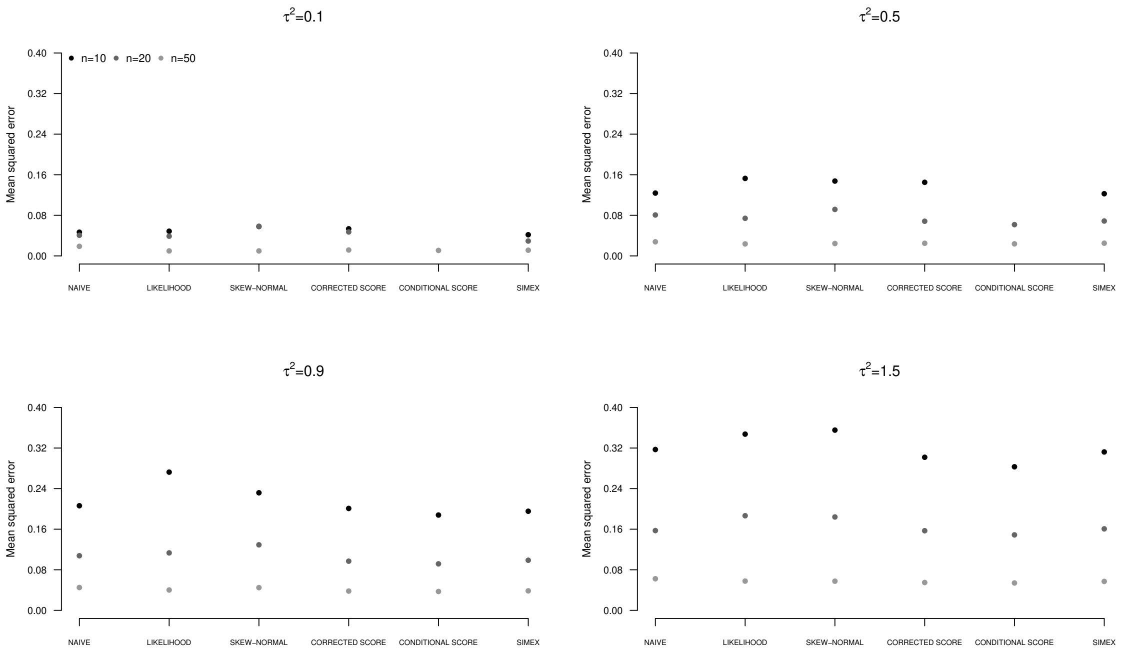

Figure S9: Mean squared error of the estimators of β_1 obtained from naive analysis, likelihood analysis under a Normal or a Skew-Normal specification of the distribution of ξ, corrected score, conditional score, SIMEX, on the basis of $1,000$ replicates of simulation scenario i). Underlying risk distributed as a Skew-Normal.

Table S8: Empirical coverage probabilities (multiplied by 1,000) of confidence intervals for β_1 and associated Monte Carlo standard error (in parentheses, multiplied by 1,000) obtained from naive analysis, likelihood analysis under a Normal or a Skew-Normal specification of the distribution of ξ, corrected score, conditional score, SIMEX, on the basis of $1,000$ replicates of simulation scenario i). Underlying risk distributed as a Normal, Skew-Normal, or Mixture of Normals.

			n			n			n	
τ^2	Method	10	20	50	10	20	50	10	20	50
0.1			Normal			Mixture			Skew-Normal	
	NAIVE	823 (12)	748 (14)	773 (13)	860 (11)	741 (14)	728 (14)	830 (12)	669 (15)	620 (15)
	LIKELIHOOD	770 (13)	873 (11)	926 (8)	742 (14)	861 (11)	923 (8)	774 (13)	855 (11)	928 (8)
	SKEW-NORMAL	759 (15)	863 (11)	922 (9)	719 (15)	864 (12)	897 (12)	779 (14)	862 (12)	925 (8)
	CORRECTED SCORE	818 (13)	848 (12)	893 (10)	831 (12)	878 (11)	914 (9)	818 (13)	866 (12)	906 (9)
	CONDITIONAL SCORE	824 (13)	843 (12)	896 (10)	817 (13)	868 (11)	908 (9)	808 (14)	848 (12)	899 (10)
	SIMEX	770 (13)	863 (11)	912 (9)	809 (12)	866 (11)	926 (8)	804 (13)	869 (11)	905 (9)
0.5			Normal			Mixture			Skew-Normal	
	NAIVE	887 (10)	834 (12)	821 (12)	885 (10)	845 (11)	789 (13)	875 (10)	762 (13)	729 (14)
	LIKELIHOOD	830 (12)	889 (10)	935 (8)	797 (13)	910 (9)	927 (8)	814 (12)	891 (10)	902 (9)
	SKEW-NORMAL	822 (13)	883 (11)	930 (8)	789 (14)	902 (11)	924 (10)	807 (13)	900 (10)	901 (10)
	CORRECTED SCORE	823 (12)	855 (11)	918 (9)	814 (12)	886 (10)	923 (8)	812 (12)	884 (10)	880 (10)
	CONDITIONAL SCORE	817 (12)	840 (12)	923 (8)	805 (13)	880 (10)	915 (9)	802 (13)	862 (11)	885 (10)
	SIMEX	828 (12)	862 (11)	928 (8)	822 (12)	888 (10)	927 (8)	848 (11)	878 (10)	899 (10)
0.9			Normal			Mixture			Skew-Normal	
	NAIVE	876 (10)	773 (13)	822 (12)	775 (13)	850 (11)	849 (11)	876 (10)	797 (13)	738 (14)
	LIKELIHOOD	840 (12)	880 (10)	935 (8)	818 (12)	902 (9)	927 (8)	829 (12)	878 (10)	924 (8)
	SKEW-NORMAL	839 (12)	886 (10)	934 (8)	815 (13)	895 (11)	929 (10)	826 (13)	876 (11)	920 (9)
	CORRECTED SCORE	824 (12)	866 (11)	912 (9)	812 (12)	896 (10)	916 (9)	815 (12)	851 (11)	908 (9)
	CONDITIONAL SCORE	817 (12)	865 (11)	915 (9)	801 (13)	893 (10)	919 (9)	800 (13)	844 (11)	903 (9)
	SIMEX	836 (12)	881 (10)	927 (8)	823 (12)	902 (9)	924 (8)	848 (11)	870 (11)	917 (9)
1.5			Normal			Mixture			Skew-Normal	
	NAIVE	885 (10)	852 (11)	867 (11)	886 (10)	746 (14)	769 (13)	890 (10)	800 (13)	760 (14)
	LIKELIHOOD	850 (11)	892 (10)	918 (9)	793 (13)	897 (10)	929 (8)	835 (12)	896 (10)	923 (8)
	SKEW-NORMAL	848 (12)	897 (10)	922 (9)	801 (13)	894 (11)	939 (9)	831 (12)	889 (10)	924 (8)
	CORRECTED SCORE	824 (12)	876 (10)	903 (9)	803 (13)	879 (10)	926 (8)	806 (13)	872 (11)	911 (9)
	CONDITIONAL SCORE	814 (12)	866 (11)	904 (9)	789 (13)	877 (10)	922 (8)	800 (13)	861 (11)	906 (9)
	SIMEX	834 (12)	887 (10)	917 (9)	812 (12)	895 (10)	933 (8)	847 (11)	898 (10)	920 (9)

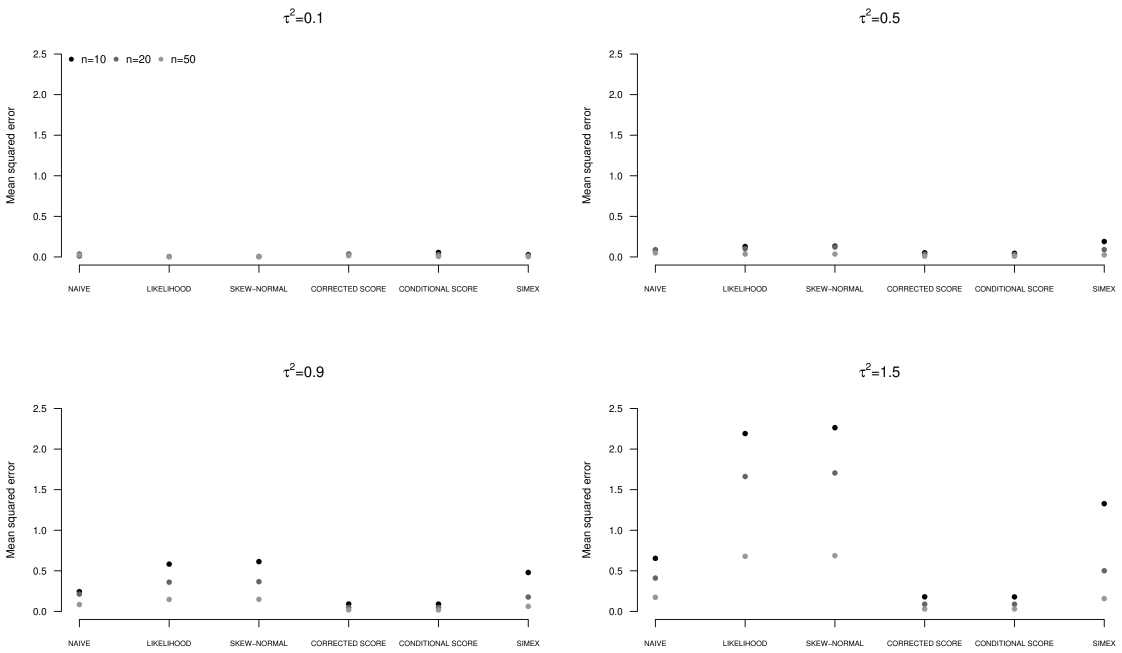

Figure S10: Mean squared error of the estimators of τ^2 obtained from naive analysis, likelihood analysis under a Normal or a Skew-Normal specification of the distribution of ξ, corrected score, conditional score, SIMEX, on the basis of $1,000$ replicates of simulation scenario i). Underlying risk normally distributed.

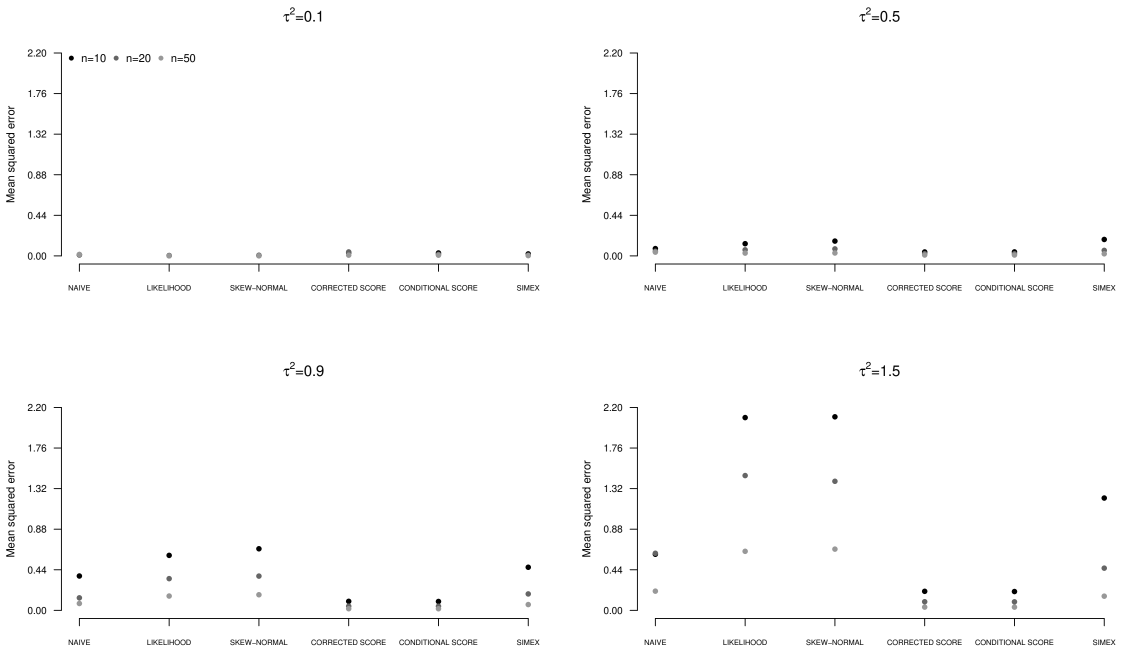

Figure S11: Mean squared error of the estimators of τ^2 obtained from naive analysis, likelihood analysis under a Normal or a Skew-Normal specification of the distribution of ξ, corrected score, conditional score, SIMEX, on the basis of $1,000$ replicates of simulation scenario i). Underlying risk distributed as a mixture of Normals.

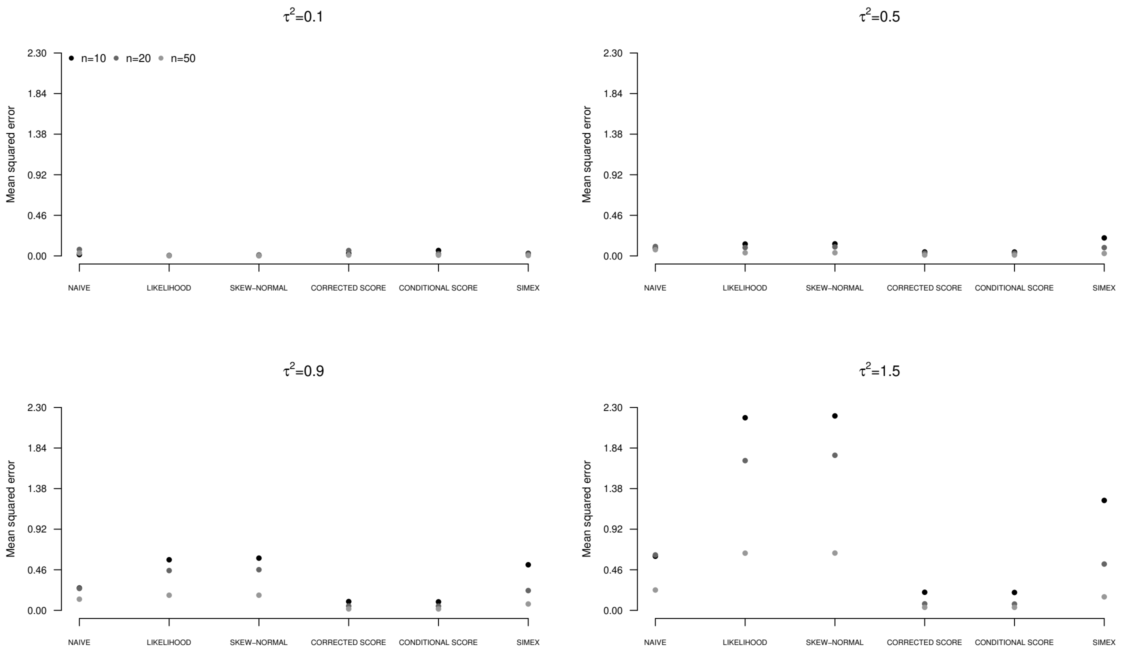

Figure S12: Mean squared error of the estimators of τ^2 obtained from naive analysis, likelihood analysis under a Normal or a Skew-Normal specification of the distribution of ξ, corrected score, conditional score, SIMEX, on the basis of $1,000$ replicates of simulation scenario i). Underlying risk distributed as a Skew-Normal.

Table S9: Bias and standard deviation (SD) of the estimates of β_0, β_1, τ^2, and average of the estimated standard errors (SE) obtained from naive analysis, likelihood analysis under a Normal or a Skew-Normal specification of the distribution of ξ, corrected score, conditional score, SIMEX, on the basis of $1,000$ replicates of simulation scenario i), with $n = 100$. Underlying risk normally distributed.

τ^2	Method	Bias	SD	SE	Bias	SD	SE	Bias	SD	SE
0.1			β_0			β_1			τ^2	
	NAIVE	-0.003	0.053	0.051	-0.072	0.061	0.049	0.159	0.045	0.037
	LIKELIHOOD	-0.002	0.049	0.046	-0.002	0.057	0.054	-0.005	0.029	0.018
	SKEW-NORMAL	-0.002	0.049	0.047	-0.003	0.056	0.054	-0.005	0.029	0.018
	CORRECTED SCORE	-0.003	0.054	0.048	-0.005	0.066	0.057	0.012	0.043	0.081
	CONDITIONAL SCORE	-0.003	0.054	0.048	-0.005	0.066	0.057	0.012	0.043	0.081
	SIMEX	-0.003	0.054	0.051	-0.005	0.066	0.061	0.012	0.044	0.044
0.5			β_0			β_1			τ^2	
	NAIVE	0.000	0.085	0.084	-0.070	0.085	0.079	0.183	0.106	0.098
	LIKELIHOOD	0.001	0.081	0.079	-0.001	0.088	0.086	-0.034	0.087	0.119
	SKEW-NORMAL	0.001	0.081	0.078	-0.002	0.088	0.086	-0.034	0.087	0.119
	CORRECTED SCORE	0.000	0.086	0.079	-0.002	0.092	0.085	0.034	0.105	0.079
	CONDITIONAL SCORE	0.000	0.086	0.079	-0.002	0.092	0.085	0.034	0.105	0.079
	SIMEX	0.000	0.086	0.083	-0.002	0.092	0.090	0.034	0.104	0.107
0.9			β_0			β_1			τ^2	
	NAIVE	-0.004	0.105	0.106	-0.075	0.106	0.100	0.195	0.173	0.156
	LIKELIHOOD	0.000	0.099	0.100	-0.011	0.111	0.108	-0.069	0.149	0.259
	SKEW-NORMAL	0.000	0.100	0.099	-0.012	0.111	0.108	-0.069	0.149	0.259
	CORRECTED SCORE	-0.004	0.105	0.100	-0.009	0.114	0.105	0.045	0.172	0.094
	CONDITIONAL SCORE	-0.004	0.105	0.100	-0.009	0.114	0.106	0.045	0.172	0.095
	SIMEX	-0.004	0.105	0.106	-0.009	0.114	0.111	0.043	0.171	0.166
1.5			β_0			β_1			τ^2	
	NAIVE	0.006	0.134	0.133	-0.073	0.131	0.126	0.220	0.265	0.246
	LIKELIHOOD	0.014	0.127	0.126	-0.008	0.141	0.134	-0.136	0.229	0.520
	SKEW-NORMAL	0.014	0.127	0.125	-0.009	0.140	0.134	-0.137	0.229	0.521
	CORRECTED SCORE	0.007	0.134	0.125	-0.008	0.140	0.128	0.066	0.269	0.177
	CONDITIONAL SCORE	0.007	0.134	0.125	-0.008	0.140	0.128	0.066	0.269	0.17
	SIMEX	0.004	0.134	0.132	-0.008	0.140	0.136	0.053	0.260	0.257

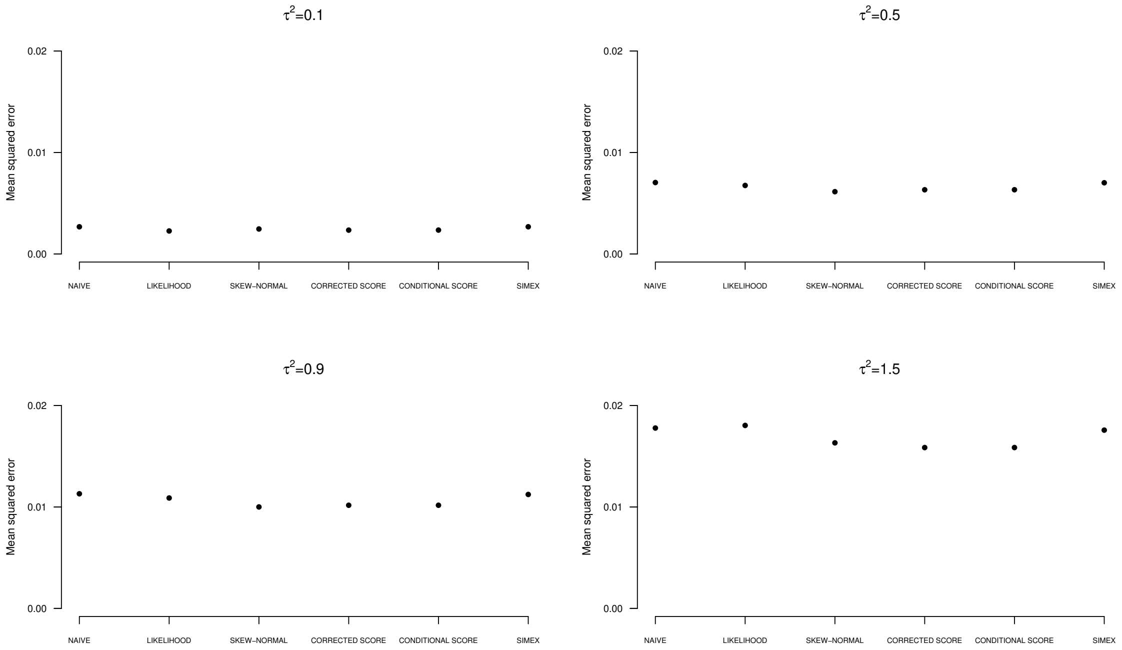

Figure S13: Mean squared error of the estimators of β_0 obtained from naive analysis, likelihood analysis under a Normal or a Skew-Normal specification of the distribution of ξ, corrected score, conditional score, SIMEX, on the basis of $1,000$ replicates of simulation scenario i), with $n = 100$. Underlying risk normally distributed.

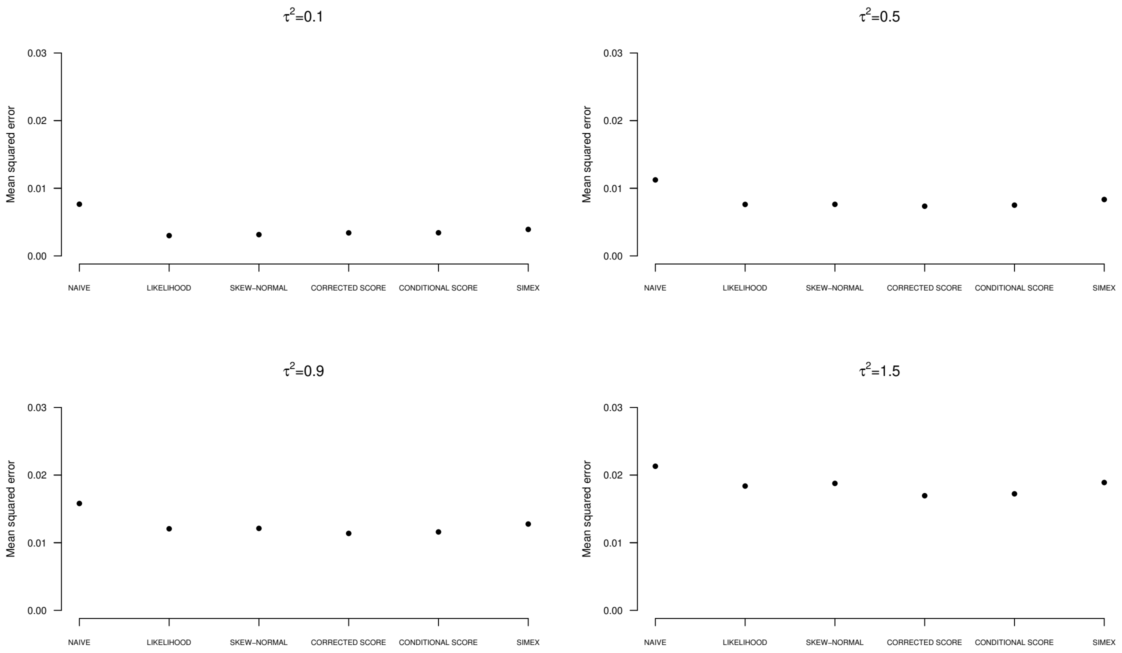

Figure S14: Mean squared error of the estimators of β_1 obtained from naive analysis, likelihood analysis under a Normal or a Skew-Normal specification of the distribution of ξ, corrected score, conditional score, SIMEX, on the basis of $1,000$ replicates of simulation scenario i), with $n = 100$. Underlying risk normally distributed.

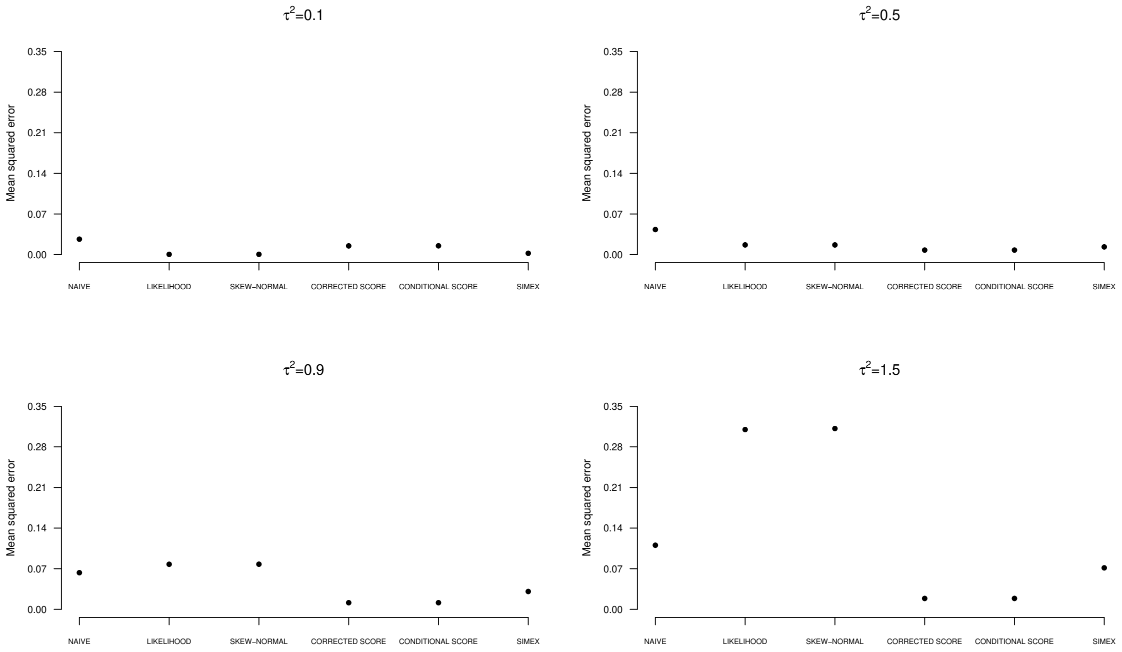

Figure S15: Mean squared error of the estimators of τ_2 obtained from naive analysis, likelihood analysis under a Normal or a Skew-Normal specification of the distribution of ξ, corrected score, conditional score, SIMEX, on the basis of $1,000$ replicates of simulation scenario i), with $n = 100$. Underlying risk normally distributed.

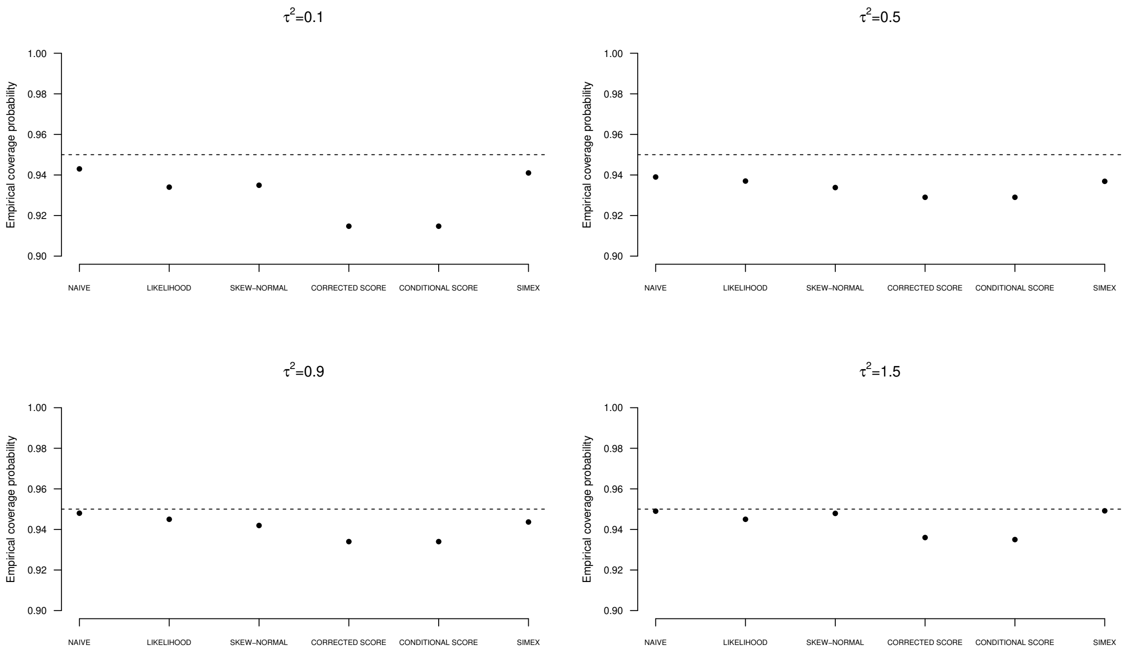

Figure S16: Empirical coverage probabilities of confidence intervals for β_0 from uncorrected approach (NAIVE), likelihood approach under a Normal specification (LIKELIHOOD) or a Skew-Normal specification (SKEW-NORMAL) for the underlying risk distribution, SIMEX, corrected score and conditional score, on the basis of $1,000$ replicates of simulation scenario i), with $n = 100$. Underlying risk normally distributed.

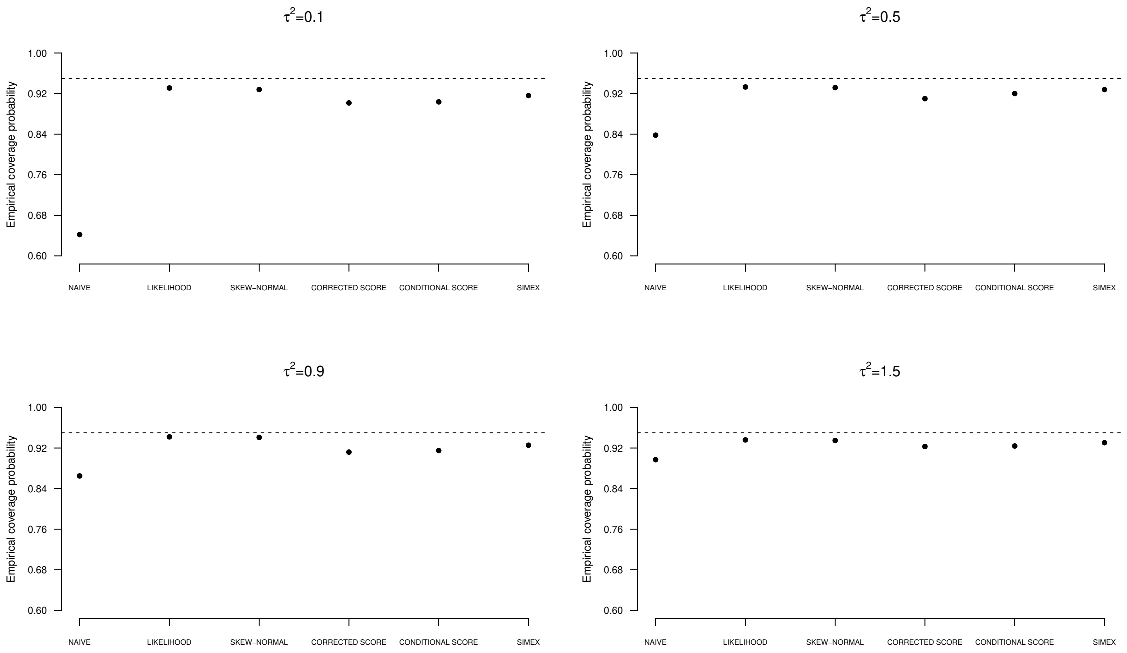

Figure S17: Empirical coverage probabilities of confidence intervals for β_1 from uncorrected approach (NAIVE), likelihood approach under a Normal specification (LIKELIHOOD) or a Skew-Normal specification (SKEW-NORMAL) for the underlying risk distribution, SIMEX, corrected score and conditional score, on the basis of $1,000$ replicates of simulation scenario i), with $n = 100$. Underlying risk normally distributed.

Web Appendix B: Simulation results for the log event rate case

This web appendix reports a portion of the results of the simulation study carried out to compare the performance of the competing approaches in control rate regression, as described in Section 4 of the main manuscript. The reference is to simulation scenario *ii*) in the main manuscript.

Table S10: Bias and standard deviation (SD) of the estimates of β_0, and average of the estimated standard errors (SE) obtained from uncorrected approach (NAIVE), likelihood analysis under a Normal or a Skew-Normal specification of the distribution of ξ, corrected score, conditional score, SIMEX, on the basis of $1,000$ replicates of simulation scenario *ii*). Underlying risk normally distributed.

τ^2	Method	Bias	SD	SE	Bias	SD	SE	Bias	SD	SE
0.1			$n = 10$			$n = 20$			$n = 50$	
	NAIVE	-0.007	0.198	0.115	-0.029	0.194	0.110	-0.049	0.142	0.072
	LIKELIHOOD	-0.012	0.182	0.131	-0.013	0.128	0.106	-0.004	0.064	0.058
	SKEW-NORMAL	-0.009	0.179	0.130	-0.013	0.130	0.106	-0.002	0.062	0.057
	CORRECTED SCORE	0.007	0.168	0.126	0.003	0.129	0.103	0.003	0.082	0.071
	CONDITIONAL SCORE	0.007	0.168	0.126	0.004	0.129	0.103	0.003	0.082	0.071
	SIMEX	-0.002	0.166	0.132	-0.017	0.128	0.108	-0.016	0.079	0.073
0.5			$n = 10$			$n = 20$			$n = 50$	
	NAIVE	0.000	0.537	0.411	-0.033	0.284	0.242	-0.047	0.406	0.196
	LIKELIHOOD	-0.002	0.273	0.229	-0.019	0.199	0.175	-0.005	0.114	0.113
	SKEW-NORMAL	-0.008	0.273	0.228	-0.017	0.199	0.173	-0.002	0.113	0.112
	CORRECTED SCORE	0.016	0.269	0.227	0.000	0.197	0.172	0.016	0.115	0.115
	CONDITIONAL SCORE	0.016	0.268	0.227	0.000	0.197	0.172	0.016	0.116	0.115
	SIMEX	0.002	0.271	0.239	-0.027	0.198	0.180	-0.011	0.117	0.118
0.9			$n = 10$			$n = 20$			$n = 50$	
	NAIVE	-0.059	0.576	0.599	-0.032	0.448	0.432	-0.027	0.297	0.252
	LIKELIHOOD	-0.027	0.352	0.296	0.007	0.237	0.221	-0.008	0.151	0.144
	SKEW-NORMAL	-0.027	0.350	0.296	0.007	0.235	0.221	-0.006	0.151	0.143
	CORRECTED SCORE	-0.004	0.341	0.296	0.029	0.235	0.217	0.017	0.150	0.143
	CONDITIONAL SCORE	-0.005	0.341	0.296	0.029	0.235	0.217	0.017	0.150	0.143
	SIMEX	-0.021	0.345	0.313	-0.006	0.242	0.228	-0.014	0.152	0.148
1.5			$n = 10$			$n = 20$			$n = 50$	
	NAIVE	-0.022	0.717	0.943	-0.040	0.582	0.673	-0.013	0.371	0.379
	LIKELIHOOD	-0.008	0.408	0.340	0.002	0.296	0.277	0.005	0.187	0.179
	SKEW-NORMAL	-0.012	0.421	0.340	0.003	0.297	0.276	0.006	0.187	0.179
	CORRECTED SCORE	0.003	0.409	0.344	0.024	0.293	0.272	0.030	0.185	0.176
	CONDITIONAL SCORE	0.002	0.409	0.344	0.024	0.293	0.272	0.030	0.185	0.176
	SIMEX	-0.025	0.435	0.369	-0.020	0.304	0.288	-0.012	0.190	0.183

Table S11: Bias and standard deviation (SD) of the estimates of β_0, and average of the estimated standard errors (SE) obtained from uncorrected approach (NAIVE), likelihood analysis under a Normal or a Skew-Normal specification of the distribution of ξ, corrected score, conditional score, SIMEX, on the basis of $1,000$ replicates of simulation scenario *ii*). Underlying risk distributed as a mixture of Normals.

τ^2	Method	Bias	SD	SE	Bias	SD	SE	Bias	SD	SE
0.1			$n = 10$			$n = 20$			$n = 50$	
	NAIVE	-0.007	0.180	0.123	-0.069	0.361	0.172	-0.052	0.148	0.084
	LIKELIHOOD	-0.023	0.164	0.123	-0.031	0.139	0.114	-0.006	0.070	0.060
	SKEW-NORMAL	-0.024	0.164	0.122	-0.025	0.197	0.138	0.002	0.129	0.089
	CORRECTED SCORE	-0.010	0.159	0.119	-0.018	0.136	0.111	-0.009	0.087	0.077
	CONDITIONAL SCORE	-0.009	0.159	0.120	-0.018	0.137	0.111	-0.009	0.087	0.077
	SIMEX	-0.014	0.158	0.123	-0.036	0.133	0.114	-0.027	0.082	0.078
0.5			$n = 10$			$n = 20$			$n = 50$	
	NAIVE	-0.022	0.289	0.281	-0.055	0.263	0.250	-0.034	0.215	0.161
	LIKELIHOOD	-0.023	0.255	0.218	-0.031	0.194	0.176	-0.016	0.123	0.117
	SKEW-NORMAL	-0.023	0.255	0.214	-0.029	0.192	0.171	-0.012	0.128	0.110
	CORRECTED SCORE	-0.006	0.251	0.215	-0.012	0.194	0.174	0.005	0.123	0.115
	CONDITIONAL SCORE	-0.006	0.251	0.215	-0.012	0.194	0.174	0.005	0.123	0.115
	SIMEX	-0.013	0.252	0.224	-0.038	0.195	0.182	-0.019	0.123	0.118
0.9			$n = 10$			$n = 20$			$n = 50$	
	NAIVE	-0.039	0.763	0.799	-0.048	0.342	0.378	-0.046	0.229	0.244
	LIKELIHOOD	-0.013	0.328	0.280	-0.020	0.238	0.219	-0.010	0.151	0.143
	SKEW-NORMAL	-0.014	0.325	0.279	-0.018	0.242	0.216	-0.007	0.151	0.141
	CORRECTED SCORE	0.006	0.324	0.279	-0.001	0.235	0.217	0.014	0.150	0.144
	CONDITIONAL SCORE	0.006	0.324	0.280	-0.001	0.235	0.217	0.014	0.150	0.144
	SIMEX	-0.005	0.328	0.292	-0.033	0.240	0.228	-0.017	0.153	0.149
1.5			$n = 10$			$n = 20$			$n = 50$	
	NAIVE	-0.013	0.625	0.894	-0.023	0.777	0.837	-0.051	0.493	0.421
	LIKELIHOOD	-0.014	0.417	0.351	0.010	0.294	0.274	-0.001	0.189	0.177
	SKEW-NORMAL	-0.015	0.413	0.352	0.011	0.291	0.269	0.004	0.185	0.175
	CORRECTED SCORE	0.008	0.410	0.352	0.028	0.294	0.272	0.023	0.187	0.177
	CONDITIONAL SCORE	0.009	0.410	0.353	0.027	0.293	0.272	0.023	0.187	0.177
	SIMEX	-0.004	0.416	0.369	-0.015	0.302	0.287	-0.018	0.192	0.184

Table S12: Bias and standard deviation (SD) of the estimates of β_0, and average of the estimated standard errors (SE) obtained from uncorrected approach (NAIVE), likelihood analysis under a Normal or a Skew-Normal specification of the distribution of ξ, corrected score, conditional score, SIMEX, on the basis of $1,000$ replicates of simulation scenario *ii*). Underlying risk distributed as a Skew-Normal.

τ^2	Method	Bias	SD	SE	Bias	SD	SE	Bias	SD	SE
0.1			$n = 10$			$n = 20$			$n = 50$	
	NAIVE	0.022	0.283	0.121	0.029	0.244	0.091	-0.006	0.237	0.076
	LIKELIHOOD	0.014	0.239	0.149	0.032	0.195	0.129	0.019	0.118	0.075
	SKEW-NORMAL	0.005	0.234	0.149	0.028	0.219	0.143	0.020	0.118	0.075
	CORRECTED SCORE	0.016	0.238	0.146	0.046	0.201	0.123	0.059	0.178	0.099
	CONDITIONAL SCORE	0.017	0.238	0.147	0.045	0.202	0.124	0.058	0.178	0.100
	SIMEX	0.012	0.237	0.146	0.035	0.203	0.129	0.043	0.177	0.114
0.5			$n = 10$			$n = 20$			$n = 50$	
	NAIVE	-0.012	0.509	0.322	0.005	0.489	0.286	0.004	0.282	0.159
	LIKELIHOOD	-0.001	0.355	0.268	0.015	0.266	0.208	0.029	0.182	0.148
	SKEW-NORMAL	-0.015	0.343	0.261	0.014	0.267	0.207	0.035	0.180	0.145
	CORRECTED SCORE	0.006	0.341	0.270	0.030	0.267	0.208	0.069	0.196	0.143
	CONDITIONAL SCORE	0.007	0.341	0.271	0.031	0.267	0.208	0.069	0.196	0.144
	SIMEX	-0.003	0.353	0.276	0.016	0.269	0.213	0.047	0.197	0.156
0.9			$n = 10$			$n = 20$			$n = 50$	
	NAIVE	-0.007	0.629	0.524	0.017	0.468	0.382	0.019	0.398	0.255
	LIKELIHOOD	0.018	0.458	0.350	0.028	0.312	0.260	0.042	0.206	0.180
	SKEW-NORMAL	0.008	0.452	0.343	0.027	0.310	0.257	0.054	0.212	0.179
	CORRECTED SCORE	0.020	0.452	0.354	0.049	0.315	0.260	0.081	0.221	0.175
	CONDITIONAL SCORE	0.020	0.452	0.355	0.049	0.315	0.260	0.081	0.221	0.176
	SIMEX	0.012	0.458	0.360	0.030	0.320	0.266	0.054	0.226	0.187
1.5			$n = 10$			$n = 20$			$n = 50$	
	NAIVE	0.061	0.824	0.872	-0.003	0.655	0.659	-0.004	0.695	0.466
	LIKELIHOOD	0.026	0.552	0.431	0.039	0.375	0.332	0.057	0.251	0.219
	SKEW-NORMAL	0.022	0.545	0.430	0.045	0.372	0.326	0.072	0.249	0.219
	CORRECTED SCORE	0.031	0.541	0.438	0.056	0.373	0.329	0.095	0.256	0.213
	CONDITIONAL SCORE	0.031	0.541	0.439	0.056	0.373	0.330	0.096	0.256	0.214
	SIMEX	0.019	0.559	0.449	0.028	0.383	0.339	0.058	0.263	0.227

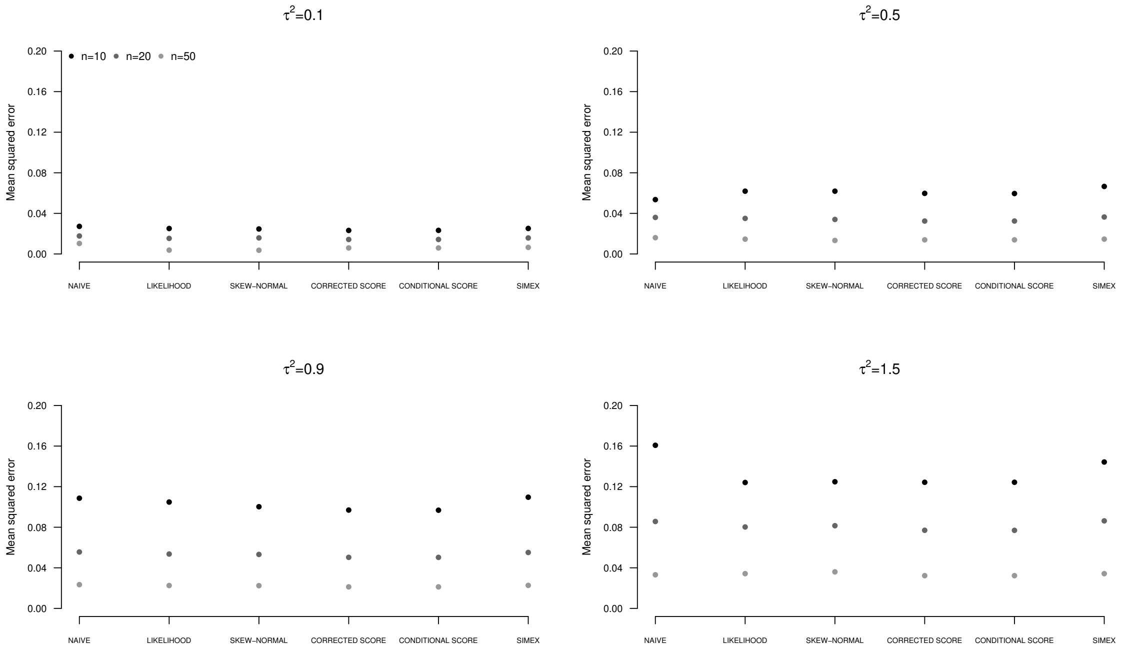

Figure S18: Mean squared error of the estimators β_0 obtained from naive analysis, likelihood analysis under a Normal or a Skew-Normal specification of the distribution of ξ, corrected score, conditional score, SIMEX, on the basis of $1,000$ replicates of simulation scenario i). Underlying risk normally distributed.

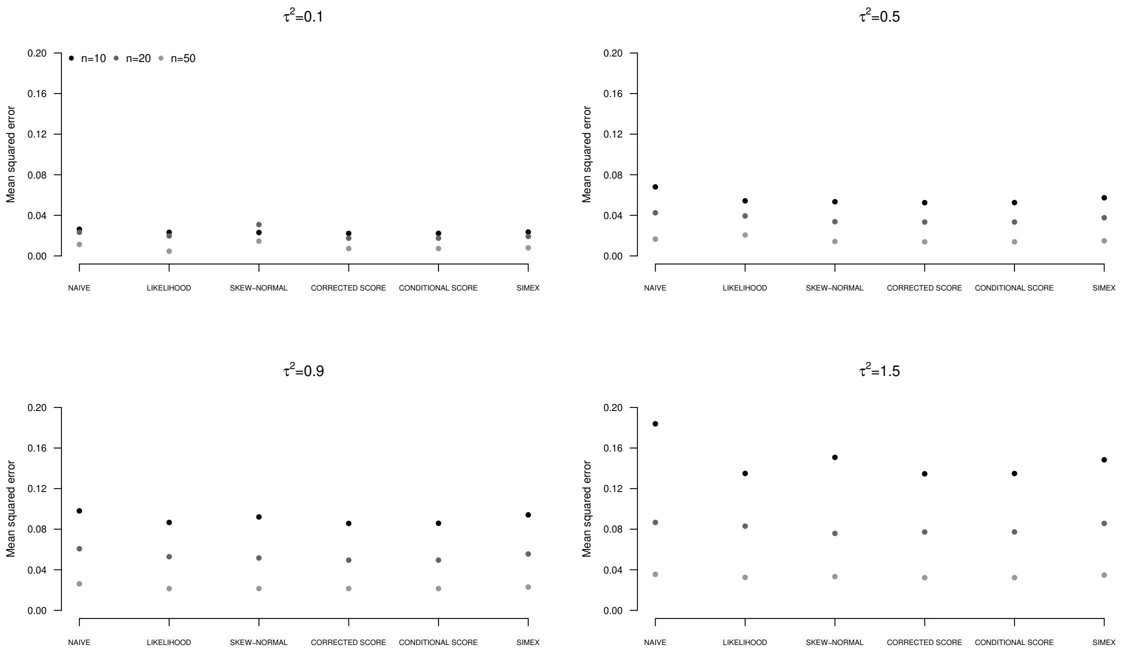

Figure S19: Mean squared error of the estimators β_0 obtained from naive analysis, likelihood analysis under a Normal or a Skew-Normal specification of the distribution of ξ, corrected score, conditional score, SIMEX, on the basis of $1,000$ replicates of simulation scenario i). Underlying risk distributed as a mixture of Normals.

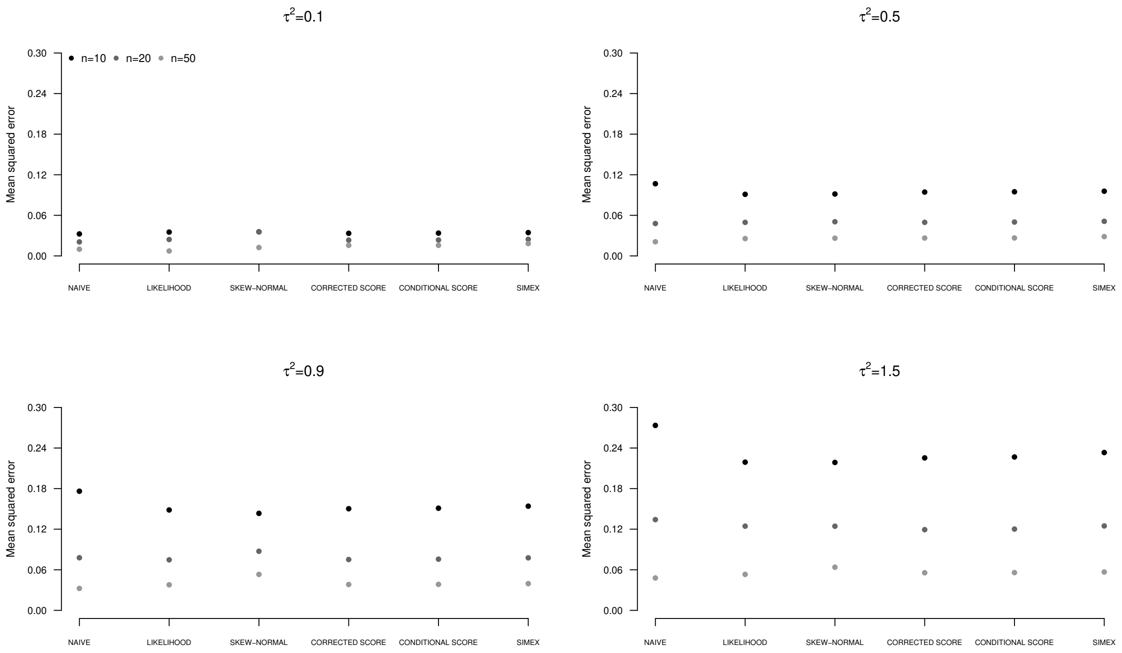

Figure S20: Mean squared error of the estimators β_0 obtained from naive analysis, likelihood analysis under a Normal or a Skew-Normal specification of the distribution of ξ, corrected score, conditional score, SIMEX, on the basis of $1,000$ replicates of simulation scenario i). Underlying risk distributed as a Skew-Normal.

Table S13: Bias and standard deviation (SD) of the estimates of β_1, and average of the estimated standard errors (SE) obtained from uncorrected approach (NAIVE), likelihood analysis under a Normal or a Skew-Normal specification of the distribution of ξ, corrected score, conditional score, SIMEX, on the basis of $1,000$ replicates of simulation scenario ii). Underlying risk normally distributed.

τ^2	Method	Bias	SD	SE	Bias	SD	SE	Bias	SD	SE
0.1			$n = 10$			$n = 20$			$n = 50$	
	NAIVE	-0.055	0.228	0.115	-0.053	0.219	0.110	-0.062	0.191	0.072
	LIKELIHOOD	-0.038	0.230	0.129	-0.051	0.186	0.105	-0.024	0.085	0.061
	SKEW-NORMAL	-0.033	0.225	0.129	-0.050	0.183	0.105	-0.025	0.087	0.061
	CORRECTED SCORE	-0.046	0.223	0.115	-0.061	0.193	0.100	-0.080	0.157	0.087
	CONDITIONAL SCORE	-0.048	0.222	0.116	-0.062	0.193	0.101	-0.081	0.157	0.087
	SIMEX	-0.043	0.225	0.122	-0.047	0.199	0.109	-0.067	0.159	0.093
0.5			$n = 10$			$n = 20$			$n = 50$	
	NAIVE	-0.056	0.466	0.411	-0.069	0.285	0.242	-0.056	0.342	0.196
	LIKELIHOOD	-0.029	0.324	0.231	-0.067	0.249	0.179	-0.039	0.153	0.122
	SKEW-NORMAL	-0.018	0.316	0.232	-0.068	0.248	0.181	-0.044	0.160	0.124
	CORRECTED SCORE	-0.045	0.313	0.212	-0.082	0.247	0.168	-0.088	0.182	0.129
	CONDITIONAL SCORE	-0.047	0.312	0.213	-0.084	0.247	0.169	-0.088	0.181	0.130
	SIMEX	-0.044	0.315	0.226	-0.064	0.253	0.178	-0.067	0.186	0.135
0.9			$n = 10$			$n = 20$			$n = 50$	
	NAIVE	-0.050	0.581	0.599	-0.034	0.483	0.432	-0.097	0.326	0.252
	LIKELIHOOD	-0.058	0.426	0.299	-0.066	0.304	0.226	-0.057	0.190	0.154
	SKEW-NORMAL	-0.052	0.418	0.300	-0.064	0.299	0.227	-0.066	0.197	0.157
	CORRECTED SCORE	-0.060	0.405	0.279	-0.074	0.297	0.210	-0.096	0.207	0.156
	CONDITIONAL SCORE	-0.063	0.404	0.280	-0.076	0.296	0.211	-0.097	0.207	0.156
	SIMEX	-0.056	0.410	0.298	-0.050	0.305	0.222	-0.074	0.211	0.162
1.5			$n = 10$			$n = 20$			$n = 50$	
	NAIVE	-0.058	0.714	0.943	-0.039	0.601	0.673	-0.081	0.359	0.379
	LIKELIHOOD	0.025	0.485	0.380	-0.037	0.356	0.282	-0.071	0.224	0.188
	SKEW-NORMAL	0.011	0.489	0.385	-0.036	0.354	0.283	-0.077	0.225	0.190
	CORRECTED SCORE	0.011	0.488	0.362	-0.046	0.342	0.263	-0.099	0.223	0.184
	CONDITIONAL SCORE	0.009	0.487	0.365	-0.048	0.341	0.264	-0.100	0.223	0.184
	SIMEX	0.031	0.498	0.390	-0.019	0.353	0.279	-0.069	0.230	0.193

Table S14: Bias and standard deviation (SD) of the estimates of β_1, and average of the estimated standard errors (SE) obtained from uncorrected approach (NAIVE), likelihood analysis under a Normal or a Skew-Normal specification of the distribution of ξ, corrected score, conditional score, SIMEX, on the basis of $1,000$ replicates of simulation scenario *ii*). Underlying risk distributed as a mixture of Normals.

τ^2	Method	Bias	SD	SE	Bias	SD	SE	Bias	SD	SE
0.1			$n = 10$			$n = 20$			$n = 50$	
	NAIVE	-0.079	0.309	0.123	-0.079	0.414	0.172	-0.133	0.259	0.084
	LIKELIHOOD	-0.047	0.299	0.166	-0.092	0.254	0.148	-0.043	0.142	0.090
	SKEW-NORMAL	-0.031	0.278	0.168	-0.088	0.331	0.185	-0.057	0.223	0.132
	CORRECTED SCORE	-0.047	0.290	0.154	-0.099	0.255	0.143	-0.158	0.240	0.129
	CONDITIONAL SCORE	-0.054	0.287	0.154	-0.101	0.255	0.143	-0.159	0.237	0.129
	SIMEX	-0.063	0.284	0.154	-0.088	0.260	0.151	-0.143	0.240	0.136
0.5			$n = 10$			$n = 20$			$n = 50$	
	NAIVE	-0.062	0.480	0.281	-0.106	0.380	0.250	-0.126	0.336	0.161
	LIKELIHOOD	-0.016	0.471	0.307	-0.080	0.323	0.244	-0.065	0.225	0.173
	SKEW-NORMAL	-0.013	0.457	0.304	-0.066	0.314	0.237	-0.078	0.224	0.167
	CORRECTED SCORE	-0.037	0.445	0.285	-0.105	0.317	0.233	-0.148	0.271	0.180
	CONDITIONAL SCORE	-0.042	0.442	0.286	-0.107	0.316	0.234	-0.149	0.270	0.180
	SIMEX	-0.045	0.451	0.295	-0.087	0.325	0.248	-0.127	0.277	0.188
0.9			$n = 10$			$n = 20$			$n = 50$	
	NAIVE	-0.044	0.959	0.799	-0.114	0.490	0.378	-0.134	0.369	0.244
	LIKELIHOOD	-0.009	0.551	0.390	-0.089	0.398	0.301	-0.082	0.274	0.216
	SKEW-NORMAL	0.003	0.555	0.390	-0.077	0.387	0.300	-0.095	0.271	0.211
	CORRECTED SCORE	-0.030	0.533	0.363	-0.109	0.398	0.287	-0.154	0.296	0.215
	CONDITIONAL SCORE	-0.036	0.530	0.363	-0.112	0.396	0.287	-0.156	0.295	0.216
	SIMEX	-0.035	0.542	0.378	-0.087	0.410	0.305	-0.128	0.303	0.227
1.5			$n = 10$			$n = 20$			$n = 50$	
	NAIVE	-0.020	1.019	0.894	0.012	0.956	0.837	-0.083	0.605	0.421
	LIKELIHOOD	0.004	0.733	0.500	-0.066	0.469	0.381	-0.067	0.306	0.264
	SKEW-NORMAL	0.003	0.723	0.498	-0.058	0.464	0.374	-0.081	0.309	0.256
	CORRECTED SCORE	-0.034	0.674	0.474	-0.081	0.459	0.363	-0.136	0.320	0.256
	CONDITIONAL SCORE	-0.039	0.669	0.473	-0.084	0.458	0.364	-0.138	0.319	0.257
	SIMEX	-0.026	0.695	0.494	-0.052	0.472	0.387	-0.103	0.332	0.271

Table S15: Bias and standard deviation (SD) of the estimates of β_1, and average of the estimated standard errors (SE) obtained from uncorrected approach (NAIVE, likelihood analysis under a Normal or a Skew-Normal specification of the distribution of ξ, corrected score, conditional score, SIMEX, on the basis of 1,000 replicates of simulation scenario *ii*). Underlying risk distributed as a Skew-Normal.

τ^2	Method	Bias	SD	SE	Bias	SD	SE	Bias	SD	SE
0.1			$n = 10$			$n = 20$			$n = 50$	
	NAIVE	-0.058	0.271	0.121	-0.079	0.258	0.091	-0.066	0.230	0.076
	LIKELIHOOD	-0.050	0.255	0.140	-0.073	0.235	0.126	-0.042	0.134	0.074
	SKEW-NORMAL	-0.038	0.242	0.141	-0.061	0.261	0.139	-0.040	0.141	0.076
	CORRECTED SCORE	-0.038	0.246	0.119	-0.076	0.240	0.107	-0.112	0.216	0.105
	CONDITIONAL SCORE	-0.040	0.246	0.120	-0.075	0.242	0.109	-0.109	0.217	0.106
	SIMEX	-0.038	0.245	0.132	-0.071	0.244	0.120	-0.103	0.219	0.11
0.5			$n = 10$			$n = 20$			$n = 50$	
	NAIVE	-0.053	0.487	0.322	-0.048	0.456	0.286	-0.069	0.281	0.159
	LIKELIHOOD	-0.043	0.415	0.262	-0.063	0.312	0.212	-0.074	0.213	0.152
	SKEW-NORMAL	-0.030	0.398	0.258	-0.062	0.317	0.211	-0.080	0.217	0.151
	CORRECTED SCORE	-0.034	0.384	0.242	-0.063	0.295	0.191	-0.115	0.230	0.150
	CONDITIONAL SCORE	-0.035	0.384	0.242	-0.063	0.295	0.192	-0.115	0.229	0.151
	SIMEX	-0.039	0.391	0.261	-0.056	0.297	0.204	-0.105	0.233	0.160
0.9			$n = 10$			$n = 20$			$n = 50$	
	NAIVE	-0.008	0.623	0.524	-0.090	0.477	0.382	-0.084	0.362	0.255
	LIKELIHOOD	-0.029	0.496	0.346	-0.074	0.346	0.257	-0.087	0.241	0.185
	SKEW-NORMAL	-0.016	0.490	0.340	-0.076	0.341	0.255	-0.104	0.250	0.185
	CORRECTED SCORE	-0.026	0.475	0.315	-0.086	0.336	0.237	-0.128	0.255	0.180
	CONDITIONAL SCORE	-0.027	0.474	0.315	-0.087	0.336	0.237	-0.127	0.255	0.181
	SIMEX	-0.023	0.478	0.340	-0.078	0.342	0.249	-0.114	0.260	0.190
1.5			$n = 10$			$n = 20$			$n = 50$	
	NAIVE	-0.057	0.879	0.872	-0.050	0.655	0.659	-0.050	0.628	0.466
	LIKELIHOOD	-0.032	0.614	0.436	-0.083	0.421	0.329	-0.098	0.282	0.225
	SKEW-NORMAL	-0.029	0.606	0.441	-0.087	0.411	0.329	-0.120	0.287	0.226
	CORRECTED SCORE	-0.039	0.570	0.395	-0.088	0.404	0.304	-0.131	0.289	0.217
	CONDITIONAL SCORE	-0.040	0.570	0.395	-0.089	0.403	0.304	-0.131	0.288	0.217
	SIMEX	-0.026	0.598	0.430	-0.074	0.412	0.320	-0.112	0.294	0.226

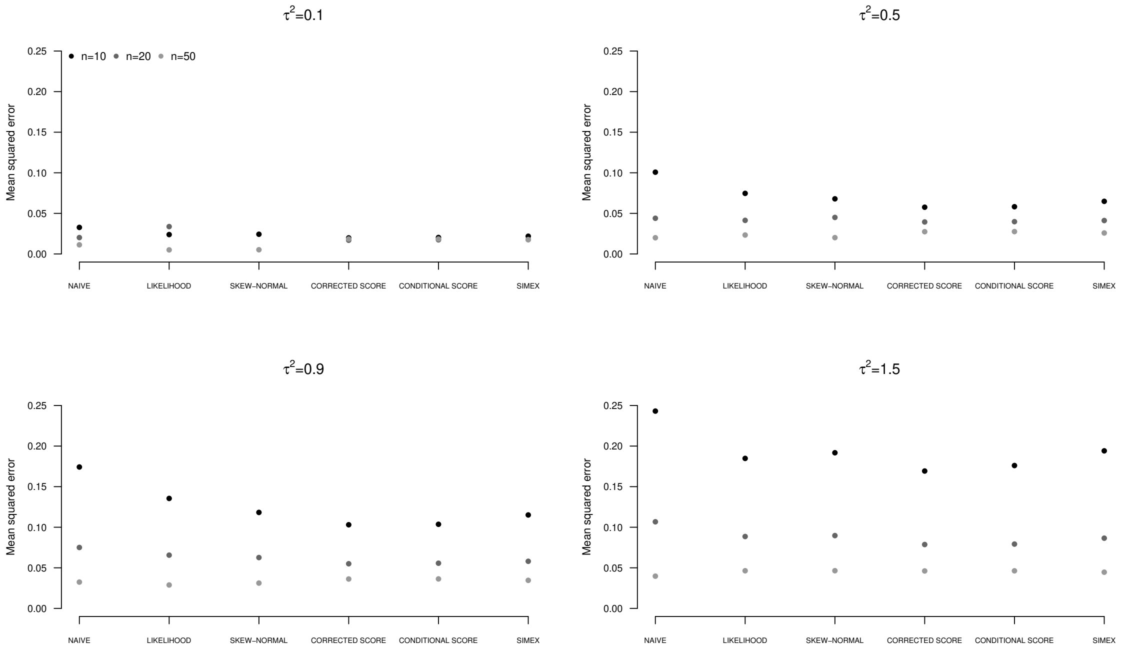

Figure S21: Mean squared error of the estimators β_1 obtained from naive analysis, likelihood analysis under a Normal or a Skew-Normal specification of the distribution of ξ, corrected score, conditional score, SIMEX, on the basis of $1,000$ replicates of simulation scenario i). Underlying risk normally distributed.

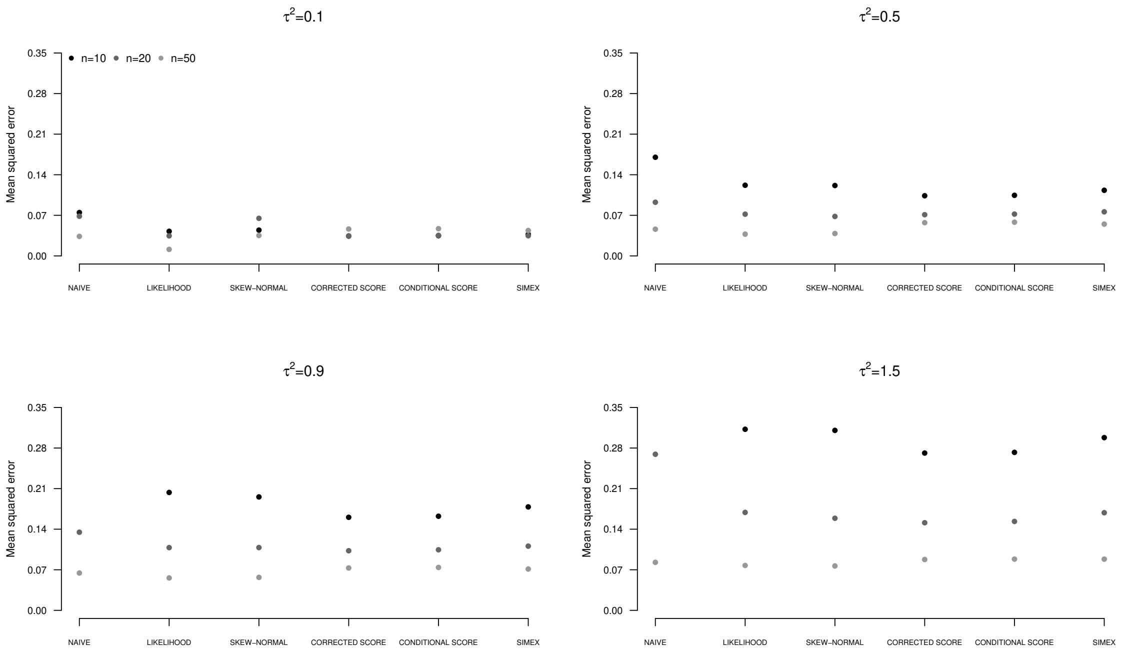

Figure S22: Mean squared error of the estimators β_1 obtained from naive analysis, likelihood analysis under a Normal or a Skew-Normal specification of the distribution of ξ, corrected score, conditional score, SIMEX, on the basis of $1,000$ replicates of simulation scenario i). Underlying risk distributed as a mixture of Normals.

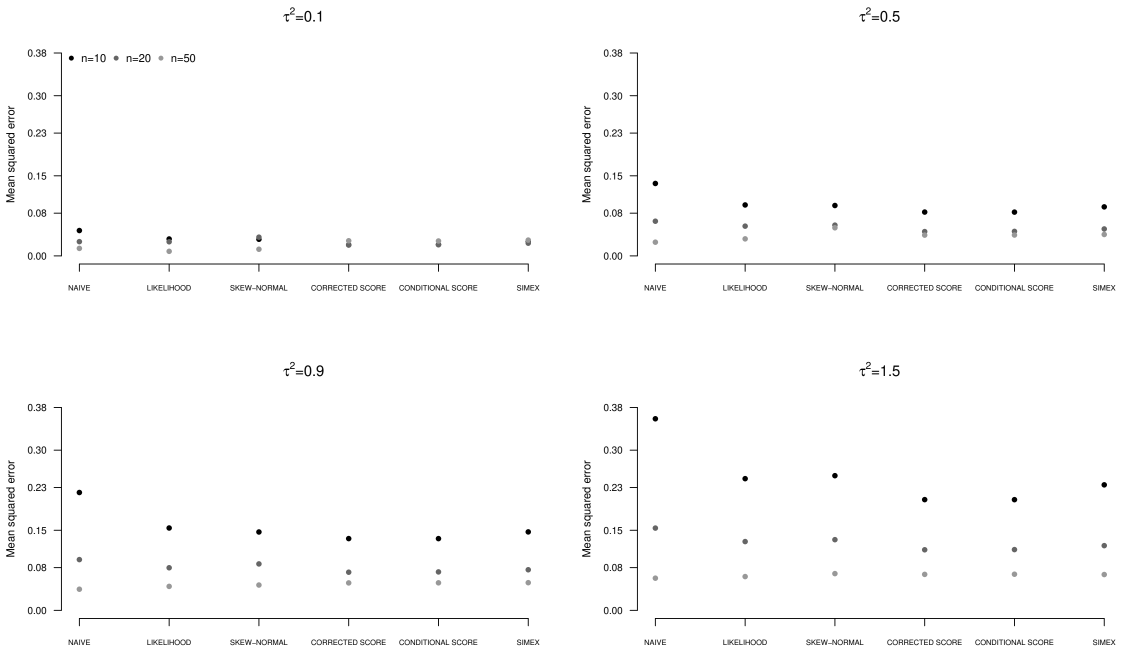

Figure S23: Mean squared error of the estimators β_1 obtained from naive analysis, likelihood analysis under a Normal or a Skew-Normal specification of the distribution of ξ, corrected score, conditional score, SIMEX, on the basis of $1,000$ replicates of simulation scenario i). Underlying risk distributed as a Skew-Normal.

Table S16: Bias and standard deviation (SD) of the estimates of τ^2, and average of the estimated standard errors (SE) obtained from uncorrected approach (NAIVE, likelihood analysis under a Normal or a Skew-Normal specification of the distribution of ξ, corrected score, conditional score, SIMEX, on the basis of 1,000 replicates of simulation scenario *ii*). Underlying risk normally distributed.

τ^2	Method	Bias	SD	SE	Bias	SD	SE	Bias	SD	SE
0.1			$n=10$			$n=20$			$n=50$	
	NAIVE	0.131	0.497	0.115	0.230	0.452	0.110	0.252	0.298	0.072
	LIKELIHOOD	0.017	0.148	0.048	0.055	0.189	0.092	0.037	0.156	0.053
	SKEW-NORMAL	0.012	0.137	0.044	0.047	0.176	0.084	0.029	0.132	0.042
	CORRECTED SCORE	0.081	0.453	0.089	0.139	0.388	0.102	0.164	0.263	0.112
	CONDITIONAL SCORE	0.081	0.453	0.089	0.139	0.388	0.102	0.164	0.263	0.112
	SIMEX	0.129	0.562	0.203	0.176	0.430	0.202	0.186	0.276	0.170
0.5			$n=10$			$n=20$			$n=50$	
	NAIVE	0.323	0.832	0.411	0.227	0.493	0.242	0.461	0.644	0.196
	LIKELIHOOD	-0.075	0.265	0.249	-0.022	0.228	0.238	0.079	0.240	0.281
	SKEW-NORMAL	-0.081	0.259	0.244	-0.025	0.225	0.236	0.075	0.240	0.269
	CORRECTED SCORE	-0.021	0.472	0.136	0.071	0.421	0.131	0.137	0.289	0.113
	CONDITIONAL SCORE	-0.020	0.473	0.136	0.071	0.421	0.131	0.137	0.289	0.113
	SIMEX	0.123	0.596	0.399	0.168	0.475	0.311	0.192	0.302	0.225
0.9			$n=10$			$n=20$			$n=50$	
	NAIVE	0.299	0.962	0.599	0.397	0.674	0.432	0.334	0.434	0.252
	LIKELIHOOD	-0.173	0.364	0.524	-0.076	0.325	0.498	0.041	0.277	0.461
	SKEW-NORMAL	-0.176	0.369	0.526	-0.074	0.326	0.502	0.045	0.282	0.462
	CORRECTED SCORE	-0.088	0.570	0.172	-0.012	0.442	0.151	0.086	0.316	0.118
	CONDITIONAL SCORE	-0.087	0.570	0.172	-0.012	0.442	0.151	0.086	0.316	0.118
	SIMEX	0.160	0.734	0.619	0.145	0.507	0.413	0.177	0.340	0.281
1.5			$n=10$			$n=20$			$n=50$	
	NAIVE	0.387	1.350	0.943	0.520	1.003	0.673	0.355	0.502	0.379
	LIKELIHOOD	-0.522	0.417	0.825	-0.226	0.392	0.903	-0.059	0.338	0.754
	SKEW-NORMAL	-0.529	0.425	0.820	-0.226	0.392	0.907	-0.057	0.338	0.756
	CORRECTED SCORE	-0.369	0.524	0.209	-0.118	0.525	0.182	-0.002	0.389	0.133
	CONDITIONAL SCORE	-0.369	0.523	0.209	-0.118	0.525	0.182	-0.002	0.389	0.133
	SIMEX	0.031	0.705	0.879	0.142	0.612	0.604	0.156	0.417	0.384

Table S17: Bias and standard deviation (SD) of the estimates of τ^2, and average of the estimated standard errors (SE) obtained from uncorrected approach (NAIVE, likelihood analysis under a Normal or a Skew-Normal specification of the distribution of ξ, corrected score, conditional score, SIMEX, on the basis of $1,000$ replicates of simulation scenario *ii*). Underlying risk distributed as a mixture of Normals.

τ^2	Method	Bias	SD	SE	Bias	SD	SE	Bias	SD	SE
0.1			$n=10$			$n=20$			$n=50$	
	NAIVE	0.146	0.622	0.123	0.416	0.846	0.172	0.310	0.337	0.084
	LIKELIHOOD	0.007	0.093	0.038	0.091	0.248	0.154	0.063	0.220	0.085
	SKEW-NORMAL	0.000	0.082	0.035	0.164	0.362	0.209	0.190	0.434	0.171
	CORRECTED SCORE	0.096	0.555	0.090	0.199	0.467	0.123	0.228	0.314	0.132
	CONDITIONAL SCORE	0.096	0.555	0.090	0.201	0.469	0.123	0.228	0.314	0.132
	SIMEX	0.151	0.693	0.233	0.244	0.518	0.266	0.254	0.325	0.216
0.5			$n=10$			$n=20$			$n=50$	
	NAIVE	0.062	0.557	0.281	0.251	0.495	0.250	0.290	0.339	0.161
	LIKELIHOOD	-0.064	0.247	0.251	0.004	0.228	0.268	0.101	0.264	0.307
	SKEW-NORMAL	-0.082	0.252	0.244	-0.023	0.254	0.264	0.056	0.312	0.286
	CORRECTED SCORE	-0.011	0.523	0.136	0.117	0.454	0.143	0.154	0.305	0.116
	CONDITIONAL SCORE	-0.011	0.523	0.136	0.117	0.454	0.142	0.154	0.305	0.115
	SIMEX	0.128	0.657	0.401	0.220	0.504	0.349	0.211	0.317	0.232
0.9			$n=10$			$n=20$			$n=50$	
	NAIVE	0.698	2.221	0.799	0.235	0.565	0.378	0.295	0.368	0.244
	LIKELIHOOD	-0.171	0.356	0.527	-0.065	0.318	0.515	0.084	0.281	0.508
	SKEW-NORMAL	-0.177	0.366	0.528	-0.096	0.322	0.496	0.041	0.323	0.490
	CORRECTED SCORE	-0.095	0.597	0.175	0.033	0.483	0.160	0.120	0.326	0.122
	CONDITIONAL SCORE	-0.095	0.597	0.175	0.033	0.483	0.160	0.120	0.326	0.122
	SIMEX	0.140	0.763	0.624	0.193	0.543	0.451	0.220	0.344	0.295
1.5			$n=10$			$n=20$			$n=50$	
	NAIVE	0.288	1.351	0.894	1.011	1.421	0.837	0.562	0.703	0.421
	LIKELIHOOD	-0.459	0.449	0.822	-0.180	0.396	0.939	-0.009	0.336	0.793
	SKEW-NORMAL	-0.479	0.450	0.809	-0.232	0.422	0.893	-0.080	0.385	0.753
	CORRECTED SCORE	-0.222	0.777	0.211	-0.041	0.576	0.186	0.034	0.372	0.133
	CONDITIONAL SCORE	-0.222	0.777	0.211	-0.041	0.576	0.186	0.034	0.372	0.133
	SIMEX	0.144	0.994	0.926	0.231	0.677	0.633	0.203	0.413	0.389

Table S18: Bias and standard deviation (SD) of the estimates of τ^2, and average of the estimated standard errors (SE) obtained from uncorrected approach (NAIVE, likelihood analysis under a Normal or a Skew-Normal specification of the distribution of ξ, corrected score, conditional score, SIMEX, on the basis of $1,000$ replicates of simulation scenario *ii*). Underlying risk distributed as a Skew-normal.

τ^2	Method	Bias	SD	SE	Bias	SD	SE	Bias	SD	SE
0.1			$n = 10$			$n = 20$			$n = 50$	
	NAIVE	0.142	0.566	0.121	0.174	0.407	0.091	0.272	0.336	0.076
	LIKELIHOOD	0.010	0.114	0.038	0.055	0.157	0.079	0.048	0.176	0.060
	SKEW-NORMAL	0.006	0.104	0.036	0.089	0.258	0.110	0.047	0.188	0.053
	CORRECTED SCORE	0.077	0.473	0.081	0.118	0.356	0.089	0.189	0.285	0.114
	CONDITIONAL SCORE	0.077	0.473	0.081	0.118	0.356	0.089	0.189	0.285	0.114
	SIMEX	0.126	0.592	0.189	0.151	0.400	0.162	0.211	0.298	0.174
0.5			$n = 10$			$n = 20$			$n = 50$	
	NAIVE	0.145	0.809	0.322	0.358	0.785	0.286	0.281	0.342	0.159
	LIKELIHOOD	-0.094	0.231	0.227	-0.007	0.223	0.246	0.106	0.249	0.301
	SKEW-NORMAL	-0.127	0.231	0.204	-0.016	0.239	0.247	0.090	0.249	0.278
	CORRECTED SCORE	-0.004	0.560	0.137	0.083	0.425	0.130	0.158	0.291	0.114
	CONDITIONAL SCORE	-0.004	0.560	0.137	0.083	0.425	0.130	0.158	0.291	0.114
	SIMEX	0.133	0.706	0.413	0.164	0.474	0.301	0.209	0.308	0.227
0.9			$n = 10$			$n = 20$			$n = 50$	
	NAIVE	0.148	0.818	0.524	0.247	0.623	0.382	0.351	0.469	0.255
	LIKELIHOOD	-0.189	0.359	0.496	-0.077	0.313	0.481	0.077	0.288	0.491
	SKEW-NORMAL	-0.219	0.357	0.471	-0.097	0.313	0.462	0.076	0.288	0.483
	CORRECTED SCORE	-0.114	0.577	0.167	0.018	0.478	0.152	0.127	0.331	0.122
	CONDITIONAL SCORE	-0.114	0.577	0.167	0.018	0.478	0.152	0.127	0.331	0.122
	SIMEX	0.101	0.726	0.577	0.149	0.537	0.415	0.209	0.345	0.292
1.5			$n = 10$			$n = 20$			$n = 50$	
	NAIVE	0.244	1.142	0.872	0.476	0.907	0.659	0.783	1.034	0.466
	LIKELIHOOD	-0.467	0.458	0.817	-0.175	0.415	0.926	-0.008	0.339	0.790
	SKEW-NORMAL	-0.485	0.462	0.802	-0.204	0.430	0.899	-0.007	0.345	0.792
	CORRECTED SCORE	-0.258	0.709	0.206	-0.036	0.590	0.184	0.062	0.392	0.135
	CONDITIONAL SCORE	-0.258	0.709	0.206	-0.036	0.590	0.184	0.062	0.392	0.135
	SIMEX	0.093	0.913	0.870	0.184	0.668	0.612	0.204	0.424	0.395

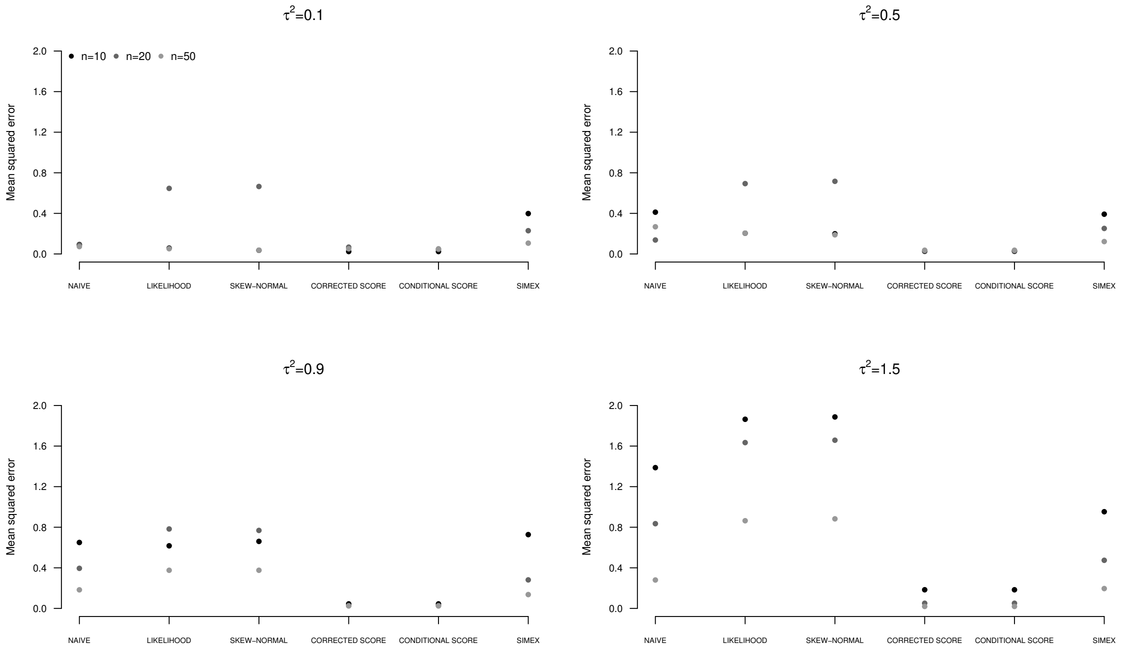

Figure S24: Mean squared error of the estimators τ^2 obtained from naive analysis, likelihood analysis under a Normal or a Skew-Normal specification of the distribution of ξ, corrected score, conditional score, SIMEX, on the basis of $1,000$ replicates of simulation scenario i). Underlying risk normally distributed.

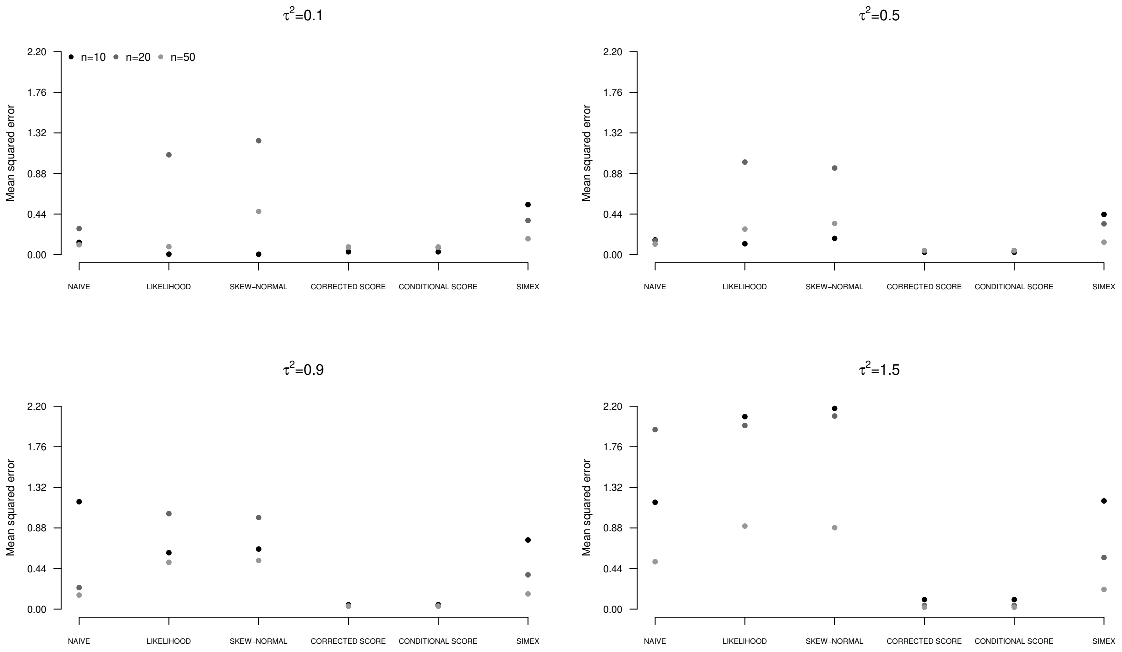

Figure S25: Mean squared error of the estimators τ^2 obtained from naive analysis, likelihood analysis under a Normal or a Skew-Normal specification of the distribution of ξ, corrected score, conditional score, SIMEX, on the basis of $1,000$ replicates of simulation scenario i). Underlying risk distributed as a mixture of Normals.

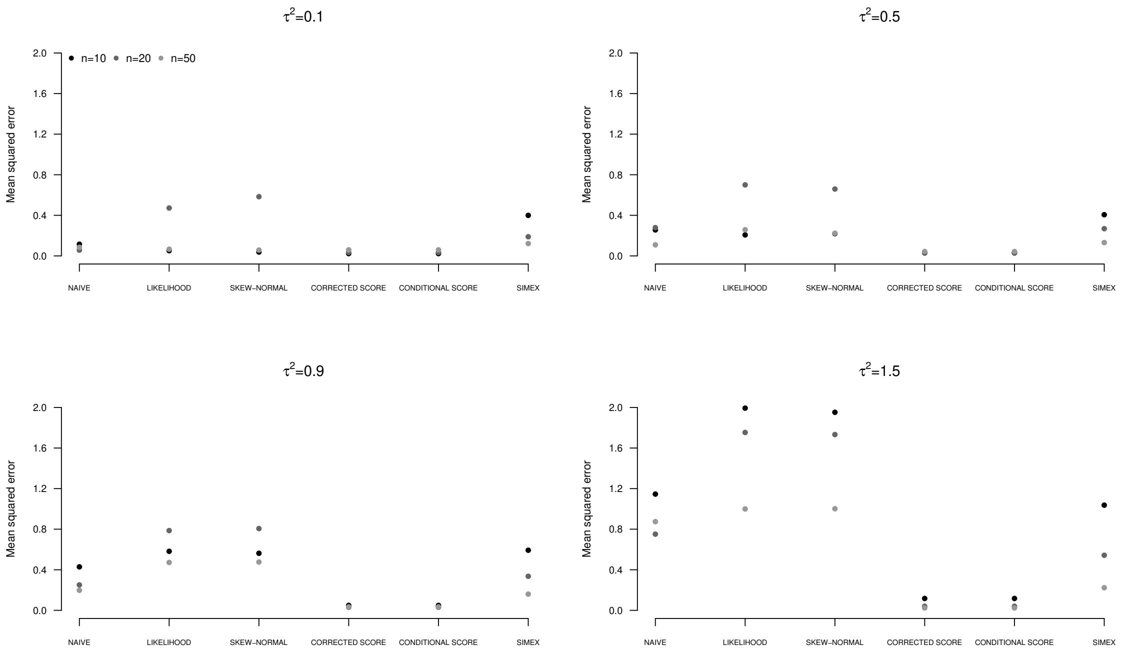

Figure S26: Mean squared error of the estimators τ^2 obtained from naive analysis, likelihood analysis under a Normal or a Skew-Normal specification of the distribution of ξ, corrected score, conditional score, SIMEX, on the basis of $1,000$ replicates of simulation scenario i). Underlying risk distributed as a Skew-Normal.

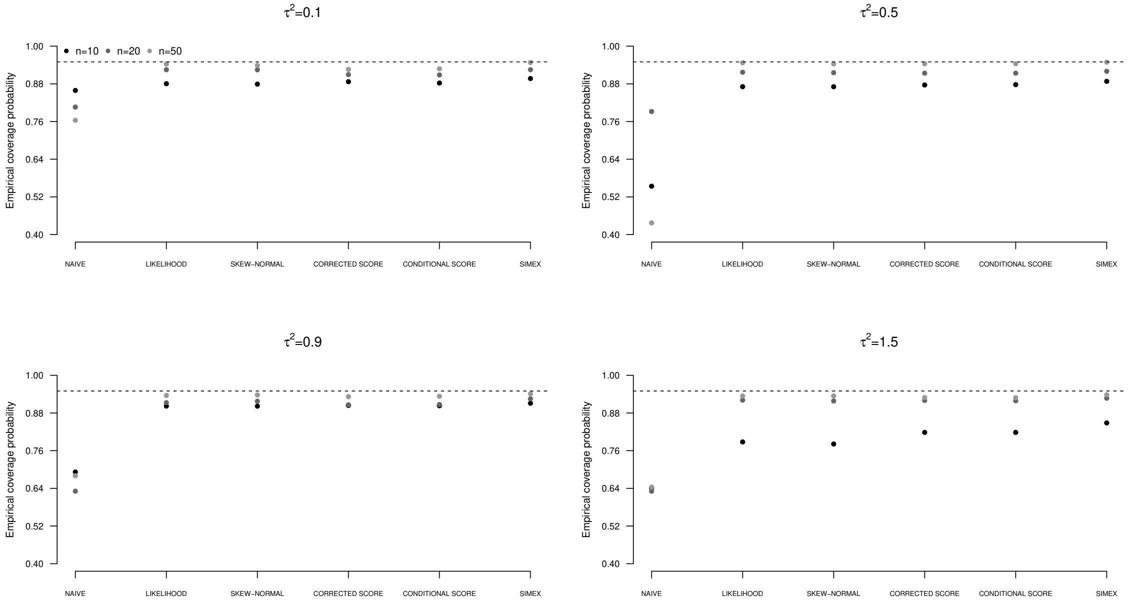

Figure S27: Empirical coverage probabilities of confidence intervals for β_0 from uncorrected approach (NAIVE), likelihood approach under a normal specification (LIKELIHOOD) or a Skew-Normal specification (SKEW-NORMAL) for the underlying risk distribution, SIMEX, corrected score and conditional score, on the basis of $1,000$ replicates of simulation scenario *ii*). Underlying risk normally distributed.

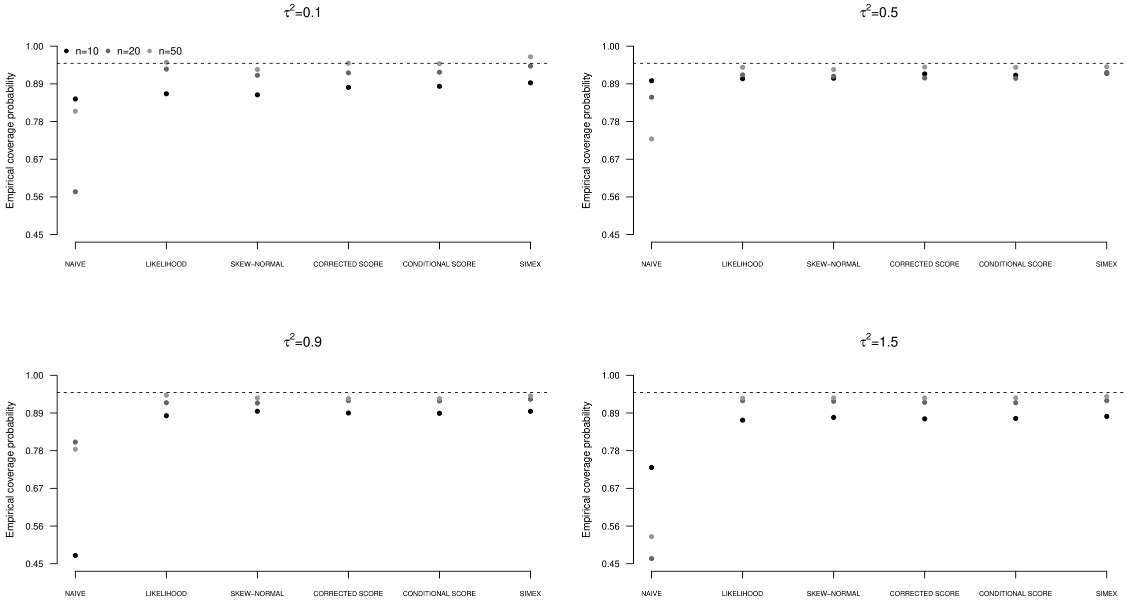

Figure S28: Empirical coverage probabilities of confidence intervals for β_0 from uncorrected approach (NAIVE), likelihood approach under a Normal specification (LIKELIHOOD) or a Skew-Normal specification (SKEW-NORMAL) for the underlying risk distribution, SIMEX, corrected score and conditional score, on the basis of $1,000$ replicates of simulation scenario *ii*). Underlying risk distributed as a mixture of Normals.

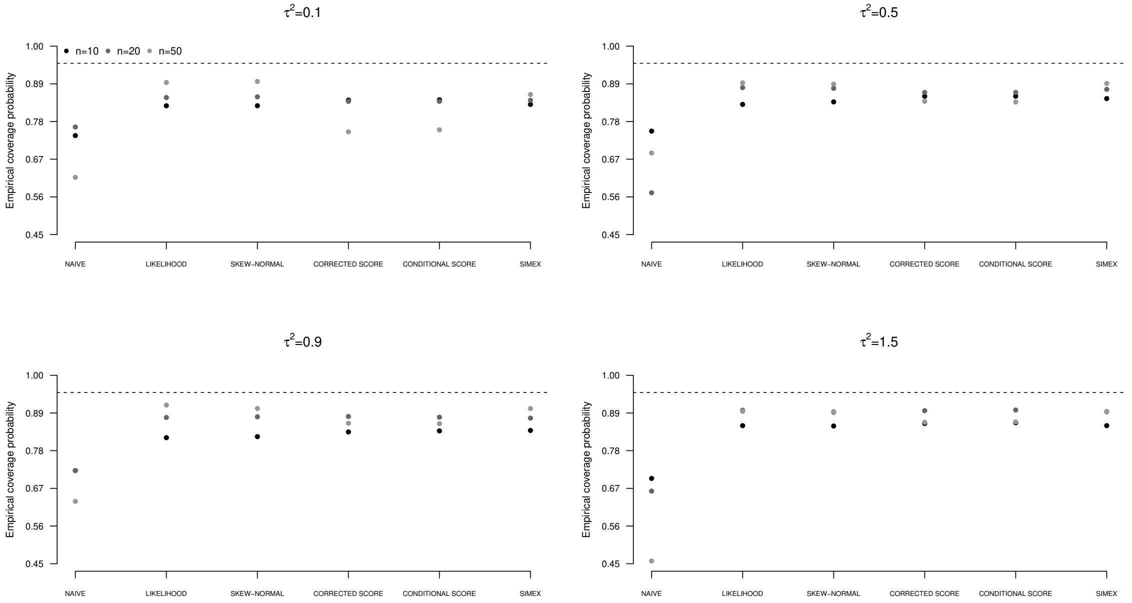

Figure S29: Empirical coverage probabilities of confidence intervals for β_0 from uncorrected approach (NAIVE), likelihood approach under a Normal specification (LIKELIHOOD) or a Skew-Normal specification (SKEW-NORMAL) for the underlying risk distribution, SIMEX, corrected score and conditional score, on the basis of $1,000$ replicates of simulation scenario ii). Underlying risk distributed as a Skew-Normal.

Table S19: Empirical coverage probabilities (multiplied by 1,000) of confidence intervals for β_0 and associated Monte Carlo standard error (in parentheses, multiplied by 1,000) obtained from naive analysis, likelihood analysis under a Normal or a Skew-Normal specification of the distribution of ξ, corrected score, conditional score, SIMEX, on the basis of $1,000$ replicates of simulation scenario *ii*). Underlying risk distributed as a Normal, Skew-Normal, or Mixture of Normals.

		n			n			n		
τ^2	Method	10	20	50	10	20	50	10	20	50
0.1			Normal			Mixture			Skew-Normal	
	NAIVE	859 (11)	806 (13)	764 (13)	846 (11)	575 (16)	810 (12)	739 (14)	764 (13)	617 (15
	LIKELIHOOD	881 (10)	925 (8)	943 (7)	861 (11)	933 (8)	953 (7)	826 (12)	850 (11)	894 (10
	SKEW-NORMAL	879 (11)	925 (9)	939 (8)	858 (12)	915 (9)	932 (8)	826 (12)	852 (11)	897 (10
	CORRECTED SCORE	887 (10)	909 (9)	926 (8)	880 (10)	922 (8)	950 (7)	842 (12)	839 (12)	750 (14
	CONDITIONAL SCORE	883 (10)	908 (9)	928 (8)	883 (10)	924 (8)	949 (7)	843 (12)	839 (12)	756 (14
	SIMEX	897 (10)	925 (8)	948 (7)	893 (10)	942 (7)	969 (5)	830 (12)	842 (12)	859 (11
0.5			Normal			Mixture			Skew-Normal	
	NAIVE	554 (16)	792 (13)	437 (16)	899 (10)	851 (11)	729 (14)	752 (14)	572 (16)	688 (15
	LIKELIHOOD	871 (11)	917 (9)	947 (7)	905 (9)	916 (9)	938 (8)	830 (12)	879 (10)	893 (10
	SKEW-NORMAL	871 (11)	916 (9)	944 (7)	906 (9)	912 (9)	932 (8)	837 (12)	877 (10)	889 (10
	CORRECTED SCORE	876 (10)	914 (9)	944 (7)	919 (9)	907 (9)	939 (8)	854 (11)	865 (11)	839 (12
	CONDITIONAL SCORE	878 (10)	914 (9)	944 (7)	915 (9)			854 (11)	865 (11)	837 (12
	SIMEX	888 (10)	920 (9)	949 (7)	921 (9)	923 (8)	940 (8)	847 (11)	874 (10)	891 (10
0.9			Normal			Mixture			Skew-Normal	
	NAIVE	692 (15)	631 (15)	680 (15)	474 (16)	805 (13)	784 (13)	722 (14)	722 (14)	632 (15
	LIKELIHOOD	902 (9)	913 (9)	936 (8)	882 (10)	920 (9)	942 (7)	818 (12)	877 (10)	913 (9
	SKEW-NORMAL	902 (10)	917 (9)	938 (8)	895 (10)	919 (9)	934 (8)	821 (12)	879 (10)	903 (9
	CORRECTED SCORE	904 (9)	906 (9)	932 (8)	890 (10)	926 (8)	932 (8)	835 (12)	880 (10)	860 (11
	CONDITIONAL SCORE	903 (9)	907 (9)	933 (8)	889 (10)	925 (8)	932 (8)	838 (12)	878 (10)	859 (11
	SIMEX	911 (9)	925 (8)	941 (7)	895 (10)	930 (8)	940 (8)	839 (12)	875 (10)	903 (9
1.5			Normal			Mixture			Skew-Normal	
	NAIVE	641 (15)	631 (15)	644 (15)	731 (14)	465 (16)	529 (16)	699 (15)	662 (15)	458 (16
	LIKELIHOOD	788 (71)	921 (9)	934 (8)	869 (11)	926 (8)	933 (8)	853 (11)	898 (10)	895 (10
	SKEW-NORMAL	781 (73)	918 (9)	934 (8)	877 (10)	924 (8)	934 (8)	852 (11)	892 (10)	894 (10
	CORRECTED SCORE	818 (67)	920 (9)	929 (8)	873 (11)	921 (9)	934 (8)	859 (11)	897 (10)	863 (11

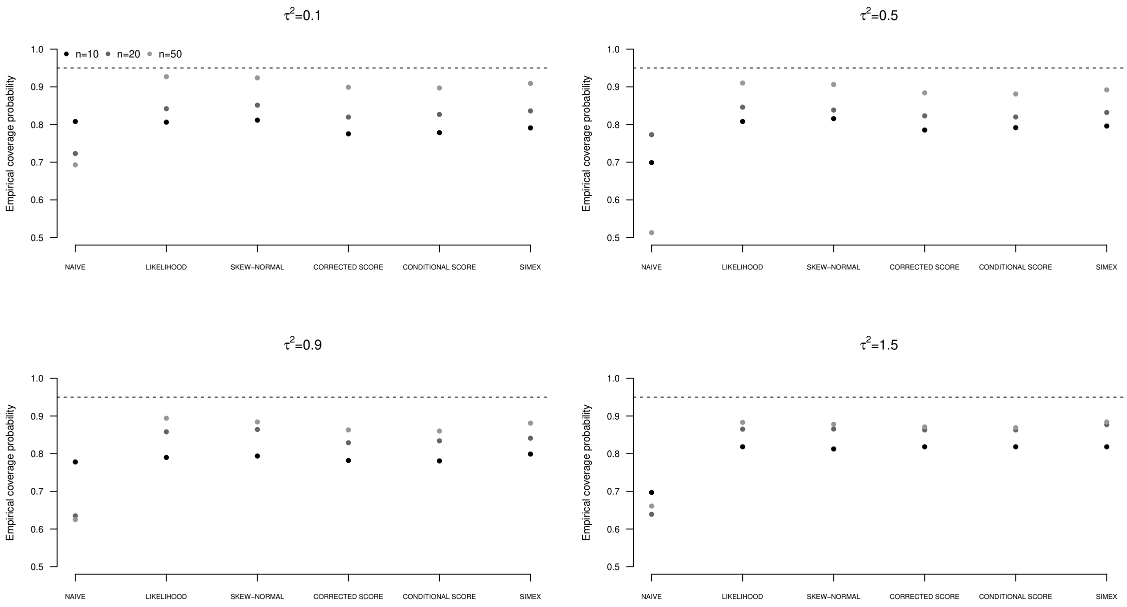

Figure S30: Empirical coverage probabilities of confidence intervals for β_1 from uncorrected approach (NAIVE), likelihood approach under a Normal specification (LIKELIHOOD) or a Skew-Normal specification (SKEW-NORMAL) for the underlying risk distribution, SIMEX, corrected score and conditional score, on the basis of $1,000$ replicates of simulation scenario *ii*). Underlying risk normally distributed.

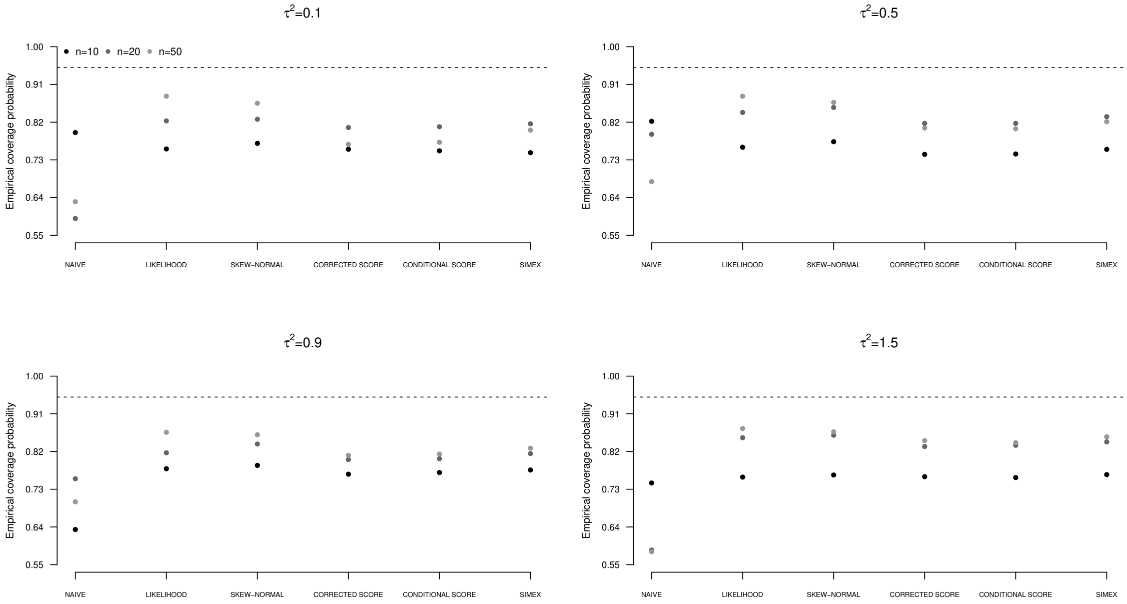

Figure S31: Empirical coverage probabilities of confidence intervals for β_1 from uncorrected approach (NAIVE), likelihood approach under a Normal specification (LIKELIHOOD) or a Skew-Normal specification (SKEW-NORMAL) for the underlying risk distribution, SIMEX, corrected score and conditional score, on the basis of $1,000$ replicates of simulation scenario ii). Underlying risk distributed as a mixture of Normals.

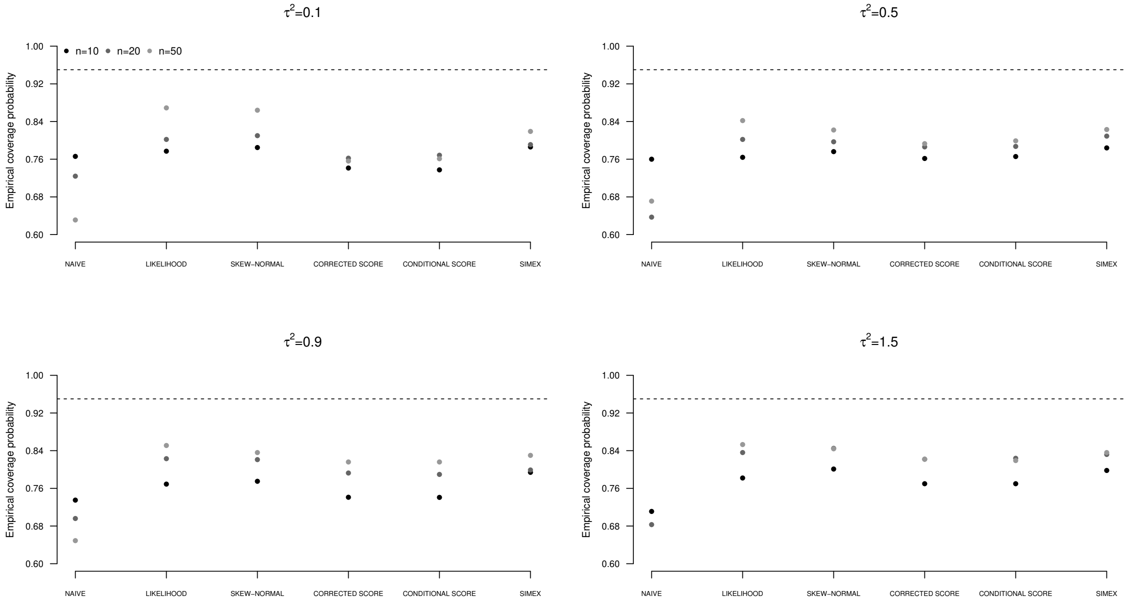

Figure S32: Empirical coverage probabilities of confidence intervals for β_1 from uncorrected approach (NAIVE), likelihood approach under a Normal specification (LIKELIHOOD) or a Skew-Normal specification (SKEW-NORMAL) for the underlying risk distribution, SIMEX, corrected score and conditional score, on the basis of $1,000$ replicates of simulation scenario ii). Underlying risk distributed as a Skew-Normal.

Table S20: Empirical coverage probabilities (multiplied by 1,000) of confidence intervals for β_1 and associated Monte Carlo standard error (in parentheses, multiplied by 1,000) obtained from naive analysis, likelihood analysis under a Normal or a Skew-Normal specification of the distribution of ξ, corrected score, conditional score, SIMEX, on the basis of $1,000$ replicates of simulation scenario *ii*). Underlying risk distributed as a Normal, Skew-Normal, or Mixture of Normals.

			n			n			n	
τ^2	Method	10	20	50	10	20	50	10	20	50
0.1			Normal			Mixture			Skew-Normal	
	NAIVE	808 (12)	723 (14)	693 (15)	795 (13)	590 (16)	630 (15)	766 (13)	724 (14)	631 (15
	LIKELIHOOD	806 (13)	842 (12)	927 (8)	756 (14)	823 (12)	882 (10)	777 (13)	802 (13)	869 (11
	SKEW-NORMAL	812 (13)	851 (12)	924 (8)	769 (14)	827 (12)	865 (11)	785 (14)	810 (12)	864 (11
	CORRECTED SCORE	775 (13)	820 (12)	899 (10)	752 (14)	809 (12)	772 (13)	737 (14)	768 (13)	761 (14
	CONDITIONAL SCORE	778 (13)	827 (12)	897 (10)	752 (14)	809 (12)	772 (13)	737 (14)	768 (13)	761 (14
	SIMEX	791 (13)	836 (12)	909 (9)	747 (14)	816 (12)	801 (13)	786 (13)	791 (13)	819 (12
0.5			Normal			Mixture			Skew-Normal	
	NAIVE	699 (15)	773 (13)	513 (16)	822 (12)	791 (13)	678 (15)	760 (14)	637 (15)	671 (15
	LIKELIHOOD	808 (12)	846 (11)	910 (9)	760 (14)	843 (12)	882 (10)	764 (13)	802 (13)	842 (12
	SKEW-NORMAL	816 (13)	838 (12)	906 (9)	773 (13)	855 (11)	867 (11)	776 (13)	797 (13)	822 (12
	CORRECTED SCORE	785 (13)	823 (12)	884 (10)	743 (14)	817 (12)	806 (13)	762 (14)	786 (13)	793 (13
	CONDITIONAL SCORE	792 (13)	820 (12)	881 (10)	744 (14)	817 (12)	804 (13)	766 (14)	787 (13)	799 (13
	SIMEX	796 (13)	832 (12)	892 (10)	755 (14)	833 (12)	821 (12)	784 (13)	809 (12)	823 (12
0.9			Normal			Mixture			Skew-Normal	
	NAIVE	778 (13)	635 (15)	625 (15)	634 (15)	755 (14)	700 (14)	735 (14)	696 (15)	649 (15
	LIKELIHOOD	790 (13)	858 (11)	894 (10)	779 (13)	817 (12)	866 (11)	769 (13)	823 (12)	851 (11
	SKEW-NORMAL	794 (13)	864 (11)	884 (10)	787 (13)	838 (12)	860 (11)	775 (13)	821 (12)	836 (12
	CORRECTED SCORE	782 (13)	829 (12)	863 (11)	766 (13)	801 (13)	811 (12)	741 (14)	793 (13)	816 (12
	CONDITIONAL SCORE	781 (13)	834 (12)	860 (11)	770 (13)	803 (13)	814 (12)	741 (14)	790 (13)	816 (12
	SIMEX	799 (13)	841 (12)	881 (10)	776 (13)	815 (12)	828 (12)	794 (13)	799 (13)	830 (12
1.5			Normal			Mixture			Skew-Normal	
	NAIVE	697 (15)	639 (15)	661 (15)	745 (14)	585 (16)	581 (16)	711 (14)	683 (15)	510 (16
	LIKELIHOOD	818 (67)	865 (11)	883 (10)	759 (14)	853 (11)	875 (10)	782 (13)	836 (12)	853 (11
	SKEW-NORMAL	812 (69)	865 (11)	878 (10)	764 (13)	859 (11)	867 (11)	801 (13)	845 (11)	844 (11
	CORRECTED SCORE	818 (67)	863 (15)	871 (11)	760 (14)	832 (12)	846 (11)	770 (13)	822 (12)	822 (12
	CONDITIONAL SCORE	818 (67)	863 (15)	869 (11)	758 (14)	835 (12)	841 (12)	770 (13)	824 (12)	819 (12
